# Asymmetric synthesis of γ-chloro-α,β-diamino- and β,γ-aziridino-α-aminoacylpyrrolidines and -piperidines via stereoselective Mannich-type additions of *N*-(diphenylmethylene)glycinamides across α-chloro-*N*-sulfinylimines

**DOI:** 10.3762/bjoc.8.239

**Published:** 2012-12-05

**Authors:** Gert Callebaut, Sven Mangelinckx, Pieter Van der Veken, Karl W Törnroos, Koen Augustyns, Norbert De Kimpe

**Affiliations:** 1 Department of Sustainable Organic Chemistry and Technology, Faculty of Bioscience Engineering, Ghent University, Coupure Links 653, B-9000 Ghent, Belgium, Tel: +32 (0)9 264 59 51. Fax: +32 (0)9 264 62 21; 2 Department of Medicinal Chemistry, University of Antwerp, Universiteitsplein 1, B-2610 Antwerp, Belgium; 3 Department of Chemistry, University of Bergen, Allégt. 41, N-5007 Bergen, Norway

**Keywords:** asymmetric synthesis, diaminoacid derivatives, Mannich-type addition, *N*-sulfinylimines, stereoselectivity

## Abstract

The asymmetric synthesis of new chiral γ-chloro-α,β-diaminocarboxylamide derivatives by highly diastereoselective Mannich-type reactions of *N*-(diphenylmethylene)glycinamides across chiral α-chloro-*N*-*p*-toluenesulfinylaldimines was developed. The resulting (*S*_S_,2*S*,3*S*)-γ-chloro-α,β-diaminocarboxylamides were formed with the opposite enantiotopic face selectivity as compared to the (*S*_S_,2*R*,3*R*)-γ-chloro-α,β-diaminocarboxyl esters obtained via Mannich-type addition of analogous *N*-(diphenylmethylene)glycine esters across a chiral α-chloro-*N*-*p*-toluenesulfinylaldimine. Selective deprotection under different acidic reaction conditions and ring closure of the γ-chloro-α,β-diaminocarboxylamides was optimized, which resulted in N^α^-deprotected *syn*-γ-chloro-α,β-diaminocarboxylamides, *N*-sulfinyl-β,γ-aziridino-α-aminocarboxylamide derivatives, a *trans*-imidazolidine, and an N^α^,N^β^-deprotected *syn*-γ-chloro-α,β-diaminocarboxylamide.

## Introduction

In recent years, non-proteinogenic diaminocarboxylic acids have gained a lot of attention among organic chemists and biochemists [1–3]. This is due to the fact that these diaminocarboxylic acids are present as key structural fragments in biologically active compounds, and some are also bioactive as the free diaminocarboxylic acid derivative [1–6]. For example, α,γ-diaminoacylamides are known for their high potency and selectivity as dipeptidyl peptidase (DPP) inhibitors [7–9].

DPP IVs are proteases that specifically cleave off N-terminal dipeptides and are involved in the degradation of incretin hormones, including glucagon-like peptide-1 (GLP-1) and glucose-dependent insulinotropic polypeptide (GIP). GLP-1 is involved in the regulation of glucose homeostasis through stimulation of insulin secretion, inhibition of glucagon release, and delay of gastric emptying. It has been demonstrated that the presence of intravenous GLP-1 increases insulin secretion as a response to elevated glucose levels, and as such, GLP-1 can offer therapeutic benefits for patients with type 2 diabetes. Unfortunately, therapeutic application of GLP-1 is problematic due to the lack of oral activity and the rapid degradation by plasma DPP IV. Therefore, DPP IV inhibitors could offer a solution to this problem, as they can extend the duration of action of GLP-1 and prolong the beneficial effects [10–12].

**Figure 1:**
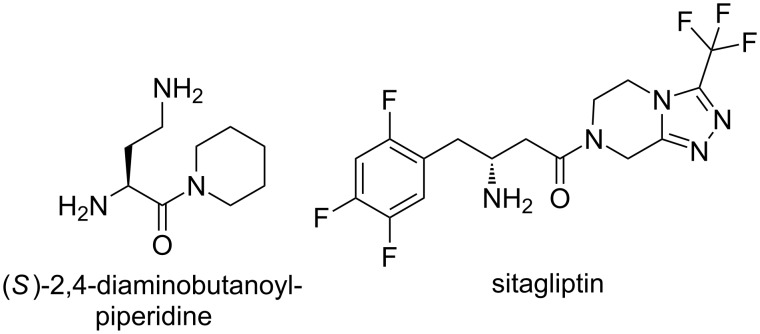
DPP inhibitors.

Besides DPP IV, a few related enzymes are present in the family of DPPs, with DPP II, DPP8, DPP9 and FAP being the most important regarding the therapeutic potential, when focusing on the inhibitory potency and selectivity [10–12]. In the research focused on DPP II and DPP IV inhibitors, it has been found that the α,γ-diaminoacylpiperidine, (*S*)-2,4-diaminobutanoylpiperidine, is a lead compound in the development of a large series of highly potent and selective DPP II inhibitors [7–9] (Figure 1). Next to the α,γ-diaminoacylpyrrolidines and –piperidines, which exhibit a DPP inhibitory effect, some β-aminocarboxylamides, such as sitagliptin, are also known as DPP inhibitors [13]. Sitagliptin is a commercialized oral antihyperglycemic drug of the DPP IV inhibitor class [14].

As α,γ-diaminocarboxylamides, as well as β-aminocarboxylamides, are known for their activity as DPP inhibitors, an increasing interest to study the DPP inhibitory potency of analogous α,β-diaminocarboxylamides exists [15]. The synthesis of chiral α,β-diaminocarboxylic acid derivatives by asymmetric Mannich-type addition of enolates across activated imines, e.g., *N*-sulfinylimines [16–20], is one of the most common and versatile methods in organic chemistry and is continuously under development [1–3]. Recently, our research group elaborated the asymmetric synthesis of new chiral γ-chloro-α,β-diaminocarboxyl esters by highly diastereoselective Mannich-type reactions of *N*-(diphenylmethylene)glycine esters across a chiral α-chloro-*N*-*p*-toluenesulfinylimine [20], which belongs to the useful class of α-halo-imines [21–26]. However, transformation of γ-chloro-α,β-diaminocarboxyl esters into the corresponding carboxylic acids, en route to further coupling to carboxylamides, has proven to be unsuccessful, probably due to competitive reactions such as the formation of α,β-diamino-γ-butyrolactones [20].

The results discussed within the present paper demonstrate the synthesis and elaboration of chiral *syn*-γ-chloro-α,β-diaminocarboxylamide derivatives with excellent diastereoselectivity. In order to develop potential DPP inhibitors, the ring closure and deprotection of the α-amino functionality of the synthesized γ-chloro-α,β-diaminocarboxylamides were explored as well.

## Results and Discussion

The stereoselective synthesis of chiral γ-chloro-α,β-diaminocarboxylamides was performed by using a Mannich-type addition of glycine amides **4** across chiral α-chloro-*N*-sulfinylaldimines **3**.

**Scheme 1:**
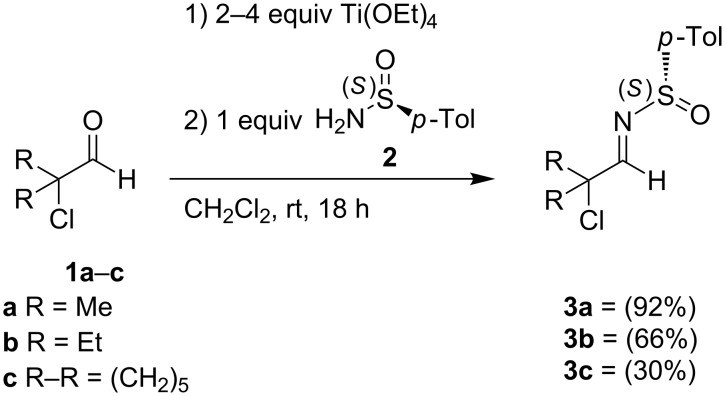
Synthesis of chiral α-chloro-*N*-*p*-toluenesulfinylaldimines **3**.

Initially, the chiral α-chloro-*N*-sulfinylaldimines **3**, including the new imines **3b** and **3c** derived from 2-chloro-2-ethylbutanal (**1b**) and 1-chlorocyclohexanecarboxaldehyde (**1c**), respectively, were efficiently prepared by condensation of α-chloroaldehydes **1** with (*S*)-(+)-*p*-toluenesulfinamide (**2**) in dichloromethane in the presence of Ti(OEt)_4_ (Scheme 1) [27].

**Scheme 2:**
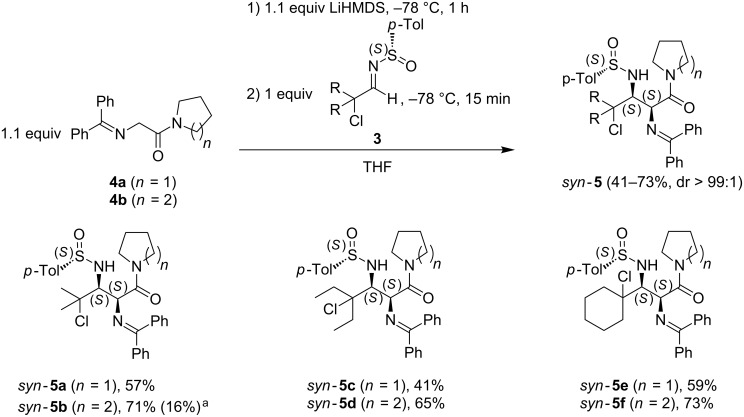
Synthesis of (*S*_S_,2*S*,3*S*)-γ-chloro-α,β-diaminocarboxylamides **5**. ^a^Yield in parentheses results from the use of LDA instead of LiHMDS.

The synthesis of *N*-(diphenylmethylene)glycinamides **4** was performed starting from *N*-Boc glycine, in accordance with literature procedures [28–29]. Based on our previously reported Mannich-type addition of glycine esters across chiral α-chloro-*N*-*p*-toluenesulfinylaldimine **3a** [20], the influence of the base (LiHMDS or LDA) used for the deprotonation of glycine amides **4** on the *syn*- or *anti*-selectivity of the Mannich-type addition was investigated (Scheme 2).

Initially, the Mannich-type addition of glycine amide **4b** across chiral α-chloro-*N*-*p*-toluenesulfinylisobutyraldimine (**3a**) was performed at −78 °C using 1.1 equiv of LDA. Upon ^1^H NMR analysis of the crude reaction mixture, the resulting *syn*-γ-chloro-α,β-diaminocarboxylamide *syn-***5b** was formed with an excellent stereoselectivity (dr > 99:1) but the conversion was rather low. After crystallization, the *syn*-adduct *syn-***5b** was isolated in a low yield of 16%. Repeating the Mannich-type addition of glycinamides **4** across chiral α-chloro-*N*-*p*-toluenesulfinylaldimines **3** with 1.1 equiv of LiHMDS resulted also in the formation of *syn*-γ-chloro-α,β-diaminocarboxylamides *syn-***5** with an excellent stereoselectivity (dr > 99:1). Because of the complete conversion of the substrates under these better reaction conditions (−78 °C, 15 min), the *syn*-adducts *syn*-**5** could be isolated in higher yields (41–73%) after recrystallization. The diastereomeric ratio of these *syn*-γ-chloro-α,β-diaminocarboxylamides *syn-***5** (dr > 99:1) was determined by a combination of ^1^H NMR, ^13^C NMR and HPLC analysis in which no signals from other diastereomers could be detected.

**Figure 2:**
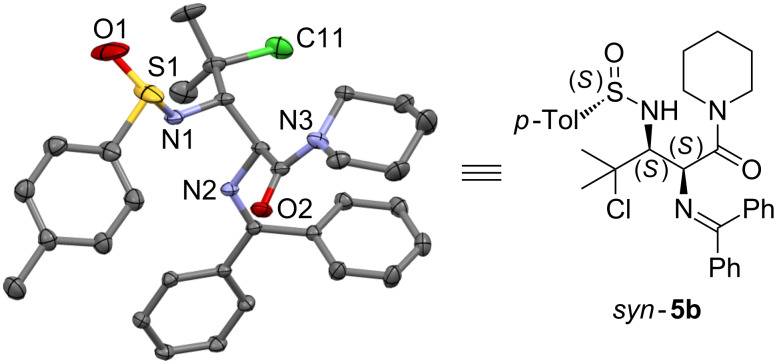
Crystal structure of *syn*-γ-chloro-α,β-diaminocarboxylamide *syn-***5b**.

In contrast to the Mannich-type addition of glycine esters across chiral α-chloro-*N*-*p*-toluenesulfinylimine **3a** [20], the diastereoselectivity of the Mannich-type addition of glycinamides **4** across chiral α-chloro-*N*-*p*-toluenesulfinylaldimines **3** was independent of the base used. The absolute stereochemistry of (*S*_S_,2*S*,3*S*)-γ-chloro-α,β-diaminocarboxylamides *syn-***5** was unambiguously determined by means of an X-ray diffraction analysis of compound *syn*-**5b** (Figure 2), in combination with the analogous NMR chemical shifts (H_α_: δ = 4.91–5.20 ppm, H_β_: δ = 3.74–4.05 ppm) and the characteristic vicinal coupling constants (^3^*J*_Hα-Hβ_ = 0–1.1 Hz) of all derivatives *syn-***5a**–**f**.

The vicinal coupling constant ^3^*J*_Hα-Hβ_ = 0–1.1 Hz for the *syn*-amides **5** has a comparably small value as the observed vicinal coupling constant ^3^*J*_Hα-Hβ_ of the closely related *syn*-γ-chloro-α,β-diaminocarboxyl esters (^3^*J*_Hα-Hβ_ = 1.1 Hz) [20]. Notably, the (*S*_S_,2*S*,3*S*)-γ-chloro-α,β-diaminocarboxylamides *syn-***5** were obtained with the opposite enantioselectivity as compared to the (*S*_S_,2*R*,3*R*)-γ-chloro-α,β-diaminocarboxyl esters obtained by Mannich-type addition of *E*-enolates derived from glycine esters across imines **3** [20].

**Scheme 3:**
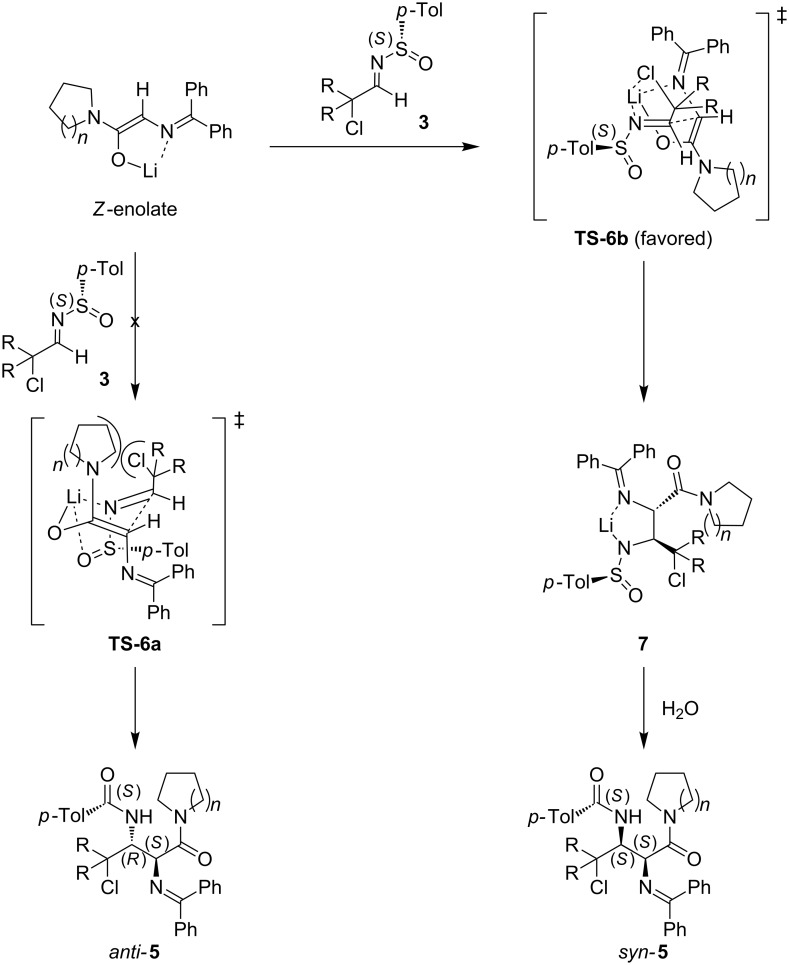
Transition-state model for reaction of the *Z*-enolate of glycinamides **4** in the Mannich-type addition across chiral α-chloro-*N*-*p*-toluenesulfinyl aldimines **3**.

The monosubstituted tertiary amide enolates obtained by deprotonation of *N*-(diphenylmethylene)glycinamides **4** are expected to have the *Z*-geometry in which A(1,3) interactions are minimized and Li-chelation stabilizes the conformation (Scheme 3), regardless of the base that was used [30–31].

Reaction of the *Z*-enolates via a cyclic chelated six-membered chairlike transition-state model **TS-6a**, would have resulted in *anti*-addition products *anti*-**5** in analogy with our previously obtained results on the synthesis of (*S*_S_,2*S*,3*R*)-γ-chloro-α,β-diaminocarboxyl esters [20]. However, starting from glycinamides **4**, due to the important 1,3-diaxial interaction between the haloalkyl group (–CClR_2_) and the cyclic amine moiety [–N(CH_2_)*_n_*] in this transition state, **TS-6a** is highly disfavored. The formation of the (*S*_S_,2*S*,3*S*)-γ-chloro-α,β-diaminocarboxylamides *syn-***5** can be explained by a boatlike transition-state model **TS-6b** involving the (*E*)-*N*-*p*-toluenesulfinylaldimines **3** [32–35]. This less sterically hindered transition state **TS-6b**, in which the haloalkyl group (–CClR_2_) occupies the less hindered pseudoequatorial position, and the corresponding Li-adduct **7** are stabilized by the interaction between the Li-cation, the diphenylmethyleneamino group, and the sulfinylimine nitrogen.

**Scheme 4:**
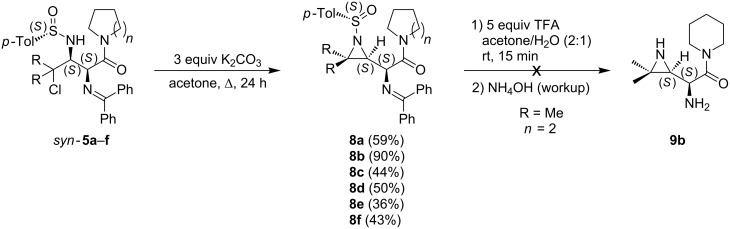
Synthesis of *N*-sulfinyl-β,γ-aziridino-α-amino carboxylic amides **8**.

The reversal of the enantiotopic face selectivity in the reaction of the *N*-sulfinylimines **3** with the glycinamides **4**, as compared to the reaction with glycine esters, is attributed to the α-coordinating ability of the chlorine atom with the lithium of the incoming enolate as depicted in transition state **TS-6b**. The coordinating α-chloro atom in **TS-6b** overrides the chelation of the sulfinyl oxygen (e.g., **TS-6a**) and allows the sulfinylimine to react in the conformation wherein the S=O bond and the lone pair of electrons on the nitrogen atom are antiperiplanar [36]. This reversal of stereoselectivity is analogous to results obtained with other *N*-*p*-toluenesulfinylimines containing an oxygen atom as α-coordinating group [37–39]. The resulting *syn*-addition products *syn-***5** were subsequently cyclized to the corresponding *N*-sulfinyl-β,γ-aziridino-α-aminocarboxylamides **8** upon treatment with K_2_CO_3_ in acetone under reflux in a moderate to very good yield (36–90%, Scheme 4).

The conversion of the ring-closure reaction was always complete as determined by TLC analysis, but purification of these *N*-sulfinyl-β,γ-aziridino-α-aminocarboxylamides **8** by flash chromatography resulted in a considerable loss of product.

In order to extend the potential applicability of the synthesized *N*-sulfinyl-β,γ-aziridino-α-aminocarboxylamides **8** as building blocks in biomedicinal chemistry, some attempts were made to remove the N-protective groups of diaminocarboxylamides **8** under mild acidic conditions (Scheme 4). In analogy with our recently published results on the corresponding aziridino esters [20], amide **8b** was treated with 5 equiv of trifluoroacetic acid in acetone/water (2:1) at rt for 15 min. After a basic workup with NH_4_OH, it was concluded that the conversion towards the N-deprotected *syn*-β,γ-aziridino-α-aminocarboxylamide **9b** was complete based on ^1^H NMR and LC–MS analysis of the crude reaction mixture. Unfortunately, all attempted purification techniques (column chromatography, preparative TLC, acid-base extraction) to remove benzophenone and some other minor impurities from the crude reaction mixture, failed to provide the pure N-deprotected *syn*-β,γ-aziridino-α-aminocarboxylamide **9b**.

**Scheme 5:**
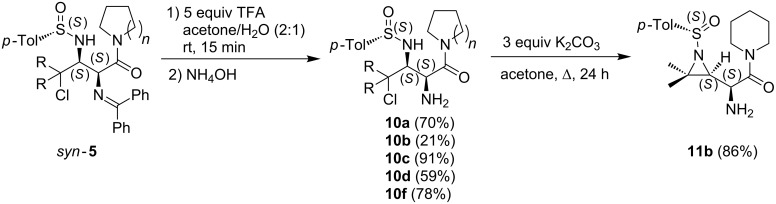
α**-**Deprotection and subsequent ring-closure of *syn*-γ-chloro-α,β-diamino carboxylic amides *syn-***5**.

Alternatively, the deprotection of the α-amino functionality of the synthesized *syn*-γ-chloro-α,β-diaminocarboxylamides *syn*-**5** was investigated en route towards the development of potential DPP inhibitors [7–9]. The *syn*-γ-chloro-α,β-diaminocarboxylamides *syn*-**5** were treated with 5 equiv of trifluoroacetic acid in acetone/water (2:1) for 15 min (Scheme 5).

After a basic workup with NH_4_OH, the α**-**deprotected *syn*-γ-chloro-α,β-diaminocarboxylamides **10** could be purified by crystallization or preparative TLC (21–91% yield). The obtained result was in accordance with the earlier reported selective deprotection of a benzophenone imine functionality, in the presence of an *N*-*p*-toluenesulfinyl moiety, of diamino esters with H_3_PO_4_/H_2_O/THF [17,40].

**Scheme 6:**
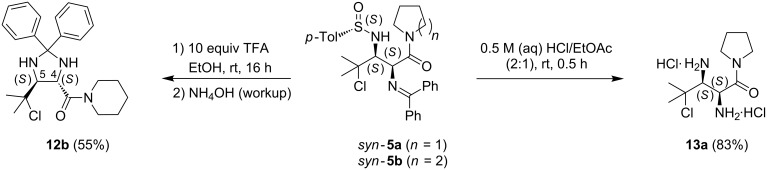
*N*-*p*-toluenesulfinyl-deprotection of *syn*-γ-chloro-α,β-diaminocarboxylamides *syn-***5**.

In a subsequent step, *syn*-γ-chloro-α,β-diaminocarboxylamide **10b** was chemoselectively cyclized to the corresponding *N*-sulfinyl-β,γ-aziridino-α-aminocarboxylamide **11b** upon treatment with K_2_CO_3_ in acetone under reflux in 86% yield. In order to provide access to the N^α^,N^β^-deprotected *syn*-γ-chloro-α,β-diaminocarboxylamides, *syn*-γ-chloro-α,β-diaminocarboxylamides *syn*-**5** were subjected to some alternative acidic deprotection reactions (Scheme 6).

In an initial reaction, *syn*-γ-chloro-α,β-diaminocarboxylamide *syn-***5b** was treated with 10 equiv of trifluoroacetic acid in ethanol at rt [18]. This resulted in *trans*-imidazolidine **12b** after basic workup with NH_4_OH. It is remarkable that the *N*-(diphenylmethylene) group was not removed under these reaction conditions but was trapped by the deprotected β-amino group, as the deprotection of analogous *anti*-substrates under the same reaction conditions led to unprotected *anti*-α,β-diaminocarboxyl esters [18]. This is possibly due to the fact that solvolysis of the imine functionality with ethanol is not favorable and an acid-catalyzed deprotection of the sulfinyl moiety will occur first [41–42]. The resulting β-amino deprotected *syn*-γ-chloro-α,β-diaminocarboxylamide could subsequently ring close further to *trans*-imidazolidine **12b**, which will be less sterically congested than an analogous *cis*-imidazolidine. In the literature, comparable non-halogenated *trans*-imidazolidines were already synthesized by 1,3-dipolar cycloaddition of *N*-benzylidene glycine ester enolates across *N*-sulfinylaldimines in the presence of a Lewis acid [43]. The *trans*-stereochemistry of imidazolidine **12b** was ensured by the vicinal coupling constant ^3^*J*_H4-H5_ = 7.43 Hz and the ^1^H NMR chemical shift of H4 (3.85 ppm), which were in the same range as for closely related *trans*-imidazolidines and *trans*-oxazolidines [43–45]. The *trans*-imidazolidine **12b** is a potential building block for foldamers, as the corresponding *trans*-oxazolidin-2-ones are already applied as such [46]. *trans*-Imidazolidine **12b** could also be used as a precursor of the corresponding N^α^,N^β^-deprotected α,β-diaminocarboxylamide, by hydrolysis under acidic conditions, in analogy with deprotection reactions of imidazolidines, imidazolines and oxazolines in the literature [16,47–48]. However, in a second reaction, *syn*-γ-chloro-α,β-diaminocarboxylamide *syn*-**5a** was directly converted into the dihydrochloride of the N^α^,N^β^-deprotected *syn*-γ-chloro-α,β-diaminocarboxylamide **13a**, by stirring in 0.5 M (aq) HCl/EtOAc (2:1) for 30 min at rt, in a yield of 83%. In this reaction, the acid-catalyzed hydrolysis of the benzophenone imine functionality proceeds readily and prevents the formation of the corresponding *trans*-imidazolidine.

## Conclusion

In conclusion, it was demonstrated that new chiral *syn*-γ-chloro-α,β-diaminocarboxylamides are formed in acceptable to good yields and with excellent diastereomeric ratios by stereoselective Mannich-type reactions of *N*-(diphenylmethylene)glycinamides across chiral α-chloro-*N*-*p*-toluenesulfinylaldimines. Notably, a very high *syn*-diastereoselectivity was obtained in the synthesis of the (*S*_S_,2*S*,3*S*)-γ-chloro-α,β-diaminocarboxylamides with the opposite enantiotopic face selectivity as compared to the Mannich-type additions of *N*-(diphenylmethylene)glycine esters across chiral α-chloro-*N*-*p*-toluenesulfinylaldimines. The synthesized γ-chloro-α,β-diaminocarboxylamides were selectively deprotected under acidic conditions, and the resulting α,β-diaminoacylpyrrolidines and -piperidines have a potential applicability as dipeptidyl peptidase inhibitors, which is currently under investigation.

## Supporting Information

File 1 General experimental conditions, experimental procedures and data, copies of ^1^H NMR and ^13^C NMR spectra for compounds **3**, *syn-***5**, **8**, and **10**–**13**.

File 2 CIF-file of compound *syn-***5b**.
