# Supplementary material for: Asymmetric synthesis of γ-chloro-α,β-diamino- and β,γ-aziridino-α-aminoacylpyrrolidines and -piperidines via stereoselective Mannich-type additions of N-(diphenylmethylene)glycinamides across α-chloro-N-sulfinylimines
Source: Beilstein J Org Chem. 2012 Dec 5;8:2124–31. doi: 10.3762/bjoc.8.239 (PMC3520569; doi:10.3762/bjoc.8.239)
Supplement: File 1 — General experimental conditions, experimental procedures and data, copies of 1H NMR and 13C NMR spectra for compounds 3, syn-5, 8, and 10–13. [file Beilstein_J_Org_Chem-08-2124-s001.pdf]

Supporting Information File 1

for

Asymmetric synthesis of γ-chloro-α,β-diamino- and β,γ-aziridino-α-aminoacylpyrrolidines and -piperidines via stereoselective Mannich-type additions of *N*-(diphenylmethylene)glycinamides across α-chloro-*N*-sulfinylimines

Gert Callebaut[1,§], Sven Mangelinckx[1,‡], Pieter Van der Veken[2], Karl W. Törnroos[3], Koen Augustyns[2] and Norbert De Kimpe[1,*]

Address: [1]Department of Sustainable Organic Chemistry and Technology, Faculty of Bioscience Engineering, Ghent University, Coupure Links 653, B-9000 Ghent, Belgium, Tel: +32 (0)9 264 59 51. Fax: +32 (0)9 264 62 21; [2]Department of Medicinal Chemistry, University of Antwerp, Universiteitsplein 1, B-2610 Antwerp, Belgium and [3]Department of Chemistry, University of Bergen, Allégt. 41, N-5007 Bergen, Norway

Email: Norbert De Kimpe* - norbert.dekimpe@UGent.be

*Corresponding author

[§]Aspirant of the "Institute for the Promotion of Innovation through Science and Technology – Flanders (IWT-Vlaanderen)".

[‡]Postdoctoral Fellow of the Research Foundation (FWO).

General experimental conditions, experimental procedures and data, copies of ^{1}H NMR and ^{13}C NMR spectra for compounds 3, *syn*-5, 8, and 10–13

Table of contents

I. General experimental methods S2

II. Experimental methods……………………………………......…………….........S3

III. Copies of ^{1}H NMR and ^{13}C NMR spectra of **3**, *syn*-**5, 8** and **10–13**...…………….........S16

I. General experimental methods

Flame-dried glassware was used for all nonaqueous reactions. Commercially available solvents and reagents were purchased from common chemical suppliers and used without further purification, unless stated otherwise. Tetrahydrofuran (THF) was freshly distilled under a nitrogen atmosphere from sodium/benzophenone ketyl. Petroleum ether refers to the 40–60 °C boiling fraction. ^{1}H NMR (300 MHz), ^{13}C NMR (75 MHz) spectra were recorded in deuterated solvents with tetramethylsilane (TMS, $\delta = 0$ ppm) as internal standard unless specified otherwise. Mass spectra were recorded by using a direct inlet system (ESI, 4000 V). IR spectra were obtained from samples in neat form with an ATR (Attenuated Total Reflectance) accessory. HRMS analysis was performed by using an HPLC coupled to a time-of-flight mass spectrometer equipped with an ESI/APCI-multimode source. Melting points of crystalline compounds were determined in open-end capillary tubes by using a hot stage apparatus and were not corrected. The purification of the reaction mixtures was performed by column chromatography with silica gel (particle size 0.035–0.070 mm, pore diameter ca. 6 nm). Thin-layer chromatography (TLC) was performed on glass plates coated with silica gel 60 F_{254}, by using UV and $KMnO_4$ as a visualizing agent. (S_S)-*p*-Toluenesulfinamide is commercially available (>98% ee).

II. Experimental methods

Synthesis of (S_S)-α-chloro-*N*-*p*-toluenesulfinylaldimines (3). The synthesis of (S_S)-*N*-(2-chloro-2-ethylbutylidene)-*p*-toluenesulfinamide (**3b**) is representative. To a flame-dried round-bottomed flask, charged with 2-chloro-2-ethylbutanal (**1b**, 4.00 g, 29.72 mmol) in dry CH_2Cl_2 (100 mL), was added $Ti(OEt)_4$ (2 equiv, 13.60 g, 59.61 mmol) and (S_S)-*p*-toluenesulfinamide (**2**, 4.60 g, 29.64 mmol) under a nitrogen atmosphere. The reaction mixture was stirred for 18 h at rt. After completion, the reaction mixture was poured into H_2O:CH_2Cl_2 1/1 (200 mL) while being rapidly stirred. The suspension was filtered over Celite® and the solids were washed with CH_2Cl_2 (2 × 50 mL). Subsequently, the combined organic phases were dried ($MgSO_4$), filtered and evaporated in vacuo. The crude product was purified by column chromatography to yield 5.27 g (19.39 mmol, 66%) of pure (S_S)-*N*-(2-chloro-2-ethylbutylidene)-*p*-toluenesulfinamide (**3b**).

(S_S)-*N*-(2-Chloro-2-ethylbutylidene)-*p*-toluenesulfinamide (3b). R_f 0.35 (petroleum ether:EtOAc 9/1). Yellow oil, yield 66% (5.27 g). $[\alpha]_D$ +264.4 (*c* 1.1, $CHCl_3$). IR (cm^{-1}): ν_{max} 808, 1075, 1099, 1144, 1617. ^1H NMR (300 MHz, $CDCl_3$) δ 0.87 (3H, t, *J* = 7.15 Hz), 0.97 (3H, t, *J* = 7.15 Hz), 1.89-2.13 (4H, m), 2.40 (3H, s), 7.30 (2H, d, *J* = 8.26 Hz), 7.55 (2H, d, *J* = 8.26 Hz), 8.11 (1H, s). ^{13}C NMR (75 MHz, $CDCl_3$) δ 8.7, 8.8, 21.5, 32.0, 32.1, 76.1, 124.7 (2C), 130.0 (2C), 141.3, 142.1, 166.2. MS (ESI^+) *m/z* (%): 263 (100), 272/274 $[M + H]^+$, 60). HRMS (ESI) calcd for $C_{13}H_{18}ClNOS$: 272.0870 MH^+; found, 272.0864.

(S_S)-*N*-(1-Chlorocyclohexylmethylidene)-*p*-toluenesulfinamide (3c). R_f 0.31 (petroleum ether:EtOAc 9/1). Colourless oil, yield 30% (0.80 g). $[\alpha]_D$ +251.0 (*c* 1.2, $CHCl_3$). IR (cm^{-1}): ν_{max} 809, 1075, 1098, 1144, 1618. ^1H NMR (300 MHz, $CDCl_3$) δ 1.18-1.35 (1H, m), 1.49-2.13 (9H, m), 2.41 (3H, s), 7.31 (2H, d, *J* = 8.26 Hz), 7.55 (2H, d, *J* = 8.26 Hz), 8.15 (1H, s).

^{13}C NMR (75 MHz, $CDCl_3$) δ 21.5, 22.0 (2C), 25.0, 36.6 (2C), 71.6, 124.8 (2C), 130.0 (2C), 141.3, 142.0, 166.0. MS (ESI$^+$) *m/z* (%): 177 (100), 284/286 [M + H$^+$, 40]. HRMS (ESI) calcd for $C_{14}H_{18}ClNOS$: 284.0870 MH$^+$; found, 284.0861.

Synthesis of (S_S,2*S*,3*S*)-*N*-[4-chloro-2-(diphenylmethyleneamino)-3-(*p*-toluenesulfinyl-amino)alkanoyl]amines *syn*-5. The synthesis of (S_S,2*S*,3*S*)-*N*-[4-chloro-2-(diphenylmethyleneamino)-4-methyl-3-(*p*-toluenesulfinylamino)pentanoyl]piperidine (*syn*-**5b**) is representative. A solution of *N*-[2-(diphenylmethyleneamino)acetyl]piperidine (**4b**, 1.1 equiv, 0.91 mmol, 0.28 g) in THF (10 mL) was cooled to −78 °C under a nitrogen atmosphere. A 1.0 M solution of LiHMDS (1.1 equiv, 0.91 mL, 0.91 mmol) in THF was slowly added and the resulting solution was stirred for 1 h at −78 °C. After deprotonation, a solution of (S_S)-α-chloro-*N*-*p*-toluenesulfinyl isobutyraldimine (**3a**, 1.0 equiv, 0.82 mmol, 0.20 g) in THF (5 mL) was added dropwise and the reaction mixture was stirred at −78 °C for 15 min. To the reaction mixture was added a saturated solution of NH_4Cl (5 mL) while being stirred at −78 °C for 2 min. The reaction mixture was brought to rt followed by an extraction with EtOAc (3 × 20 mL). The combined organic phases were dried ($MgSO_4$), filtered and evaporated in vacuo. The crude product was purified by recrystallization from diethyl ether to yield 0.32 g (0.58 mmol, 71%) of pure (S_S,2*S*,3*S*)-*N*-[4-chloro-2-(diphenylmethyleneamino)-4-methyl-3-(*p*-toluenesulfinylamino)pentanoyl]-piperidine (*syn*-**5b**).

(S_S,2*S*,3*S*)-*N*-[4-Chloro-2-(diphenylmethyleneamino)-4-methyl-3-(*p*-toluenesulfinyl-amino)pentanoyl]pyrrolidine (*syn*-5a). White crystals, yield 57% (0.30 g). $[\alpha]_D$ +64.7 (*c* 1.3, $CHCl_3$). Mp 132.3 ± 2.0 °C. IR (cm^{-1}): ν_{max} 697, 703, 1069, 1096, 1277, 1424, 1444, 1635, 3291 (weak). ^{1}H NMR (300 MHz, $CDCl_3$) δ 1.45 (3H, s), 1.53-1.86 (4H, m), 1.77 (3H,

s), 2.25-2.35 (1H, m), 2.41 (3H, s), 3.26-3.40 (3H, m), 3.77 (1H, d, *J* = 9.36 Hz), 4.93 (1H, s), 6.16 (1H, d, *J* = 9.36 Hz), 7.12-7.19 (2H, m), 7.23-7.35 (4H, m), 7.35-7.50 (4H, m), 7.50-7.57 (2H, m), 8.07 (2H, d, *J* = 8.26 Hz). ^{13}C NMR (75 MHz, $CDCl_3$) δ 21.4, 24.0, 26.0, 27.3, 32.4, 46.1, 46.2, 62.5, 66.6, 73.6, 126.1 (2C), 126.8 (2C), 128.1 (2C), 128.5 (2C), 128.6, 128.7 (2C), 129.6 (2C), 130.7, 137.8, 138.5, 141.2, 143.8, 169.5, 170.1. MS (ESI$^+$) *m/z* (%): 536/538 [M + H$^+$, 100]. HRMS (ESI) calcd for $C_{30}H_{34}ClN_3O_2S$: 536.2133 MH$^+$; found, 536.2146.

(S_S,2*S*,3*S*)-*N*-[4-Chloro-2-(diphenylmethyleneamino)-4-methyl-3-(*p*-toluenesulfinyl-amino)pentanoyl]piperidine (*syn*-5b). White crystals, yield 71% (0.32 g). $[\alpha]_D$ +78.1 (*c* 0.9, $CHCl_3$). Mp 125.4 ± 2.0 °C. IR (cm^{-1}): ν_{max} 700, 1070, 1098, 1222, 1442, 1640, 3355 (weak). ^{1}H NMR (300 MHz, $CDCl_3$) δ 0.90 (1H, m), 1.30-1.59 (5H, m), 1.47 (3H, s), 1.76 (3H, s), 2.41 (3H, s), 3.08-3.36 (3H, m), 3.52-3.64 (1H, m), 3.78 (1H, d, *J* = 9.36 Hz), 5.12 (1H, s), 6.04 (1H, d, *J* = 9.36 Hz), 7.12-7.18 (2H, m), 7.23-7.50 (8H, m), 7.53 (2H, d, *J* = 8.26 Hz), 7.95 (2H, d, *J* = 8.26 Hz). ^{13}C NMR (75 MHz, $CDCl_3$) δ 21.4, 24.4, 25.4, 25.8, 27.5, 32.6, 43.3, 46.1, 61.3, 66.5, 73.7, 126.0 (2C), 127.1 (2C), 128.0 (2C), 128.57 (3C), 128.63 (2C), 129.6 (2C), 130.6, 137.4, 138.7, 141.3, 143.6, 169.2, 170.0. MS (ESI$^+$) *m/z* (%): 550/552 [M + H$^+$, 100]. HRMS (ESI) calcd for $C_{31}H_{36}ClN_3O_2S$: 550.2290 MH$^+$; found, 550.2306.

(S_S,2*S*,3*S*)-*N*-[4-Chloro-2-(diphenylmethyleneamino)-4-ethyl-3-(*p*-toluenesulfinylamino)-hexanoyl]pyrrolidine (*syn*-5c). White crystals, yield 41% (0.71 g). $[\alpha]_D$ +60.4 (*c* 2.7, $CHCl_3$). Mp 143.3 ± 1.0 °C. IR (cm^{-1}): ν_{max} 699, 1073, 1103, 1294, 1422, 1442, 1636, 3348 (weak). ^{1}H NMR (300 MHz, $CDCl_3$) δ 0.93 (3H, t, *J* = 7.43 Hz), 1.12 (3H, t, *J* = 7.15 Hz), 1.51-2.20 (8H, m), 2.24-2.35 (1H, m), 2.41 (3H, s), 3.24-3.38 (3H, m), 4.00 (1H, d, *J* = 9.36 Hz), 4.91 (1H, s), 6.12 (1H, d, *J* = 9.36 Hz), 7.13-7.22 (2H, m), 7.23-7.35 (4H, m), 7.36-

7.58 (6H, m), 8.12 (2H, d, *J* = 8.26 Hz). ^{13}C NMR (75 MHz, $CDCl_3$) δ 8.7, 8.9, 21.5, 24.0, 26.1, 29.4, 30.2, 46.2, 46.3, 62.2, 63.5, 83.0, 126.4 (2C), 126.9 (2C), 128.2 (2C), 128.7 (3C), 128.8 (2C), 129.7 (2C), 130.8, 137.9, 138.7, 141.2, 144.1, 169.7, 170.0. MS (ESI^+) *m/z* (%): 564/566 [M + H^+, 100]. HRMS (ESI) calcd for $C_{32}H_{38}ClN_3O_2S$: 564.2446 MH^+; found, 564.2454.

(*S*$_S$,2*S*,3*S*)-*N*-[4-Chloro-2-(diphenylmethyleneamino)-4-ethyl-3-(*p*-toluenesulfinylamino)-hexanoyl]piperidine (*syn*-5d). White crystals, yield 65% (0.33 g). $[\alpha]_D$ +61.1 (*c* 0.9, $CHCl_3$). Mp 138.5 ± 2.0 °C. IR (cm^{-1}): ν_{max} 701, 1074, 1111, 1252, 1439, 1634, 3332 (weak). ^{1}H NMR (300 MHz, $CDCl_3$) δ 0.83-1.04 (1H, m), 0.93 (3H, t, *J* = 7.15 Hz), 1.12 (3H, t, *J* = 7.15 Hz), 1.30-1.65 (5H, m), 1.72-2.16 (4H, m), 2.40 (3H, s), 2.98-3.09 (1H, m), 3.20-3.31 (1H, m), 3.39-3.47 (2H, m), 4.05 (1H, d, *J* = 9.36 Hz), 5.09 (1H, s), 5.95 (1H, d, *J* = 9.36 Hz), 7.12-7.17 (2H, m), 7.24-7.31 (4H, m), 7.35-7.49 (4H, m), 7.54 (2H, d, *J* = 8.26 Hz), 8.00 (2H, d, *J* = 8.26 Hz). ^{13}C NMR (75 MHz, $CDCl_3$) δ 8.6, 8.9, 21.5, 24.5, 25.6, 26.1, 29.4, 30.3, 43.4, 46.1, 61.2, 63.1, 83.3, 126.2 (2C), 127.2 (2C), 128.1 (2C), 128.7 (5C), 129.7 (2C), 130.7, 137.5, 138.9, 141.3, 143.9, 169.7 (2C). MS (ESI^+) *m/z* (%): 578/580 [M + H^+, 100]. HRMS (ESI) calcd for $C_{33}H_{40}ClN_3O_2S$: 578.2603 MH^+; found, 578.2609.

(*S*$_S$,2*S*,3*S*)-*N*-[3-(1-Chlorocyclohexyl)-2-(diphenylmethyleneamino)-3-(*p*-toluenesulfinyl-amino)propanoyl]pyrrolidine (*syn*-5e). White crystals, yield 59% (1.14 g). $[\alpha]_D$ +83.5 (*c* 2.5, $CHCl_3$). Mp 138.8 ± 2.0 °C. IR (cm^{-1}): ν_{max} 696, 704, 1068, 1092, 1294, 1430, 1634, 3309 (weak). ^{1}H NMR (300 MHz, $CDCl_3$) δ 1.05-1.21 (1H, m), 1.48-1.94 (12H, m), 2.24-2.47 (2H, m), 2.41 (3H, s), 3.27-3.40 (3H, m), 3.74 (1H, d, *J* = 9.36 Hz), 5.01 (1H, s), 6.16 (1H, d, *J* = 9.36 Hz), 7.12-7.18 (2H, m), 7.24-7.35 (4H, m), 7.36-7.56 (6H, m), 8.10 (2H, d, *J* = 8.26 Hz). ^{13}C NMR (75 MHz, $CDCl_3$) δ 21.5, 21.7, 22.1, 24.0, 24.8, 26.1, 33.7, 37.2, 46.2,

46.3, 62.3, 67.7, 80.1, 126.2 (2C), 126.9 (2C), 128.2 (2C), 128.6 (3C), 128.8 (2C), 129.7 (2C), 130.8, 137.9, 138.7, 141.3, 143.9, 169.7, 169.8. MS (ESI^+) *m/z* (%): 576/578 [M + H^+, 100]. HRMS (ESI) calcd for $C_{33}H_{38}ClN_3O_2S$: 576.2446 MH^+; found, 576.2436.

(S_S,2*S*,3*S*)-*N*-[3-(1-Chlorocyclohexyl)-2-(diphenylmethyleneamino)-3-(*p*-toluenesulfinyl-amino)propanoyl]piperidine (*syn*-5f). White crystals, yield 73% (1.20 g). $[\alpha]_D$ +86.9 (*c* 2.9, $CHCl_3$). Mp 132.2 ± 1.0 °C. IR (cm^{-1}): ν_{max} 701, 1073, 1104, 1221, 1441, 1638, 3320 (weak). ^1H NMR (300 MHz, $CDCl_3$) δ 0.82-0.98 (1H, m), 1.05-1.21 (1H, m), 1.31-1.81 (13H, m), 2.30-2.45 (1H, m), 2.41 (3H, s), 3.06-3.16 (1H, m), 3.18-3.38 (2H, m), 3.48-3.59 (1H, m), 3.76 (1H, d×d, *J* = 9.36 Hz, 1.10 Hz), 5.20 (1H, d, *J* = 1.10 Hz), 6.03 (1H, d, *J* = 9.36 Hz), 7.12-7.17 (2H, m), 7.24-7.56 (10H, m), 7.99 (2H, d, *J* = 8.26 Hz). ^{13}C NMR (75 MHz, $CDCl_3$) δ 21.5, 21.7, 22.1, 24.5, 24.8, 25.5, 25.9, 33.8, 37.4, 43.4, 46.2, 61.1, 67.8, 80.4, 126.1 (2C), 127.2 (2C), 128.1 (2C), 128.7 (5C), 129.7 (2C), 130.6, 137.5, 138.9, 141.4, 143.7, 169.5, 169.6. MS (ESI^+) *m/z* (%): 590/592 [M + H^+, 100]. HRMS (ESI) calcd for $C_{34}H_{40}ClN_3O_2S$: 590.2603 MH^+; found, 590.2609.

Synthesis of (S_S,2*S*,2′*S*)-*N*-{2-amino-2-[1-(*p*-toluenesulfinyl)aziridin-2-yl]acetyl}amines 8 and 11a. The synthesis of (S_S,2*S*,2′*S*)-*N*-{2-(diphenylmethyleneamino)-2-[3,3-dimethyl-1-(*p*-toluenesulfinyl)aziridin-2-yl]acetyl}piperidine (**8b**) is representative. To a solution of (S_S,2*S*,3*S*)-*N*-[4-chloro-2-(diphenylmethyleneamino)-4-methyl-3-(*p*-toluenesulfinylamino)-pentanoyl]-piperidine (*syn*-**5b**, 2.10 g, 3.82 mmol) in acetone (40 mL) was added K_2CO_3 (3.0 equiv, 11.45 mmol, 1.58 g) at rt. The reaction mixture was stirred for 24 h under reflux. After 24 h, the K_2CO_3 was filtered off and the solvent was evaporated in vacuo. The resulting oil was redissolved in EtOAc (40 mL) and washed with water (2 × 15 mL). The organic phase was dried ($MgSO_4$), filtered and evaporated in vacuo. The crude product was purified by

column chromatography to yield 1.75 g (3.41 mmol, 90%) of (S_S,2S,2′S)-*N*-[2-(diphenylmethyleneamino)-2-[3,3-dimethyl-1-(*p*-toluenesulfinyl)aziridin-2-yl]acetyl]piperidine (**8b**).

(S_S,2S,2′S)-*N*-{2-(Diphenylmethyleneamino)-2-[3,3-dimethyl-1-(*p*-toluenesulfinyl)-aziridin-2-yl]acetyl}pyrrolidine (8a). R_f 0.09 (petroleum ether:EtOAc 1/1). Yellow oil, yield 59% (0.33 g). $[\alpha]_D$ +37.4 (*c* 4.1, $CHCl_3$). IR (cm^{-1}): ν_{max} 697, 1072, 1093, 1278, 1444, 1638. ^{1}H NMR (300 MHz, $CDCl_3$) δ 1.15 (3H, s), 1.60-1.78 (4H, m), 1.69 (3H, s), 1.98 (3H, s), 2.80 (2H, t, *J* = 6.3 Hz), 3.34-3.51 (2H, m), 3.40 (1H, d, *J* = 8.81 Hz), 4.05 (1H, d, *J* = 8.26 Hz), 6.74 (2H, d, *J* = 7.71 Hz), 7.07-7.15 (2H, m), 7.28-7.49 (8H, m), 7.57 (2H, d, *J* = 8.26 Hz). ^{13}C NMR (75 MHz, $CDCl_3$) δ 20.8, 21.5, 22.7, 23.8, 26.1, 46.0, 46.4, 48.0, 50.7, 64.7, 124.9 (2C), 127.9 (2C), 128.1 (2C), 129.05, 129.14 (2C), 129.4 (4C), 130.4, 135.1, 139.4, 141.8 (2C), 168.2, 169.6. MS (ESI^+) *m/z* (%): 500 [M + H^+, 100]. HRMS (ESI) calcd for $C_{30}H_{33}N_3O_2S$: 500.2366 MH^+; found, 500.2375.

(S_S,2S,2′S)-*N*-{2-(Diphenylmethyleneamino)-2-[3,3-dimethyl-1-(*p*-toluenesulfinyl)-aziridin-2-yl]acetyl}piperidine (8b). R_f 0.10 (petroleum ether:EtOAc 1/1). Yellow oil, yield 90% (1.75 g). $[\alpha]_D$ +43.8 (*c* 2.7, $CHCl_3$). IR (cm^{-1}): ν_{max} 696, 726, 1076, 1102, 1216, 1443, 1639. ^{1}H NMR (300 MHz, $CDCl_3$) δ 1.15 (3H, s), 1.17-1.31 (2H, m), 1.34-1.63 (4H, m), 1.70 (3H, s), 1.99 (3H, s), 2.83 (2H, br s), 3.19-3.31 (1H, m), 3.43 (1H, d, *J* = 8.26 Hz), 3.66-3.78 (1H, m), 4.15 (1H, d, *J* = 8.26 Hz), 6.74 (2H, d, *J* = 7.71 Hz), 7.11 (2H, d, *J* = 7.71 Hz), 7.25-7.49 (8H, m), 7.57 (2H, d, *J* = 7.71 Hz). ^{13}C NMR (75 MHz, $CDCl_3$) δ 20.7, 21.4, 22.4, 24.4, 25.6, 26.5, 43.4, 46.0, 47.7, 50.6, 63.0, 124.8 (2C), 127.7 (2C), 127.9 (2C), 129.0 (2C), 129.15, 129.21 (2C), 129.3 (2C), 130.3, 134.5, 139.4, 141.60, 141.64, 167.8, 169.3. MS

(ESI$^+$) *m/z* (%): 514 [M + H$^+$, 100]. Anal. calcd for $C_{31}H_{35}N_3O_2S$: 514.2523 MH$^+$; found, 514.2513.

(S_S,2*S*,2′*S*)-*N*-{2-(Diphenylmethyleneamino)-2-[3,3-diethyl-1-(*p*-toluenesulfinyl)aziridin-2-yl]-acetyl}pyrrolidine (8c). R_f 0.07 (petroleum ether:EtOAc 1/1). Yellow oil, yield 44% (0.12 g). $[\alpha]_D$ +71.6 (*c* 1.9, $CHCl_3$). IR (cm^{-1}): ν_{max} 696, 1076, 1102, 1277, 1427, 1444, 1644. ^{1}H NMR (300 MHz, $CDCl_3$) δ 0.79 (3H, t, *J* = 7.4 Hz), 1.04 (3H, t, *J* = 7.4 Hz), 1.08-1.22 (1H, m), 1.32-1.45 (1H, m), 1.53-1.70 (4H, m), 1.88 (3H, s), 1.90-2.10 (2H, m), 2.60-2.75 (2H, m), 3.24-3.42 (2H, m), 3.39 (1H, d, *J* = 8.26 Hz), 4.03 (1H, d, *J* = 8.26 Hz), 6.65 (2H, d, *J* = 7.71 Hz), 7.00-7.07 (2H, m), 7.19-7.45 (8H, m), 7.49 (2H, d, *J* = 8.26 Hz). ^{13}C NMR (75 MHz, $CDCl_3$) δ 9.3, 10.6, 21.4, 23.8, 24.0, 24.4, 26.0, 45.8, 46.2, 51.1, 56.1, 64.0, 124.9 (2C), 127.7 (2C), 127.9 (2C), 129.0, 129.1 (2C), 129.2 (2C), 129.3 (2C), 130.3, 135.2, 139.3, 141.6, 141.8, 168.2, 169.4. MS (ESI$^+$) *m/z* (%): 528 [M + H$^+$, 100]. Anal. calcd for $C_{32}H_{37}N_3O_2S$: 528.2679 MH$^+$; found, 528.2691.

(S_S,2*S*,2′*S*)-*N*-{2-(Diphenylmethyleneamino)-2-[3,3-diethyl-1-(*p*-toluenesulfinyl)aziridin-2-yl]-acetyl}piperidine (8d). R_f 0.08 (petroleum ether:EtOAc 1/1). Yellow oil, yield 50% (0.07 g). $[\alpha]_D$ +79.9 (*c* 2.8, $CHCl_3$). IR (cm^{-1}): ν_{max} 696, 1077, 1101, 1214, 1442, 1641. ^{1}H NMR (300 MHz, $CDCl_3$) δ 0.85 (3H, t, *J* = 7.4 Hz), 1.11 (3H, t, *J* = 7.4 Hz), 1.15-1.30 (3H, m), 1.37-1.60 (5H, m), 1.89-2.17 (2H, m), 1.96 (3H, s), 2.78-2.89 (2H, m), 3.18-3.31 (1H, m), 3.46 (1H, d, *J* = 8.26 Hz), 3.63-3.74 (1H, m), 4.19 (1H, d, *J* = 8.81 Hz), 6.73 (2H, d, *J* = 7.71 Hz), 7.12 (2H, d, *J* = 7.15 Hz), 7.27-7.45 (8H, m), 7.56 (2H, d, *J* = 7.15 Hz). ^{13}C NMR (75 MHz, $CDCl_3$) δ 9.4, 10.7, 21.5, 24.1, 24.3, 24.5, 25.7, 26.7, 43.5, 46.1, 51.1, 56.2, 62.6, 124.9 (2C), 127.8 (2C), 128.0 (2C), 129.1 (2C), 129.2, 129.3 (4C), 130.3, 134.8,

139.5, 141.7, 141.8, 167.9, 169.3. MS (ESI^+) *m/z* (%): 542 [M + H^+, 100]. HRMS (ESI) calcd for $C_{33}H_{39}N_3O_2S$: 542.2836 MH^+; found, 542.2846.

(S_S,2*S*,2′*S*)-*N*-{2-(Diphenylmethyleneamino)-2-[1-(*p*-toluenesulfinyl)-1-azaspiro[2.5]oct-2-yl]-acetyl}pyrrolidine (8e). R_f 0.21 (petroleum ether:EtOAc 2/1). Yellow oil, yield 36% (0.09 g). $[\alpha]_D$ +53.9 (*c* 2.5, $CHCl_3$). IR (cm^{-1}): ν_{max} 696, 751, 1075, 1096, 1260, 1431, 1444, 1638. ^1H NMR (300 MHz, $CDCl_3$) δ 1.14-1.77 (11H, m), 1.80-1.92 (1H, m), 1.94 (3H, s), 2.08 (2H, t, *J* = 6.05 Hz), 2.60-2.76 (2H, m), 3.32-3.50 (2H, m), 3.46 (1H, d, *J* = 8.81 Hz), 4.10 (1H, d, *J* = 8.26 Hz), 6.70 (2H, d, *J* = 7.71 Hz), 7.07-7.14 (2H, m), 7.28-7.50 (8H, m), 7.57 (2H, d, *J* = 8.26 Hz). ^{13}C NMR (75 MHz, $CDCl_3$) δ 21.5, 23.9, 24.8, 25.3, 25.7, 26.1, 32.0, 33.0, 45.8, 46.3, 51.0, 53.1, 63.9, 124.9 (2C), 127.8 (2C), 128.0 (2C), 129.0, 129.2 (2C), 129.3 (2C), 129.4 (2C), 130.3, 135.1, 139.4, 141.7, 141.8, 168.3, 169.4. MS (ESI^+) *m/z* (%): 540 [M + H^+, 100]. HRMS (ESI) calcd for $C_{33}H_{37}N_3O_2S$: 540.2679 MH^+; found, 540.2681.

(S_S,2*S*,2′*S*)-*N*-{2-(Diphenylmethyleneamino)-2-[1-(*p*-toluenesulfinyl)-1-azaspiro[2.5]oct-2-yl]-acetyl}piperidine (8f). R_f 0.13 (petroleum ether:EtOAc 2/1). Yellow oil, yield 43% (0.10 g). $[\alpha]_D$ +49.1 (*c* 2.3, $CHCl_3$). IR (cm^{-1}): ν_{max} 696, 751, 1075, 1099, 1218, 1443, 1639. ^1H NMR (300 MHz, $CDCl_3$) δ 1.10-1.75 (13H, m), 1.80-1.92 (1H, m), 1.95 (3H, s), 2.08 (2H, t, *J* = 6.05 Hz), 2.70-2.86 (2H, m), 3.20-3.31 (1H, m), 3.45 (1H, d, *J* = 8.26 Hz), 3.67-3.77 (1H, m), 4.18 (1H, d, *J* = 8.26 Hz), 6.70 (2H, d, *J* = 8.26 Hz), 7.08-7.16 (2H, m), 7.28-7.47 (8H, m), 7.56 (2H, d, *J* = 8.26 Hz). ^{13}C NMR (75 MHz, $CDCl_3$) δ 21.4, 24.4, 24.8, 25.2, 25.6 (2C), 26.6, 31.9, 32.7, 43.3, 46.0, 50.8, 53.1, 62.2, 124.8 (2C), 127.7 (2C), 127.9 (2C), 129.08 (2C), 129.14, 129.2 (4C), 130.2, 134.6, 139.4, 141.6, 141.7, 167.9, 169.1. MS (ESI^+) *m/z* (%): 554 [M + H^+, 100]. HRMS (ESI) calcd for $C_{34}H_{39}N_3O_2S$: 554.2836 MH^+; found, 554.2836.

(S_S,2*S*,2′*S*)-*N*-{2-Amino-2-[3,3-dimethyl-1-(*p*-toluenesulfinyl)aziridin-2-yl]acetyl}-piperidine (11b). R_f 0.10 (petroleum ether:EtOAc 1/1). Yellow oil, yield 86% (0.18 g). $[\alpha]_D$ +52.0 (*c* 2.1, $CHCl_3$). IR (cm^{-1}): ν_{max} 751, 813, 1065, 1088, 1221, 1444, 1631, 3272. ^1H NMR (300 MHz, $CDCl_3$) δ 1.07-1.36 (2H, m), 1.28 (3H, s), 1.40-1.71 (6H, m), 1.57 (3H, s), 2.42 (3H, s), 2.63 (1H, d, *J* = 8.26 Hz), 3.32 (1H, d, *J* = 8.26 Hz), 3.35-3.62 (4H, m), 7.34 (2H, d, *J* = 8.26 Hz), 7.72 (2H, d, *J* = 8.26 Hz). ^{13}C NMR (75 MHz, $CDCl_3$) δ 20.9, 21.5, 22.0, 24.4, 25.5, 26.6, 43.1, 46.5, 49.9, 51.0, 51.3, 125.3 (2C), 129.7 (2C), 142.7, 142.8, 169.9. MS (ESI^+) *m/z* (%): 350 [M + H^+, 100]. HRMS (ESI) calcd for $C_{18}H_{27}N_3O_2S$: 350.1897 MH^+; found, 350.1892.

Synthesis of (S_S,2*S*,3*S*)-*N*-[2-amino-4-chloro-3-(*p*-toluenesulfinylamino)alkanoyl]amines 10. The synthesis of (S_S,2*S*,3*S*)-*N*-[2-amino-4-chloro-4-methyl-3-(*p*-toluenesulfinylamino)-pentanoyl]piperidine (**10b**) is representative. To a solution of (S_S,2*S*,3*S*)-*N*-[4-chloro-2-(diphenylmethyleneamino)-4-methyl-3-(*p*-toluenesulfinylamino)pentanoyl]piperidine (*syn*-**5b**, 0.82 g, 1.49 mmol) in acetone/H_2O (2:1) (30 mL) was added dropwise trifluoroacetic acid (5 equiv, 7.45 mmol, 0.57 mL) at rt. The reaction mixture was stirred for 15 min at rt and subsequently quenched with NH_4OH in H_2O until pH 10 and concentrated in vacuo. The residue was redissolved in water (10 mL) and NH_4OH in H_2O was added until pH 10. The aqueous phase was extracted with CH_2Cl_2 (3 × 10 mL). The combined organic phases were dried ($MgSO_4$), filtered and evaporated in vacuo. The crude product was purified by crystallization from diethyl ether to yield 0.12 g (0.31 mmol, 21%) of pure (S_S,2*S*,3*S*)-*N*-[2-amino-4-chloro-4-methyl-3-(*p*-toluenesulfinylamino)pentanoyl]-piperidine (**10b**).

(S_S,2*S*,3*S*)-*N*-[2-Amino-4-chloro-4-methyl-3-(*p*-toluenesulfinylamino)pentanoyl]-pyrrolidine (10a). White crystals, yield 70% (0.26 g). $[\alpha]_D$ +51.0 (*c* 2.9, $CHCl_3$). Mp 125.4 ±

2.0 °C. IR (cm^{-1}): ν_{max} 892, 1062, 1083, 1343, 1451, 1599, 1629, 3230. ^{1}H NMR (300 MHz, $CDCl_3$) δ 1.55 (3H, s), 1.68 (3H, s), 1.77 (2H, br s), 1.84-2.13 (4H, m), 2.41 (3H, s), 3.44-3.59 (2H, m), 3.63 (2H, t, *J* = 6.6 Hz), 3.66 (1H, d×d, *J* = 9.6 Hz, 2.20 Hz), 4.24 (1H, d, *J* = 2.20 Hz), 4.89 (1H, d, *J* = 9.6 Hz), 7.32 (2H, d, *J* = 8.26 Hz), 7.83 (2H, d, *J* = 8.26 Hz). ^{13}C NMR (75 MHz, $CDCl_3$) δ 21.4, 24.0, 26.3, 29.2, 31.9, 46.6, 46.8, 52.4, 65.0, 73.7, 125.7 (2C), 129.6 (2C), 141.6, 142.7, 171.7. MS (ESI$^+$) *m/z* (%): 372/374 [M + H$^+$, 100]. HRMS (ESI) calcd for $C_{17}H_{26}ClN_3O_2S$: 372.1507 MH$^+$; found, 372.1513.

(*S*$_S$,2*S*,3*S*)-*N*-[2-Amino-4-chloro-4-methyl-3-(*p*-toluenesulfinylamino)pentanoyl]-piperidine (10b). White crystals, yield 21% (0.12 g). $[\alpha]_D$ +67.5 (*c* 2.4, $CHCl_3$). Mp 119.8 ± 2.0 °C. IR (cm^{-1}): ν_{max} 821, 854, 1066, 1092, 1244, 1446, 1460, 1613, 3181. ^{1}H NMR (300 MHz, $CDCl_3$) δ 1.50-1.77 (6H, m), 1.56 (3H, s), 1.69 (3H, s), 1.81 (2H, br s), 2.41 (3H, s), 3.46-3.58 (2H, m), 3.61 (1H, d×d, *J* = 9.91 Hz, 1.65 Hz), 3.67-3.81 (2H, m), 4.49 (1H, d, *J* = 1.65 Hz), 4.93 (1H, d, *J* = 9.91 Hz), 7.32 (2H, d, *J* = 8.0 Hz), 7.79 (2H, d, *J* = 8.0 Hz). ^{13}C NMR (75 MHz, $CDCl_3$) δ 21.4, 24.6, 25.6, 26.3, 28.7, 32.3, 44.0, 46.4, 50.4, 65.6, 73.8, 125.8 (2C), 129.6 (2C), 141.5, 142.7, 171.5. MS (ESI$^+$) *m/z* (%): 386/388 [M + H$^+$, 100]. HRMS (ESI) calcd for $C_{18}H_{28}ClN_3O_2S$: 386.1664 MH$^+$; found, 386.1676.

(*S*$_S$,2*S*,3*S*)-*N*-[2-Amino-4-chloro-4-ethyl-3-(*p*-toluenesulfinylamino)hexanoyl]pyrrolidine (10c). White crystals, yield 91% (0.16 g). $[\alpha]_D$ +49.4 (*c* 2.6, $CHCl_3$). Mp 127.3 ± 2.0 °C. IR (cm^{-1}): ν_{max} 637, 811, 1056, 1086, 1324, 1440, 1612, 1630, 3282. ^{1}H NMR (300 MHz, $CDCl_3$) δ 0.90 (3H, t, *J* = 7.15 Hz), 1.06 (3H, t, *J* = 7.15 Hz), 1.76 (2H, br s), 1.82-2.10 (8H, m), 2.41 (3H, s), 3.42-3.70 (4H, m), 3.90 (1H, d×d, *J* = 9.91 Hz, 1.65 Hz), 4.17 (1H, d, *J* = 1.65 Hz), 4.78 (1H, d, *J* = 9.91 Hz), 7.32 (2H, d, *J* = 8.26 Hz), 7.86 (2H, d, *J* = 8.26 Hz). ^{13}C NMR (75 MHz, $CDCl_3$) δ 8.7, 8.8, 21.5, 24.1, 26.4, 29.7, 30.5, 46.7, 46.8, 52.7, 61.3,

83.0, 125.8 (2C), 129.7 (2C), 141.6, 143.0, 171.8. MS (ESI$^+$) *m/z* (%): 400/402 [M + H$^+$, 100]. HRMS (ESI) calcd for $C_{19}H_{30}ClN_3O_2S$: 400.1820 MH$^+$; found, 400.1827.

(S_S,2*S*,3*S*)-*N*-[2-Amino-4-chloro-4-ethyl-3-(*p*-toluenesulfinylamino)hexanoyl]piperidine (10d). Yellow oil, yield 59% (0.07 g). $[\alpha]_D$ +41.8 (*c* 2.3, $CHCl_3$). IR (cm^{-1}): ν_{max} 751, 812, 1056, 1087, 1254, 1444, 1598, 1638, 3188. ^{1}H NMR (300 MHz, $CDCl_3$) δ 0.92 (3H, t, *J* = 7.15 Hz), 1.10 (3H, t, *J* = 7.15 Hz), 1.48-1.72 (6H, m), 1.76 (2H, br s), 1.85-2.12 (4H, m), 2.41 (3H, s), 3.36-3.53 (2H, m), 3.70-3.90 (2H, m), 3.85 (1H, d×d, *J* = 9.6 Hz, 1.65 Hz), 4.43 (1H, d, *J* = 1.65 Hz), 4.80 (1H, d, *J* = 9.6 Hz), 7.32 (2H, d, *J* = 8.0 Hz), 7.82 (2H, d, *J* = 8.0 Hz). ^{13}C NMR (75 MHz, $CDCl_3$) δ 8.7, 8.9, 21.5, 24.7, 25.7, 26.6, 29.6, 30.5, 44.0, 46.4, 50.6, 61.7, 83.0, 125.9 (2C), 129.7 (2C), 141.6, 142.9, 171.7. MS (ESI$^+$) *m/z* (%): 414/416 [M + H$^+$, 100]. HRMS (ESI) calcd for $C_{20}H_{32}ClN_3O_2S$: 414.1977 MH$^+$; found, 414.1980.

(S_S,2*S*,3*S*)-*N*-[2-Amino-3-(1-chlorocyclohexyl)-3-(*p*-toluenesulfinylamino)propanoyl]-piperidine (10f). Yellow crystals, yield 78% (0.28 g). $[\alpha]_D$ +73.2 (*c* 2.3, $CHCl_3$). Mp 95.7 ± 2.0 °C. IR (cm^{-1}): ν_{max} 813, 853, 889, 1068, 1092, 1243, 1444, 1634, 3165. ^{1}H NMR (300 MHz, $CDCl_3$) δ 1.05-2.20 (18H, m), 2.41 (3H, s), 3.44-3.59 (2H, m), 3.62 (1H, d×d, *J* = 9.91 Hz, 1.65 Hz), 3.67-3.80 (3H, m), 4.50 (1H, d, *J* = 1.65 Hz), 4.91 (1H, d, *J* = 9.91 Hz), 7.32 (2H, d, *J* = 8.26 Hz), 7.82 (2H, d, *J* = 8.26 Hz). ^{13}C NMR (75 MHz, $CDCl_3$) δ 21.5, 21.6, 22.1, 24.7, 24.8, 25.6, 26.4, 35.8, 37.4, 44.0, 46.4, 50.3, 66.5, 80.0, 125.9 (2C), 129.7 (2C), 141.6, 142.8, 171.6. MS (ESI$^+$) *m/z* (%): 426/428 [M + H$^+$, 100]. HRMS (ESI) calcd for $C_{21}H_{32}ClN_3O_2S$: 426.1977 MH$^+$; found, 426.1972.

Synthesis of (4′*S*,5′*S*)-*N*-[5-(2-chloro-2-propyl)-2,2-diphenylimidazolidin-4-yl]carbonyl-piperidine (12b). To a solution of (S_S,2*S*,3*S*)-*N*-[4-chloro-2-(diphenylmethyleneamino)-4-

methyl-3-(*p*-toluenesulfinylamino)pentanoyl]piperidine (*syn*-**5b**, 0.40 g, 0.73 mmol) in EtOH (20 mL) was added dropwise trifluoroacetic acid (10 equiv, 7.26 mmol, 0.56 mL) at rt. The reaction mixture was stirred for 16 h at rt and subsequently quenched with NH_4OH in H_2O until pH 10 and concentrated in vacuo. The residue was redissolved in water (10 mL) and NH_4OH in H_2O was added until pH 10. The aqueous phase was extracted with CH_2Cl_2 (3 × 10 mL). The combined organic phases were dried ($MgSO_4$), filtered and evaporated in vacuo. The crude product was purified by crystallization in diethyl ether to yield 0.16 g (0.40 mmol, 55%) of pure (4′*S*,5′*S*)-*N*-[5-(2-chloro-2-propyl)-2,2-diphenyl-imidazolidin-4-yl]carbonylpiperidine (**12b**).

(4′*S*,5′*S*)-*N*-[5-(2-Chloro-2-propyl)-2,2-diphenylimidazolidin-4-yl]carbonylpiperidine (12b). White crystals, yield 55% (0.16 g). $[\alpha]_D$ −33.4 (*c* 0.8, $CHCl_3$). Mp 132.7 ± 2.0 °C. IR (cm^{-1}): ν_{max} 707, 752, 1025, 1216, 1260, 1452, 1642, 3315 (weak). ^{1}H NMR (300 MHz, $CDCl_3$) δ 1.49-1.89 (6H, m), 1.52 (3H, s), 1.73 (3H, s), 2.59-2.74 (2H, m), 3.32-3.43 (1H, m), 3.52-3.69 (2H, m), 3.76-3.98 (2H, m), 3.85 (1H, d, *J* = 7.43 Hz), 7.13-7.35 (6H, m), 7.57-7.65 (4H, m). ^{13}C NMR (75 MHz, $CDCl_3$) δ 24.7, 25.5, 26.4, 31.3, 31.7, 43.5, 47.1, 60.4, 71.3, 73.3, 85.6, 125.7 (2C), 126.0 (2C), 127.1 (2C), 128.2 (2C), 128.4 (2C), 146.2, 146.7, 169.6. MS (ESI^+) *m/z* (%): 412/414 [M + H^+, 100]. HRMS (ESI) calcd for $C_{24}H_{30}ClN_3O$: 412.2150 MH^+; found, 376.2376 (100%), 412.2144 (70%).

Synthesis of (2*S*,3*S*)-*N*-[2,3-diamino-4-chloro-4-methylpentanoyl]pyrrolidine dihydro-chloride (13a). (S_S,2*S*,3*S*)-*N*-[4-Chloro-2-(diphenylmethyleneamino)-4-methyl-3-(*p*-toluenesulfinylamino)-pentanoyl]pyrrolidine (*syn*-**5a**, 0.19 g, 0.35 mmol) was dissolved in a mixture of 0.5 M (aq.) HCl/EtOAc (2:1) (12 mL) and the mixture was stirred for 30 min at rt. The organic phase was removed and the aqueous phase was washed with EtOAc (3 × 5 mL).

Subsequently, the aqueous phase was lyophilized to yield 0.09 g (0.29 mmol, 83%) of pure (2*S*,3*S*)-*N*-[2,3-diamino-4-chloro-4-methylpentanoyl]pyrrolidine dihydrochloride (**13a**).

(2*S*,3*S*)-*N*-[2,3-diamino-4-chloro-4-methylpentanoyl]pyrrolidine dihydrochloride (13a). White crystals, yield 83% (0.09 g). $[\alpha]_D$ −3.1 (*c* 2.4, MeOH). Mp 227.3 ± 2.0 °C. IR (cm^{-1}): ν_{max} 1123, 1166, 1404, 1453, 1514, 1543, 1636, 2973, 3399. ^1H NMR (300 MHz, D_2O) δ 1.79 (3H, s), 1.82-2.04 (4H, m), 1.86 (3H, s), 3.35-3.60 (3H, m), 3.62-3.77 (1H, m), 4.07 (1H, d, *J* = 2.20 Hz), 4.90 (1H, d, *J* = 2.20 Hz). ^{13}C NMR (75 MHz, D_2O) δ 23.8, 25.5, 29.0, 30.9, 47.3, 47.6, 49.8, 57.6, 67.8, 164.1. MS (ESI^+) *m/z* (%): 234/236 [M + H^+ – 2 × HCl, 100]. HRMS (ESI) calcd for $C_{10}H_{20}ClN_3O$: 198.1601 [MH^+ − 3×HCl]; found, 198.1596.

II. Copies of ^{1}H NMR and ^{13}C NMR spectra of 3, *syn*-5, 8 and 10–13

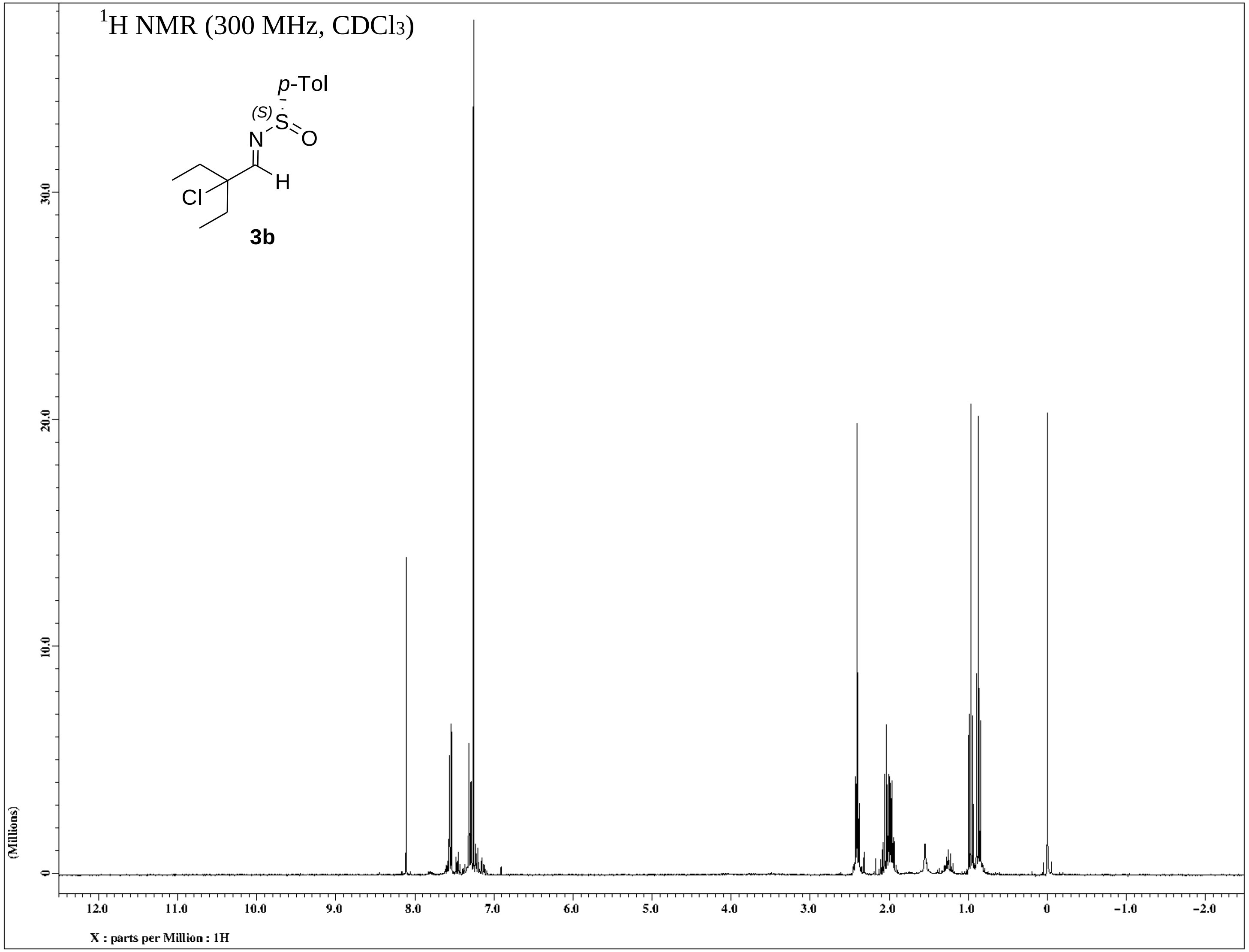

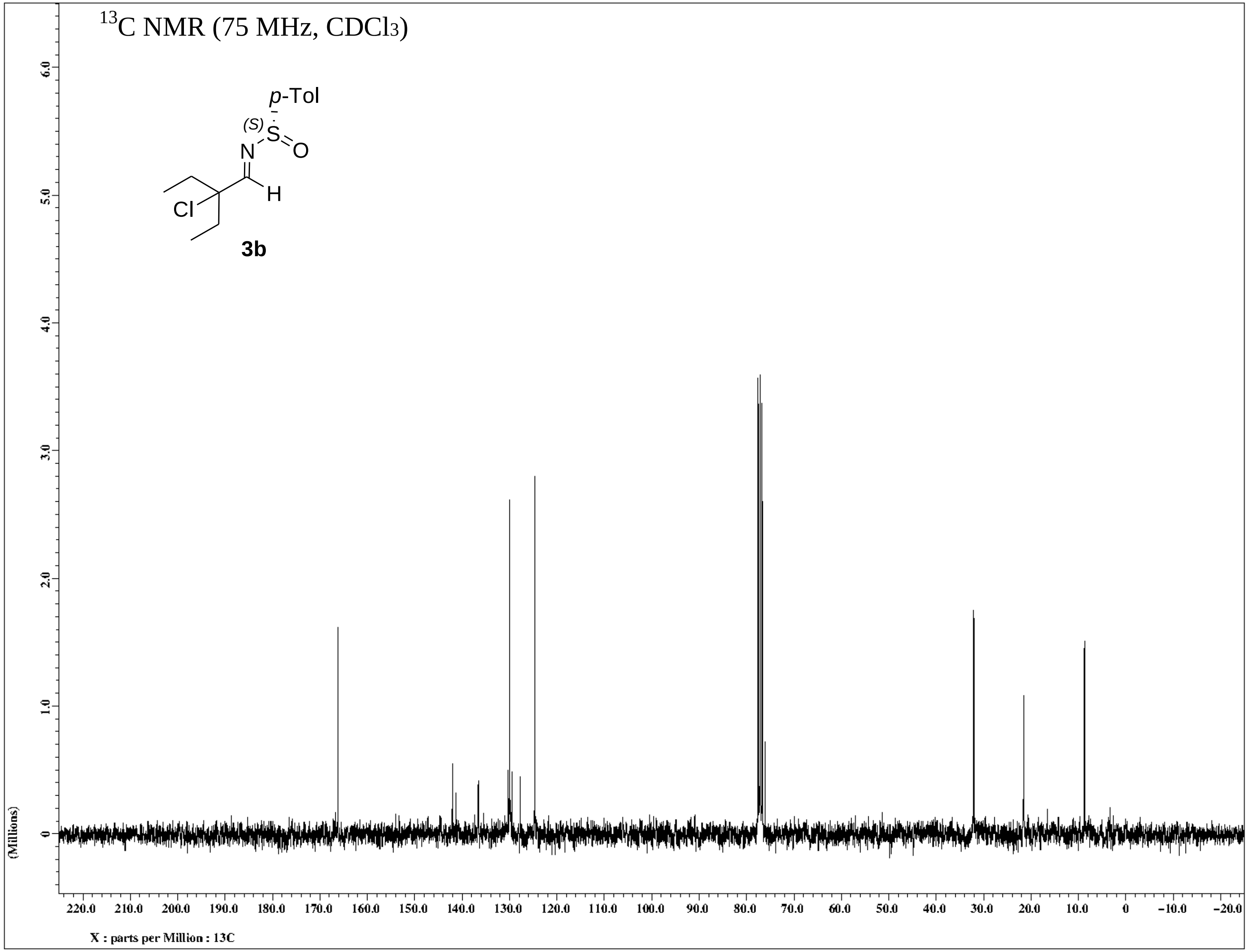

^{13}C NMR (75 MHz, CDCl$_3$)
p-Tol
(S)
S
O
N
H
Cl
3b
X : parts per Million : 13C
(Millions)

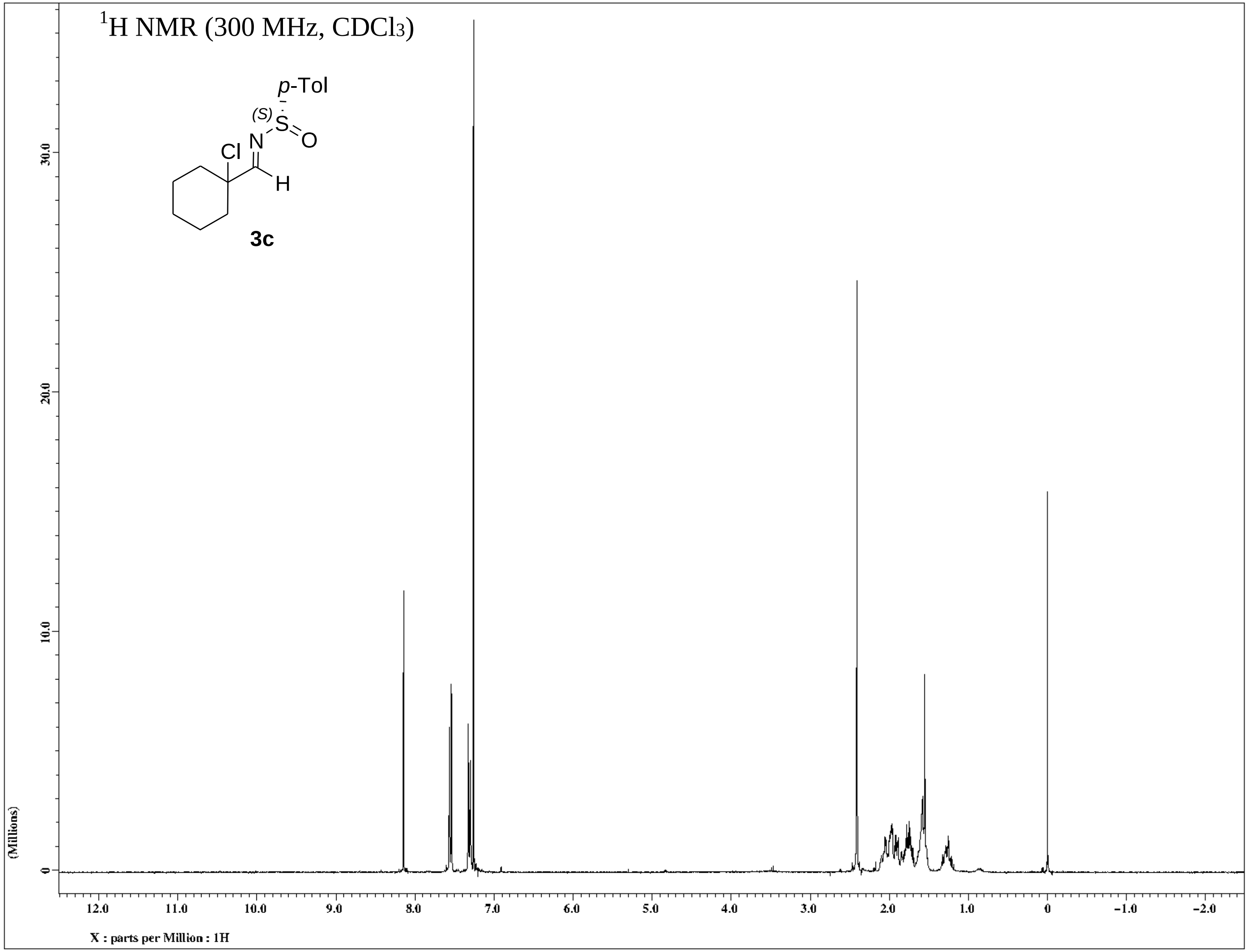

1H NMR (300 MHz, CDCl3)
p-Tol
(S)
S
O
N
Cl
H
3c
(Millions)
0
10.0
20.0
30.0
12.0
11.0
10.0
9.0
8.0
7.0
6.0
5.0
4.0
3.0
2.0
1.0
0
−1.0
−2.0
X : parts per Million : 1H

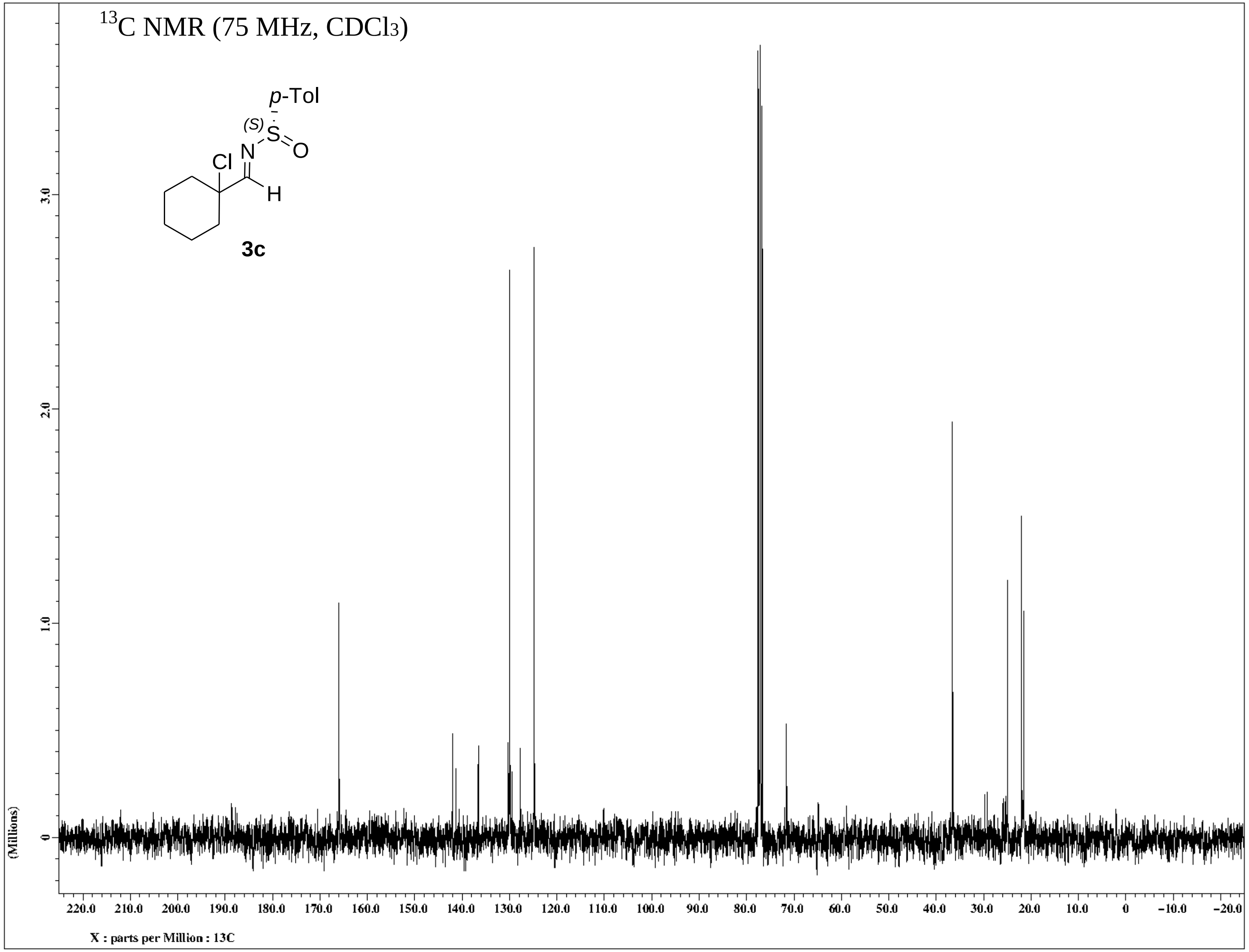

^{13}C NMR (75 MHz, CDCl$_3$)
p-Tol
(S)
S
O
N
Cl
H
3c
3.0
2.0
1.0
0
(Millions)
220.0 210.0 200.0 190.0 180.0 170.0 160.0 150.0 140.0 130.0 120.0 110.0 100.0 90.0 80.0 70.0 60.0 50.0 40.0 30.0 20.0 10.0 0 −10.0 −20.0
X : parts per Million : 13C

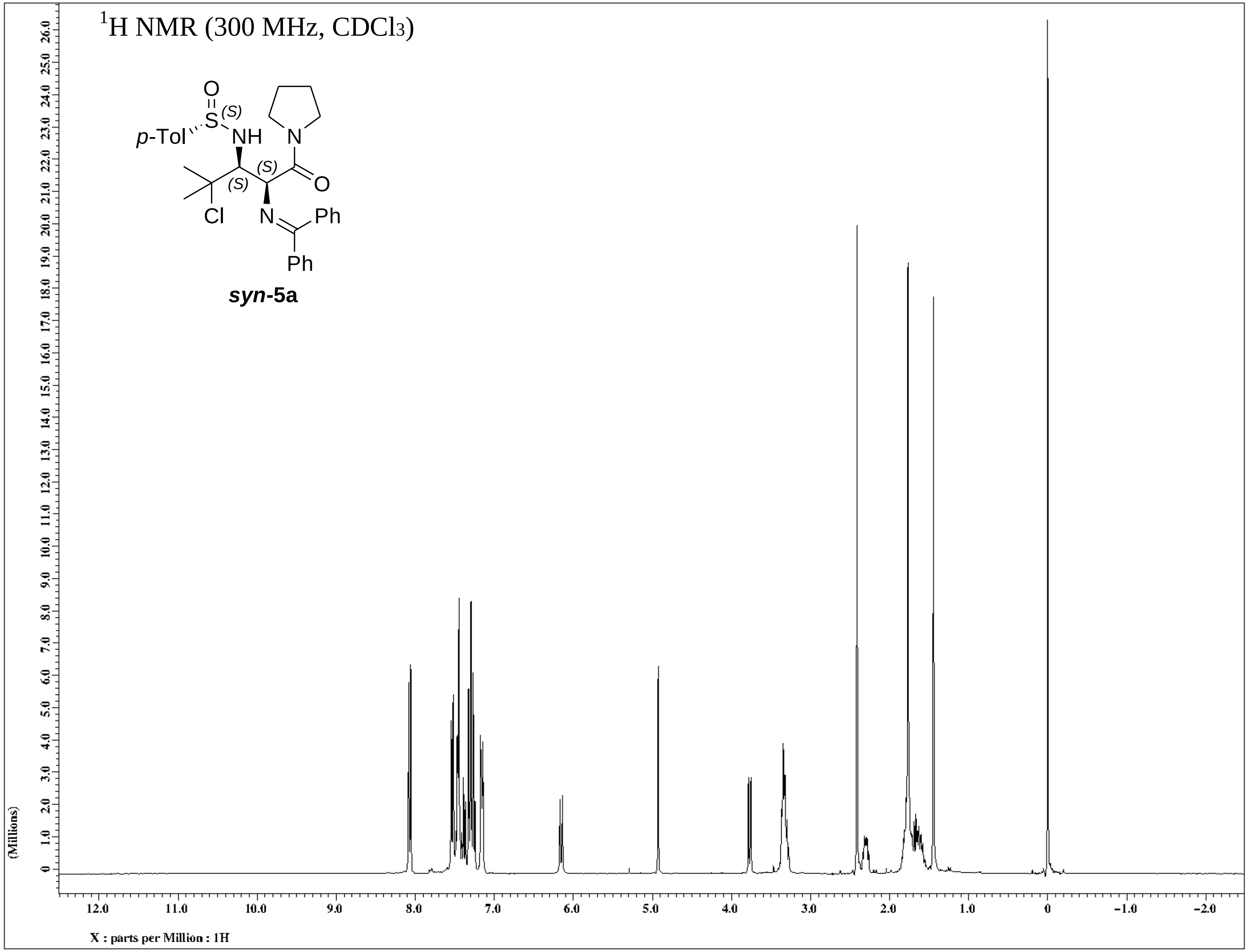

¹H NMR (300 MHz, CDCl3)
O
(S)
p-Tol
S
NH
N
(S)
(S)
O
Cl
N
Ph
Ph
syn-5a
(Millions)
X : parts per Million : 1H

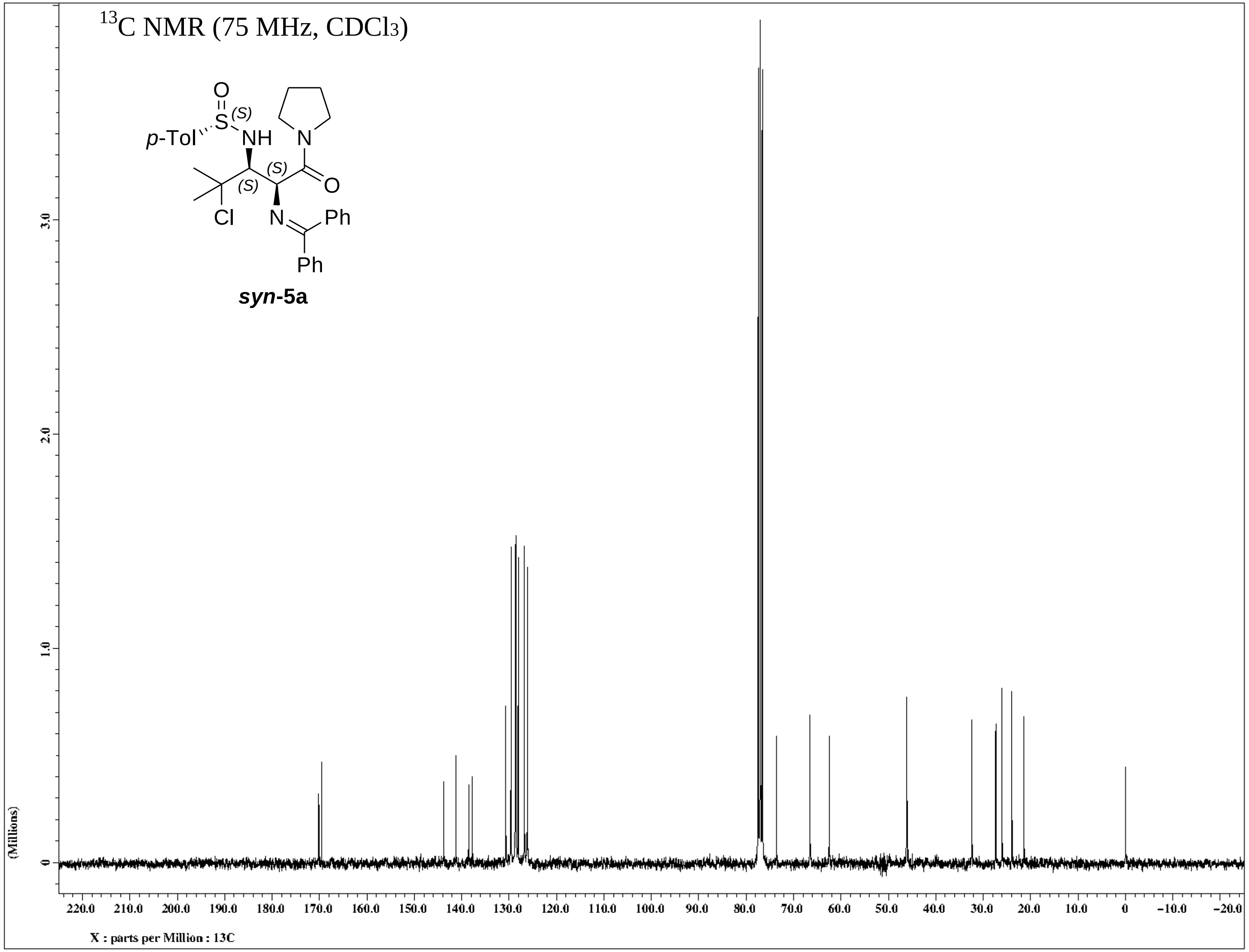

^{13}C NMR (75 MHz, CDCl$_3$)
p-Tol
O
S
(S)
NH
N
(S)
(S)
O
Cl
N
Ph
Ph
syn-5a
(Millions)
X : parts per Million : 13C

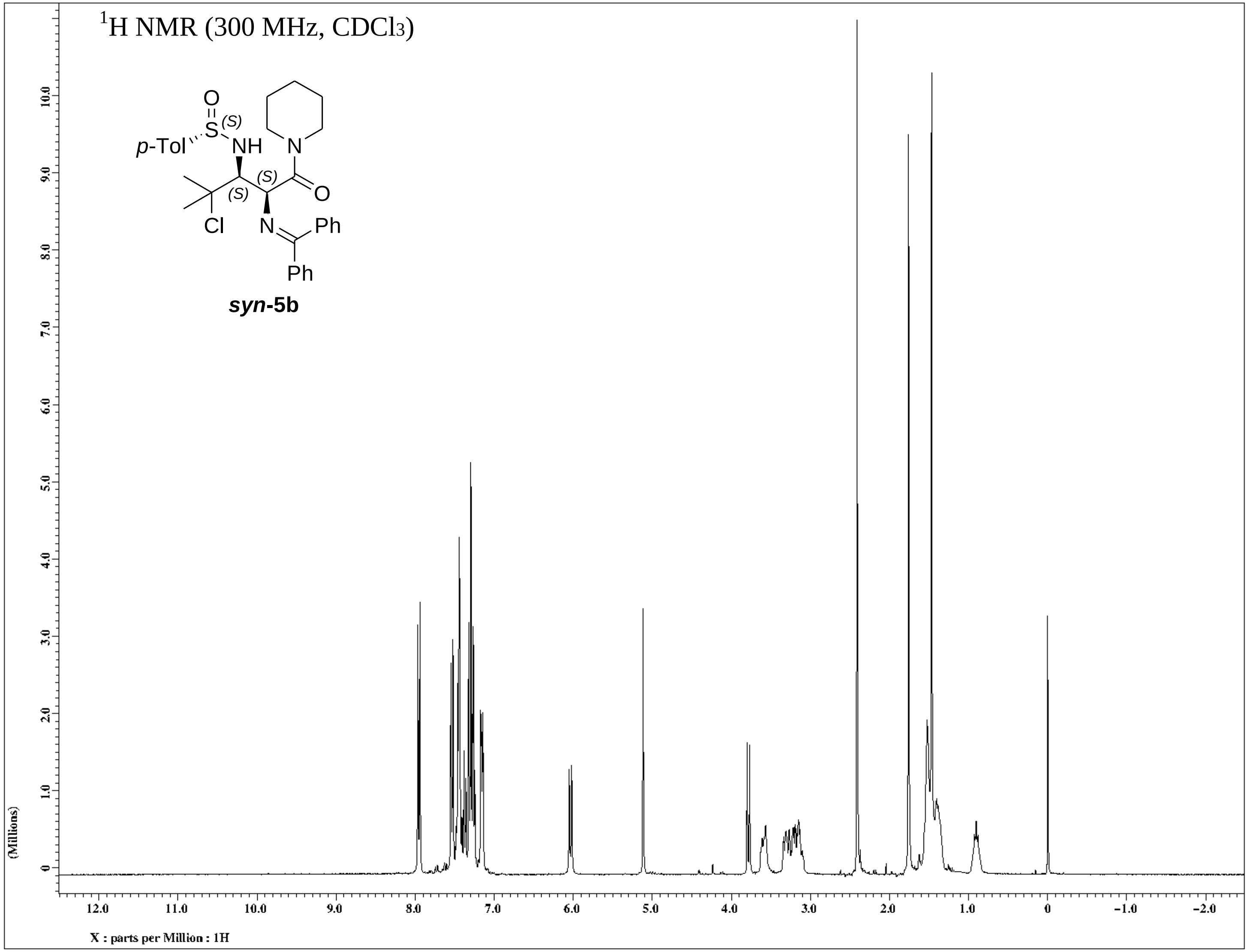

1H NMR (300 MHz, CDCl3)
O
(S)
S
p-Tol
NH
N
(S)
(S)
O
Cl
N
Ph
Ph
syn-5b
(Millions)
X : parts per Million : 1H

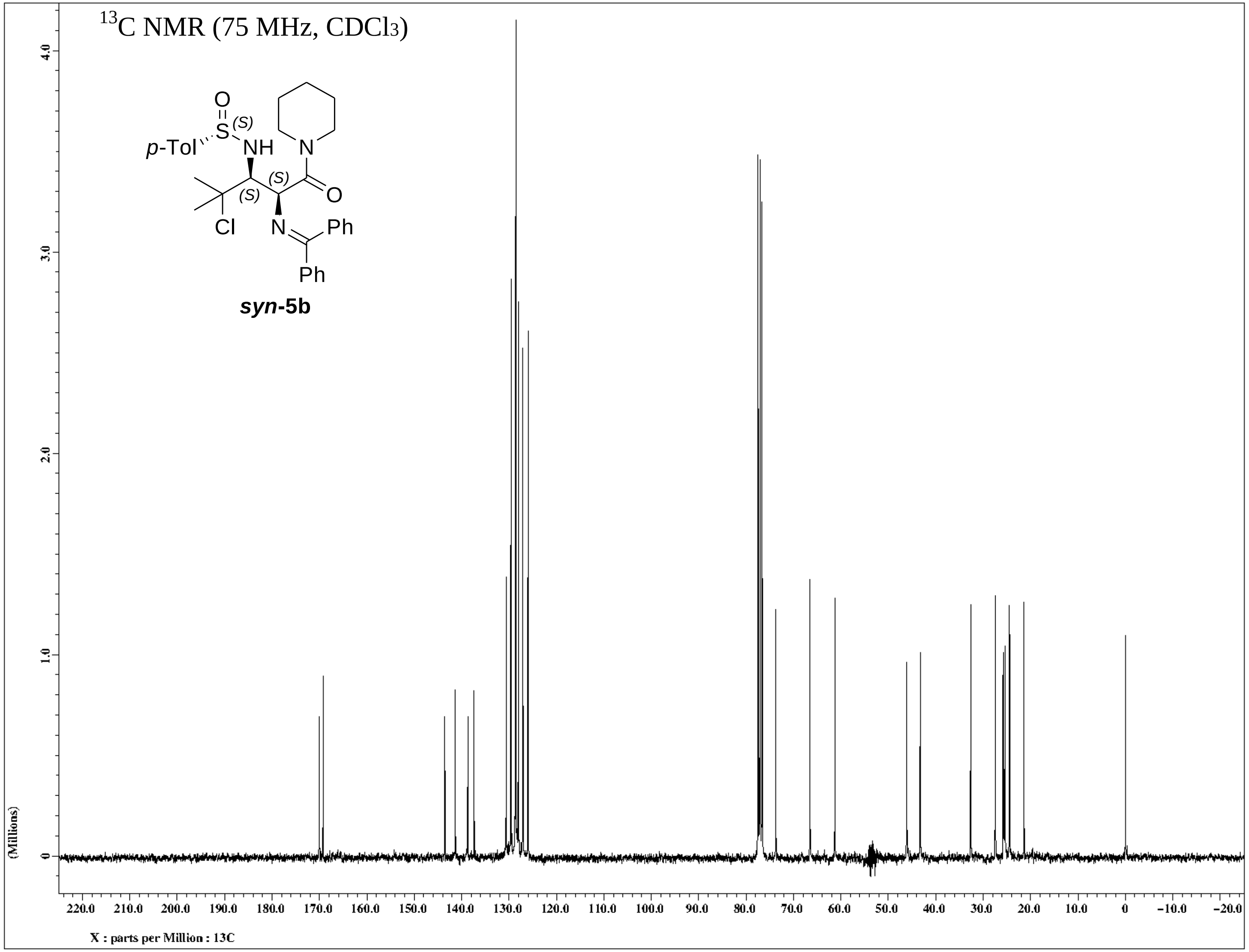

13C NMR (75 MHz, CDCl3)
O
(S)
p-Tol
S
NH
N
(S)
(S)
O
Cl
N
Ph
Ph
syn-5b
4.0
3.0
2.0
1.0
0
(Millions)
220.0 210.0 200.0 190.0 180.0 170.0 160.0 150.0 140.0 130.0 120.0 110.0 100.0 90.0 80.0 70.0 60.0 50.0 40.0 30.0 20.0 10.0 0 -10.0 -20.0
X : parts per Million : 13C

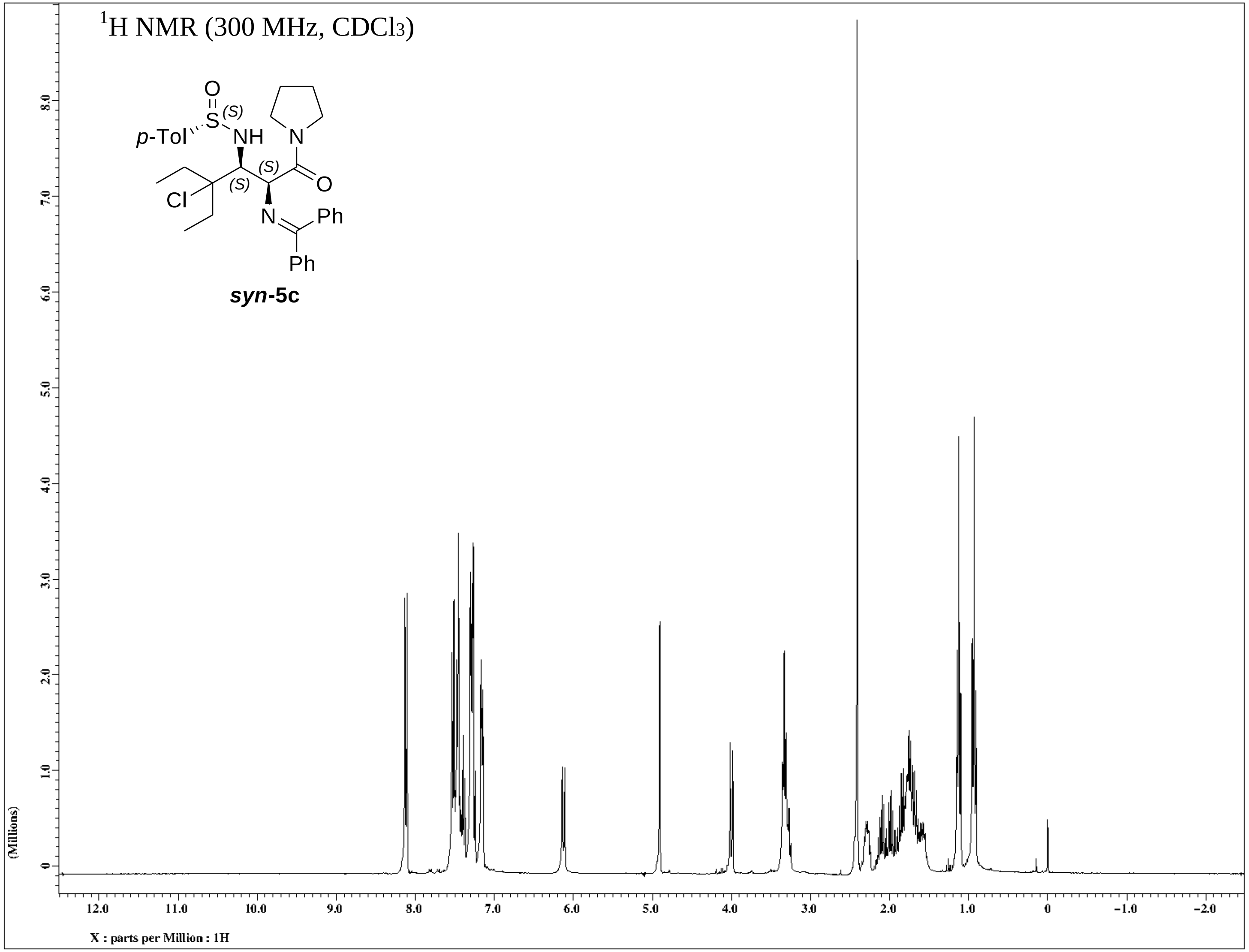

^{1}H NMR (300 MHz, CDCl3)
O
(S)
p-Tol
S
NH
N
(S)
(S)
O
Cl
N
Ph
Ph
syn-5c
(Millions)
X : parts per Million : 1H

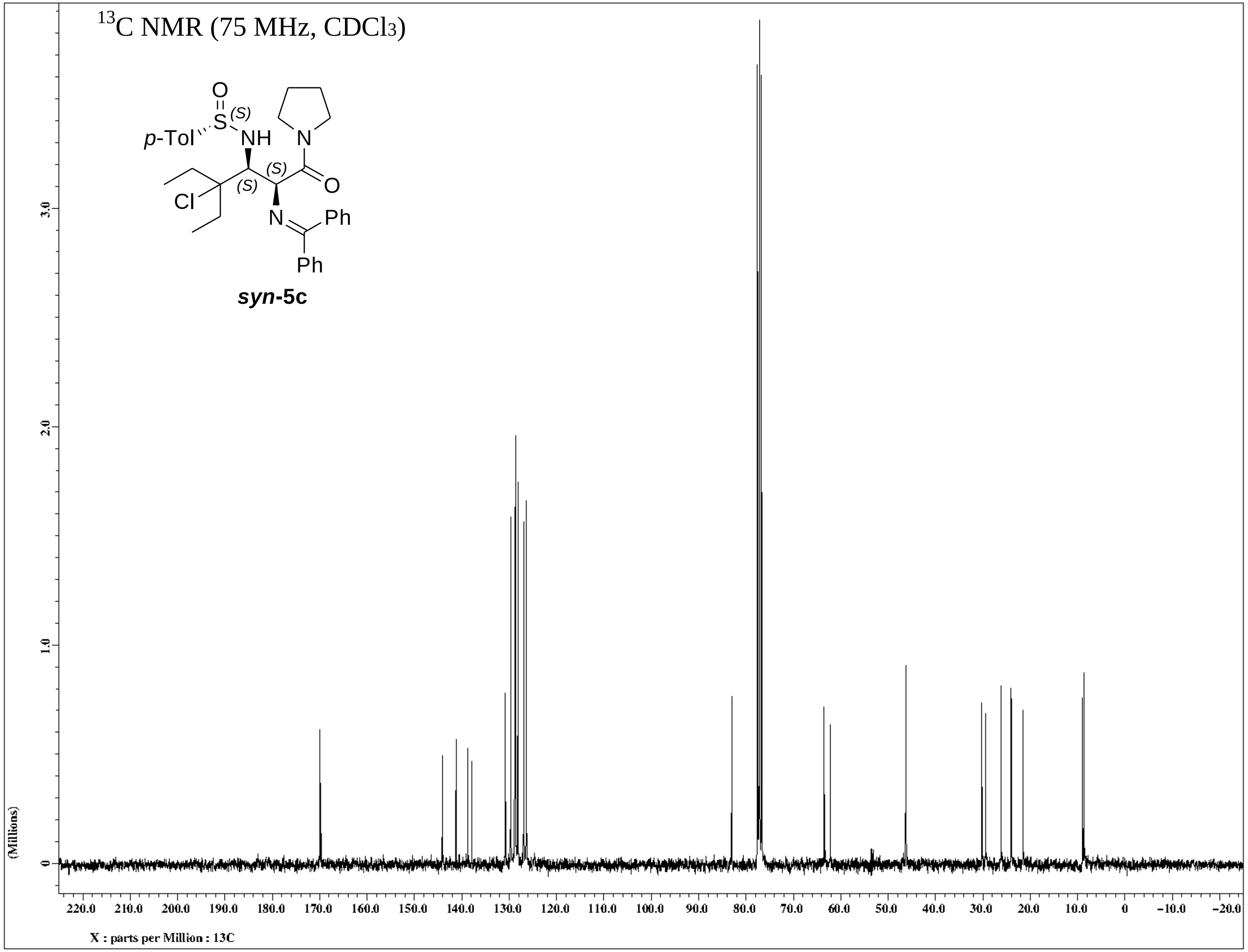

13C NMR (75 MHz, CDCl3)
p-Tol
O
S
(S)
NH
N
(S)
(S)
O
Cl
N
Ph
Ph
syn-5c
(Millions)
0
1.0
2.0
3.0
220.0 210.0 200.0 190.0 180.0 170.0 160.0 150.0 140.0 130.0 120.0 110.0 100.0 90.0 80.0 70.0 60.0 50.0 40.0 30.0 20.0 10.0 0 −10.0 −20.0
X : parts per Million : 13C

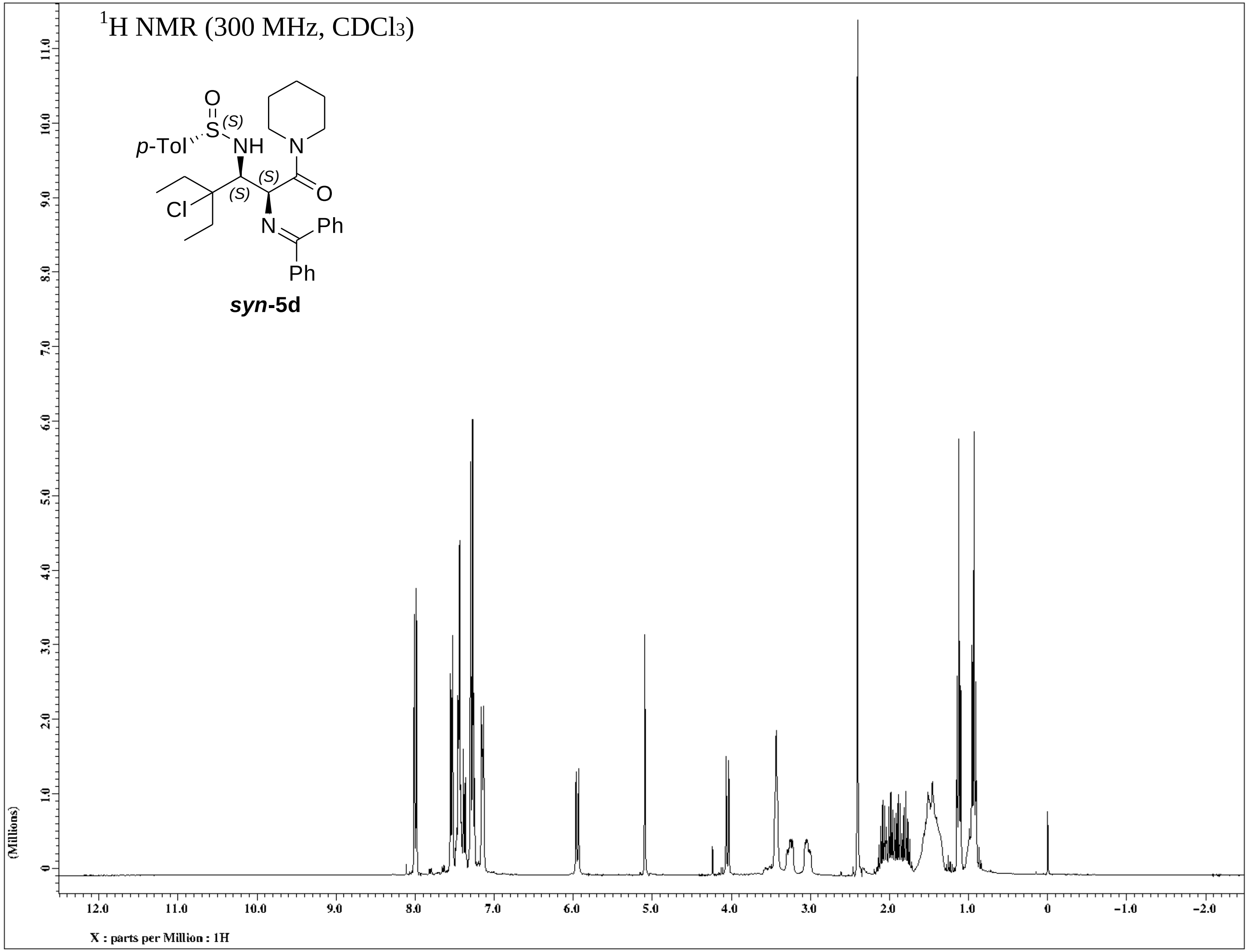

^{1}H NMR (300 MHz, CDCl3)
p-Tol
(S)
NH
N
O
(S)
(S)
Cl
N
Ph
Ph
syn-5d
(Millions)
X : parts per Million : 1H

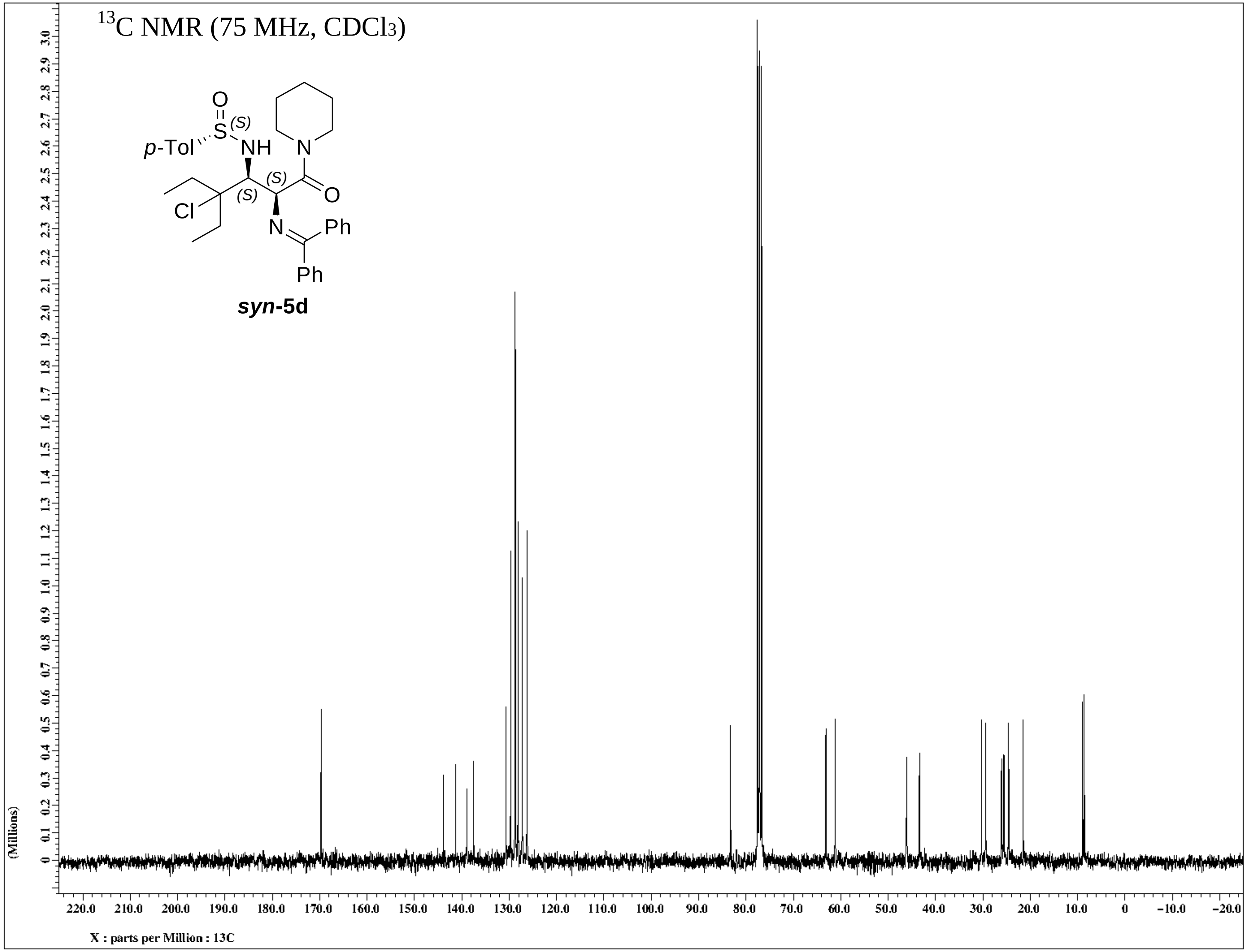

^{13}C NMR (75 MHz, CDCl3)
O
S
(S)
p-Tol
NH
N
(S)
(S)
O
Cl
N
Ph
Ph
syn-5d
(Millions)
X : parts per Million : 13C

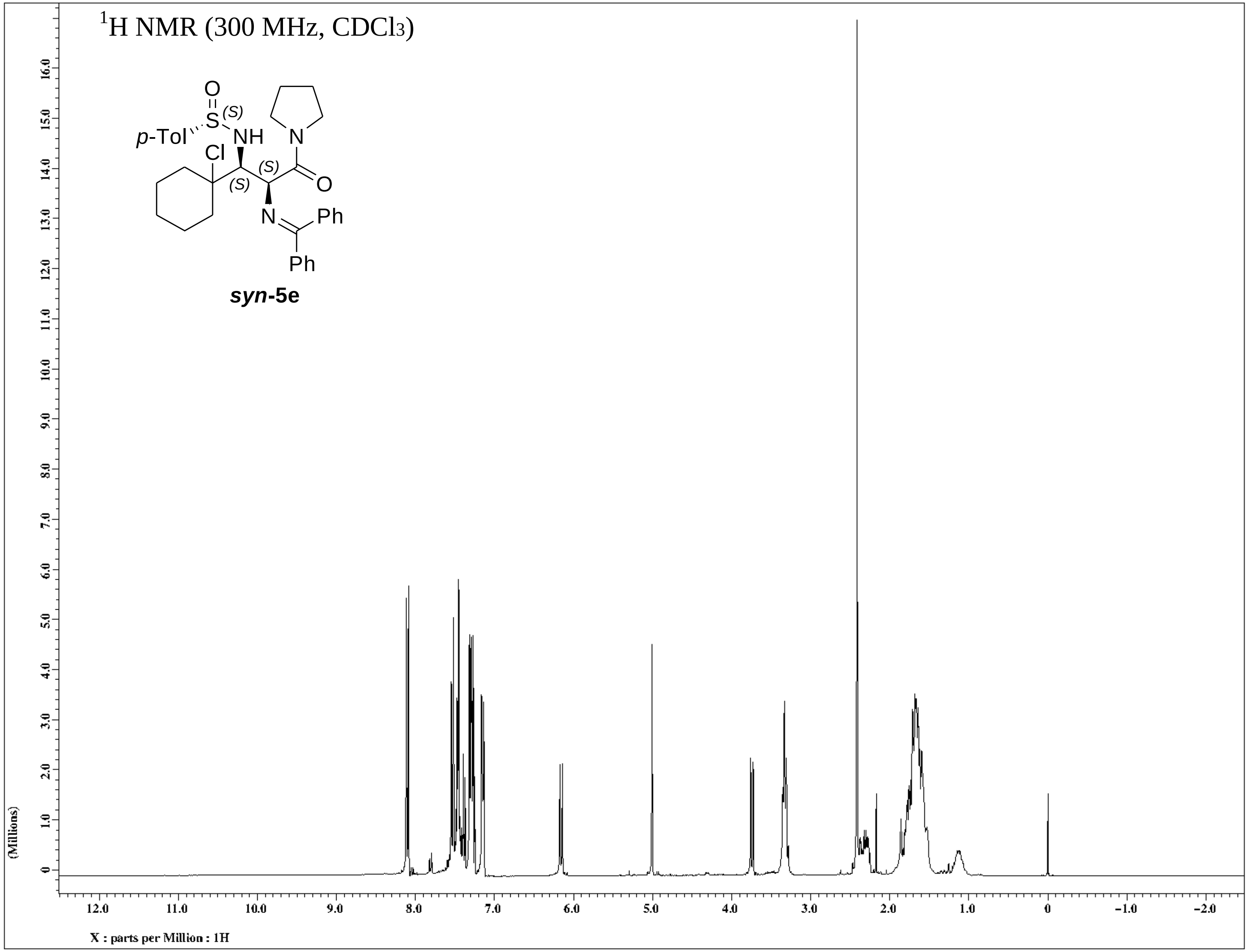

^{1}H NMR (300 MHz, CDCl3)
O
(S)
p-Tol
S
NH
N
Cl
(S)
(S)
O
N
Ph
Ph
syn-5e
(Millions)
16.0
15.0
14.0
13.0
12.0
11.0
10.0
9.0
8.0
7.0
6.0
5.0
4.0
3.0
2.0
1.0
0
12.0
11.0
10.0
9.0
8.0
7.0
6.0
5.0
4.0
3.0
2.0
1.0
0
−1.0
−2.0
X : parts per Million : 1H

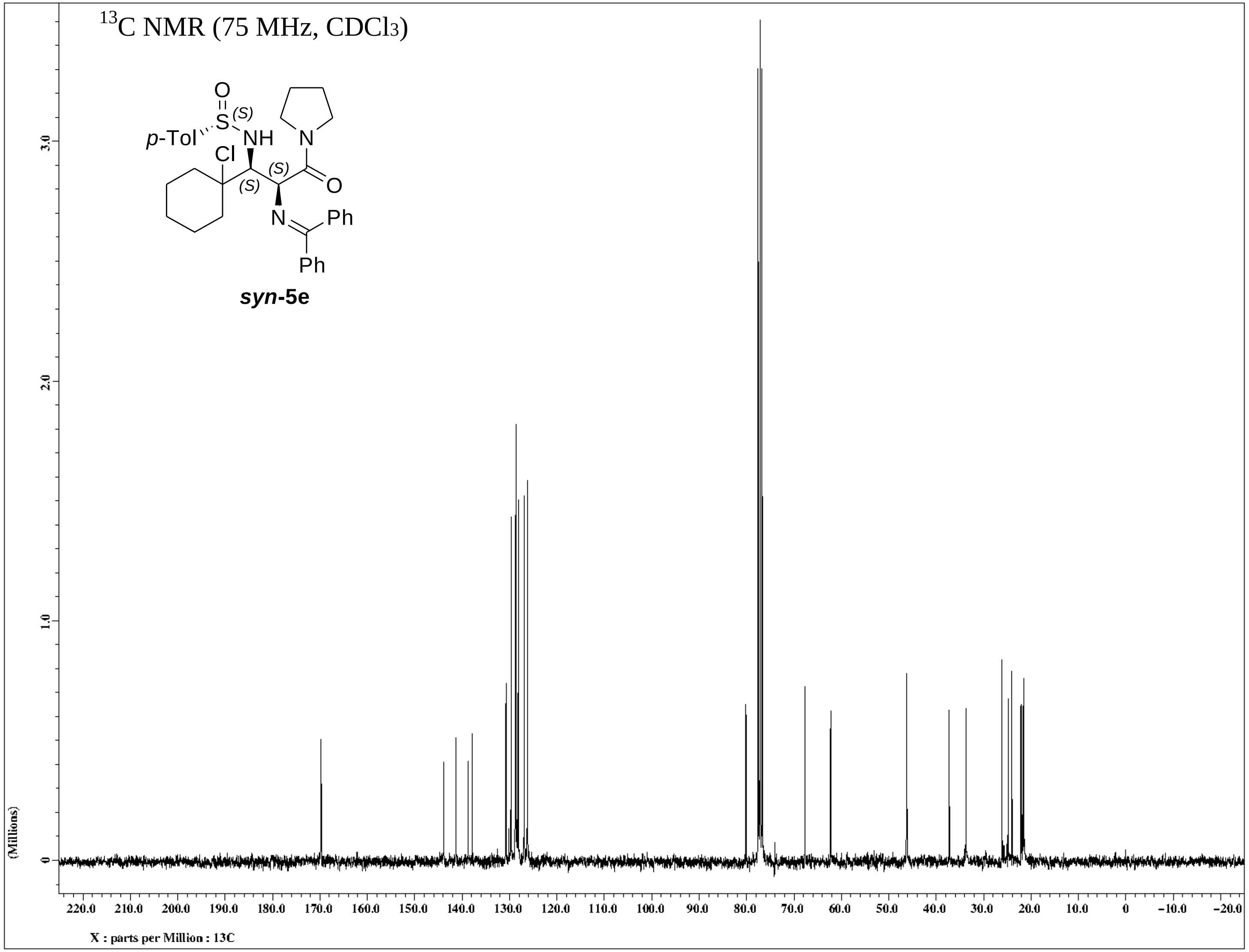

^{13}C NMR (75 MHz, CDCl$_3$)
O
(S)
p-Tol
S
NH
N
Cl
(S)
(S)
O
N
Ph
Ph
syn-5e
3.0
2.0
1.0
0
(Millions)
220.0 210.0 200.0 190.0 180.0 170.0 160.0 150.0 140.0 130.0 120.0 110.0 100.0 90.0 80.0 70.0 60.0 50.0 40.0 30.0 20.0 10.0 0 −10.0 −20.0
X : parts per Million : 13C

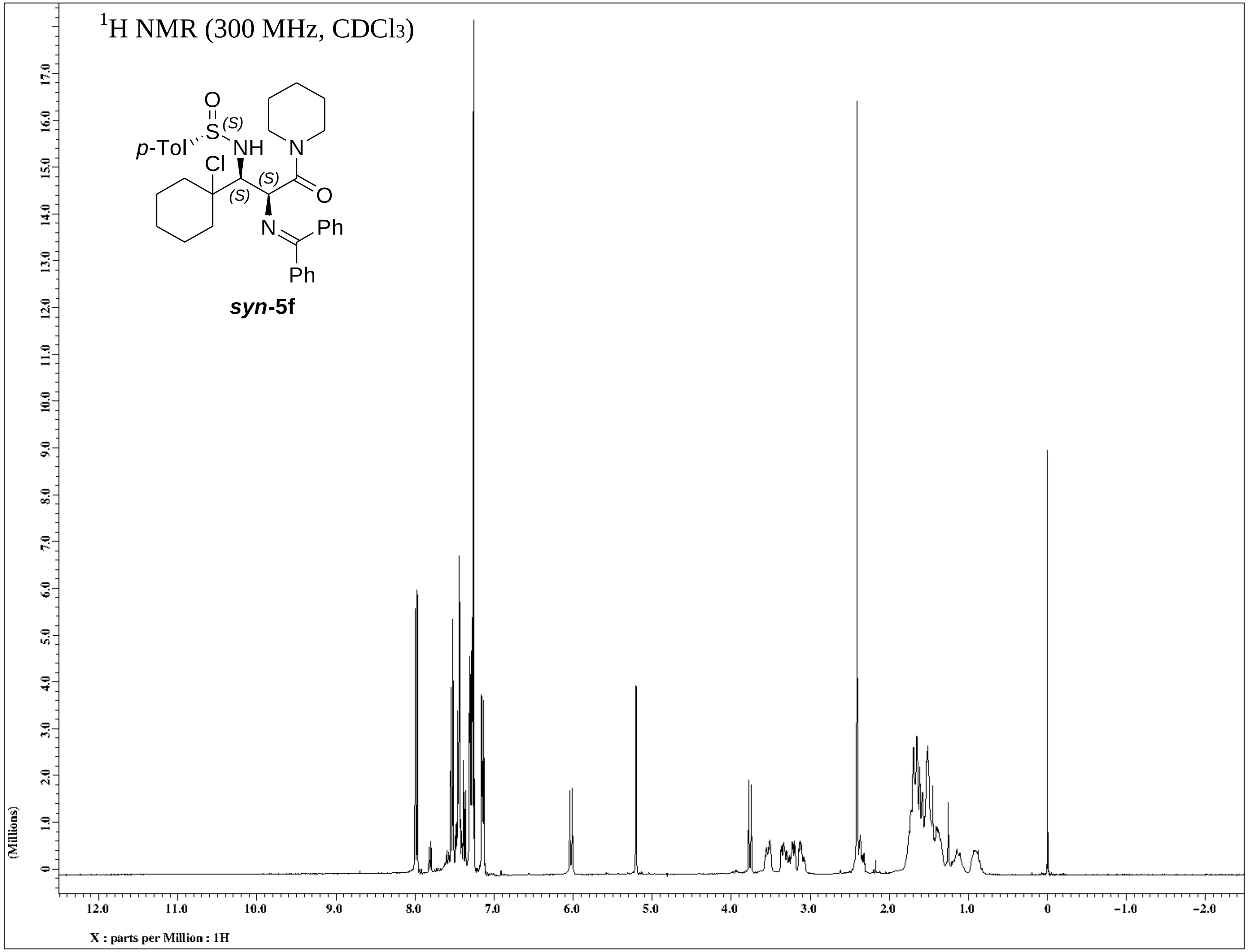

1H NMR (300 MHz, CDCl3)
O
S
(S)
p-Tol
NH
N
Cl
(S)
(S)
O
N
Ph
Ph
syn-5f
(Millions)
X : parts per Million : 1H

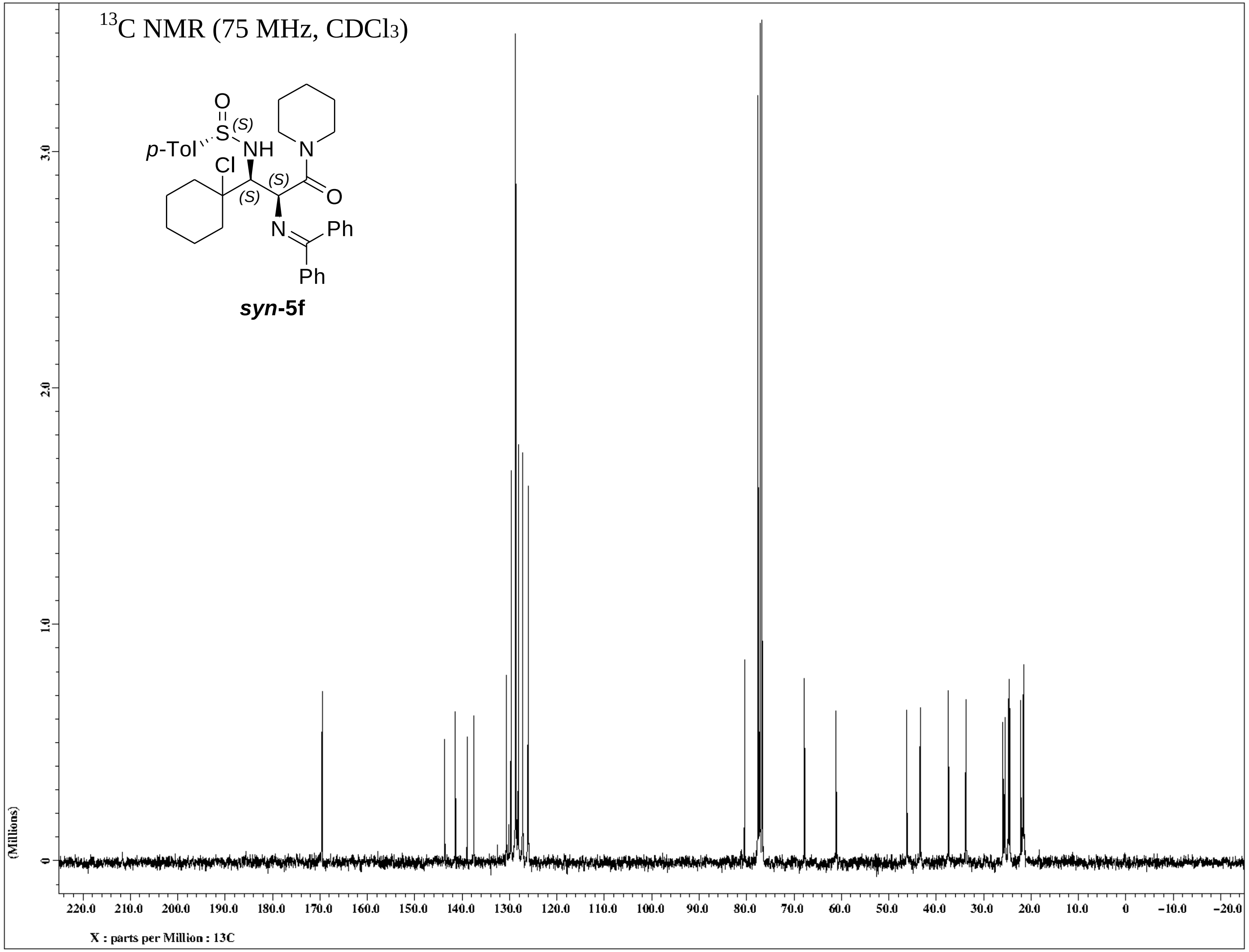

^{13}C NMR (75 MHz, CDCl3)
p-Tol
O
S
(S)
NH
Cl
(S)
(S)
N
O
N
Ph
Ph
syn-5f
(Millions)
3.0
2.0
1.0
0
220.0 210.0 200.0 190.0 180.0 170.0 160.0 150.0 140.0 130.0 120.0 110.0 100.0 90.0 80.0 70.0 60.0 50.0 40.0 30.0 20.0 10.0 0 −10.0 −20.0
X : parts per Million : 13C

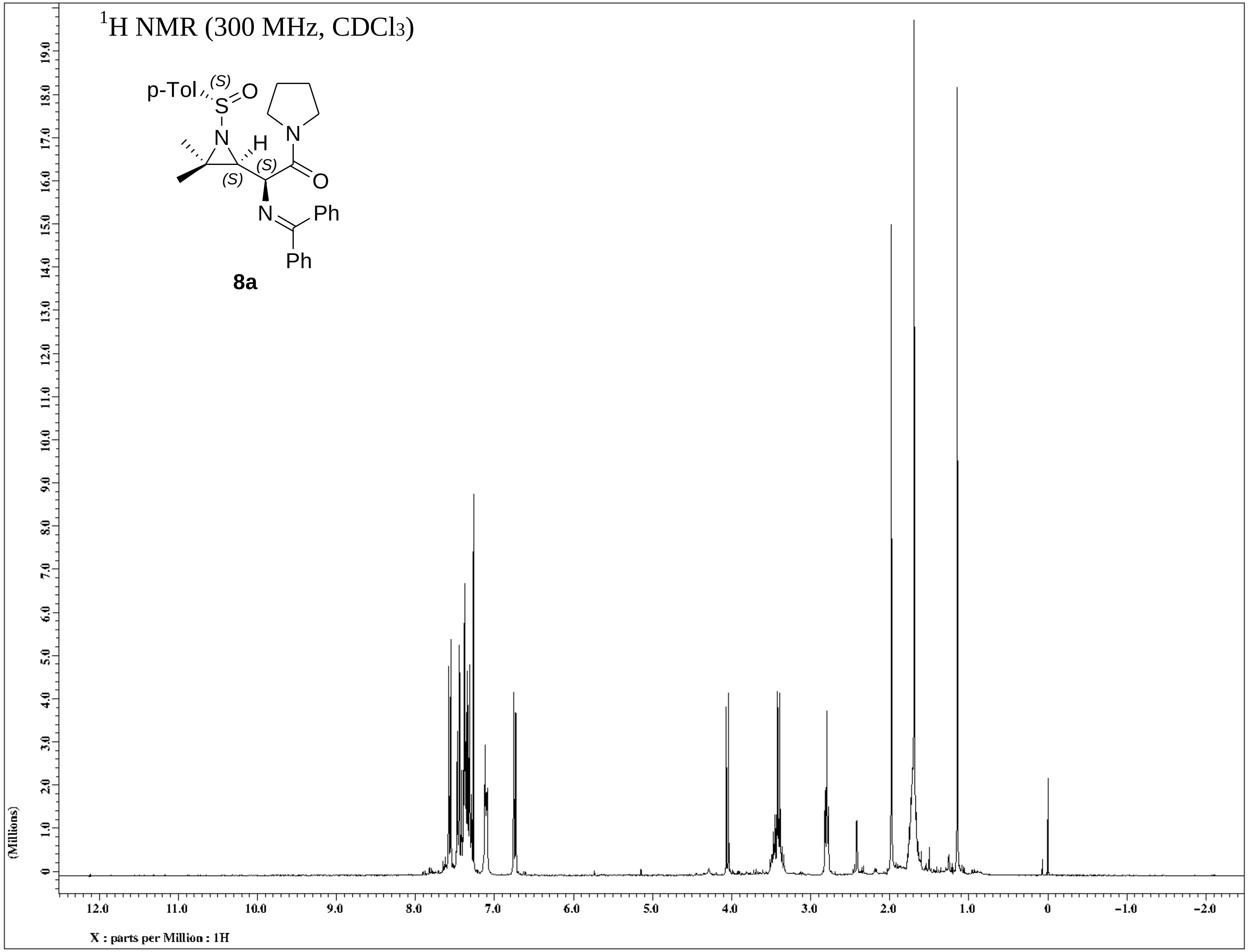

1H NMR (300 MHz, CDCl3)
p-Tol
(S)
S
O
N
H
(S)
(S)
O
N
N
Ph
Ph
8a
(Millions)
X : parts per Million : 1H

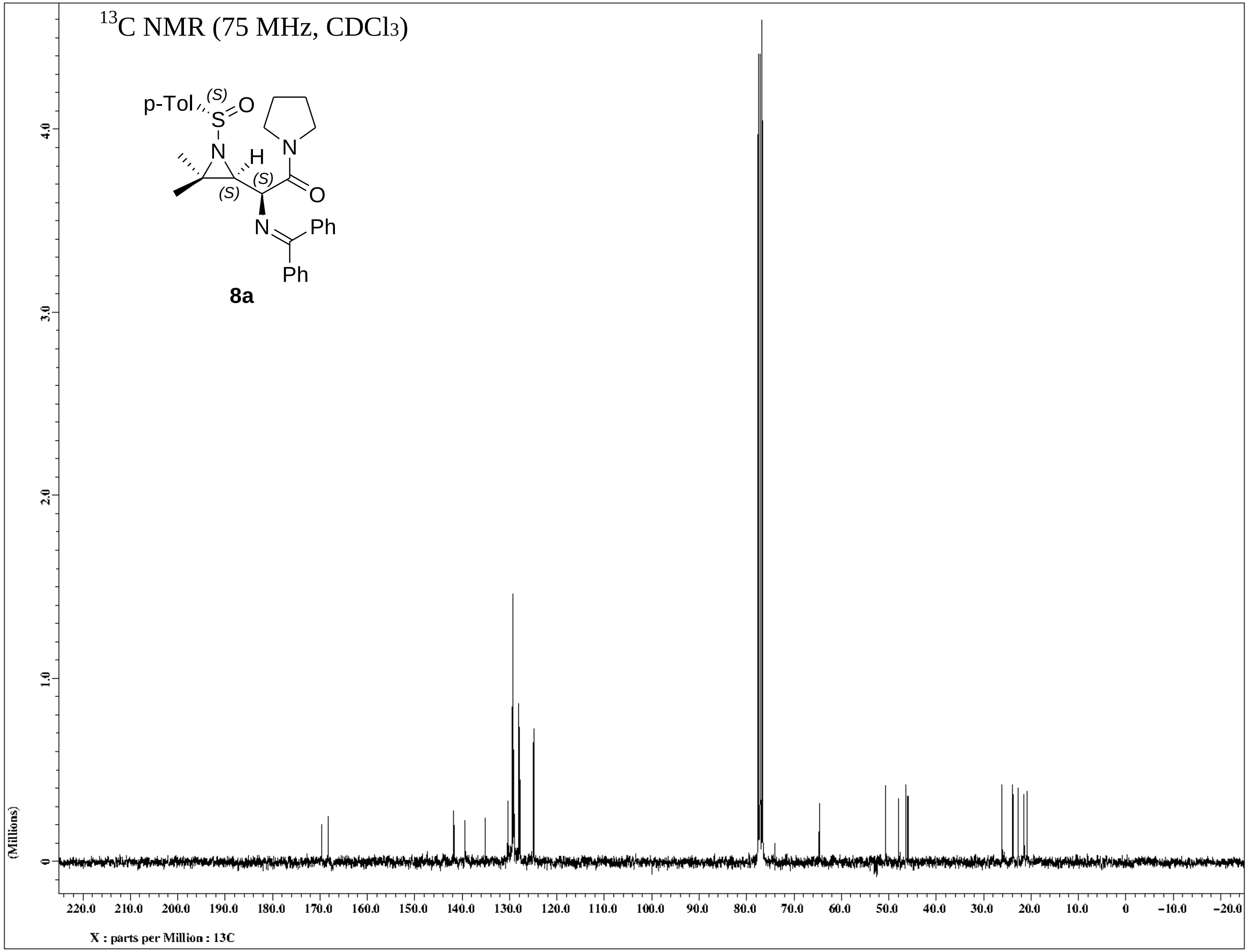

^{13}C NMR (75 MHz, CDCl3)
p-Tol
(S)
S
O
N
H
(S)
(S)
N
O
N
Ph
Ph
8a
(Millions)
4.0
3.0
2.0
1.0
0
220.0 210.0 200.0 190.0 180.0 170.0 160.0 150.0 140.0 130.0 120.0 110.0 100.0 90.0 80.0 70.0 60.0 50.0 40.0 30.0 20.0 10.0 0 -10.0 -20.0
X : parts per Million : 13C

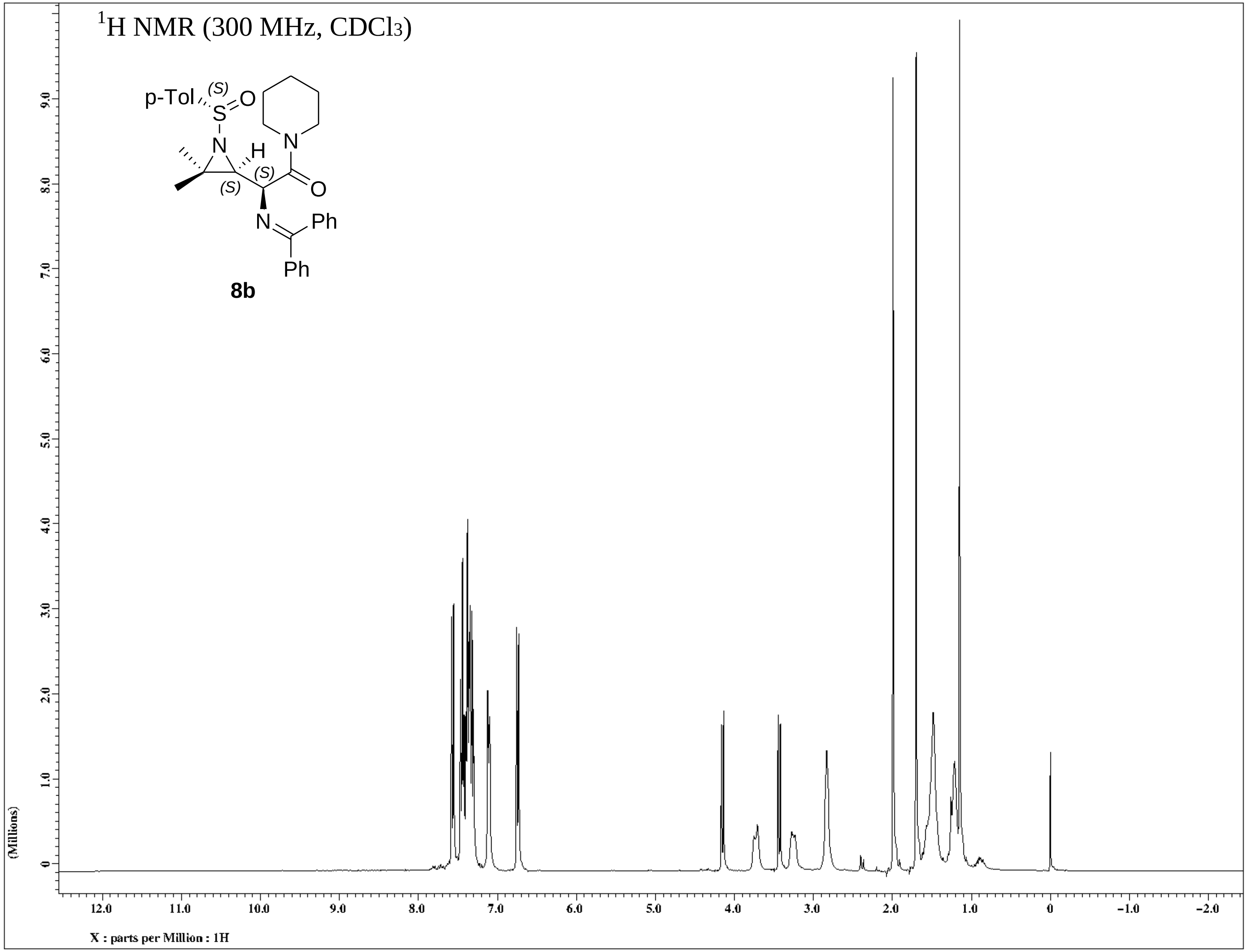

1H NMR (300 MHz, CDCl3)
p-Tol
(S)
S
O
N
H
(S)
(S)
N
O
N
Ph
Ph
8b
(Millions)
X : parts per Million : 1H

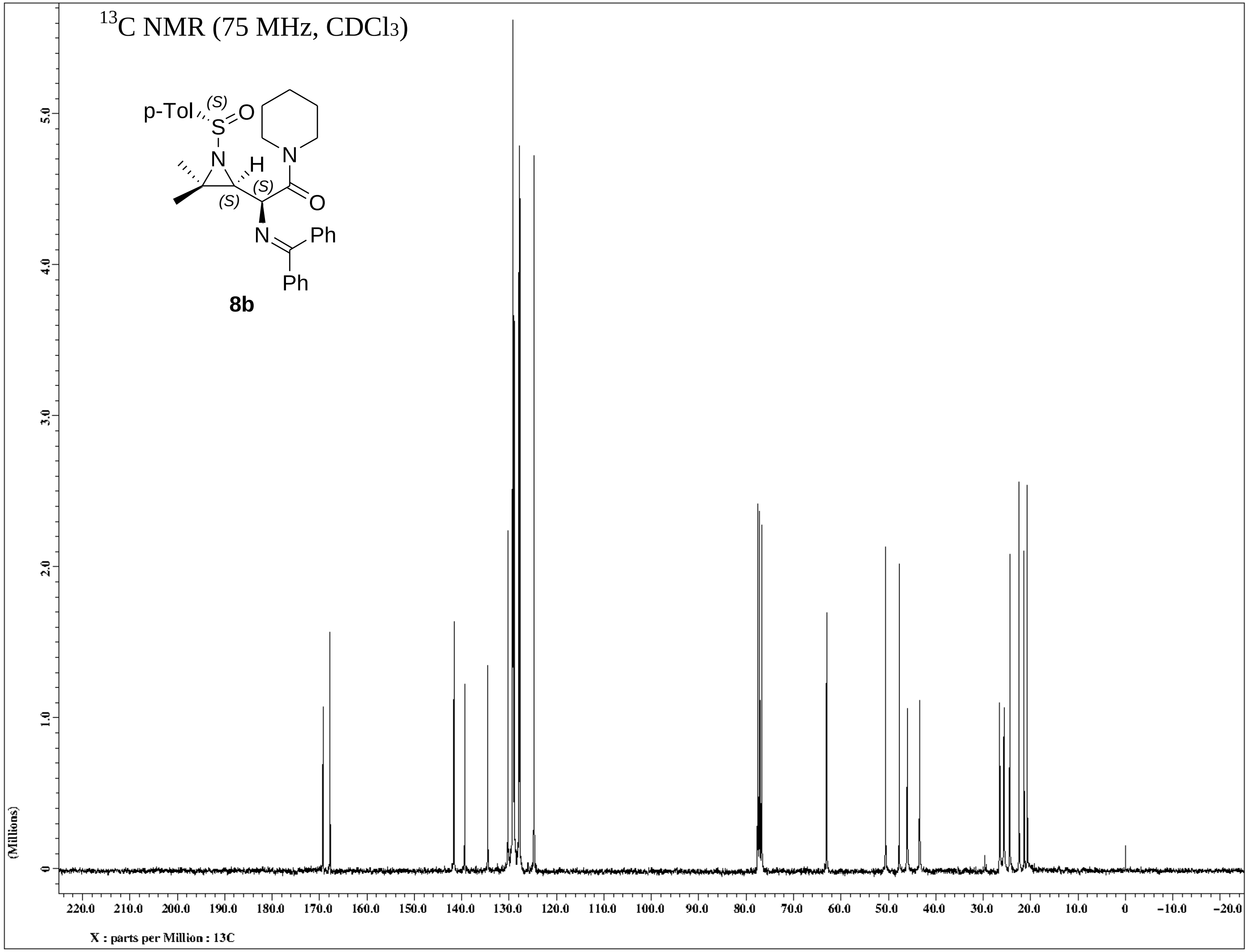

^{13}C NMR (75 MHz, CDCl$_3$)
p-Tol
(S)
S
O
N
H
(S)
(S)
O
N
N
Ph
Ph
8b
(Millions)
X : parts per Million : 13C

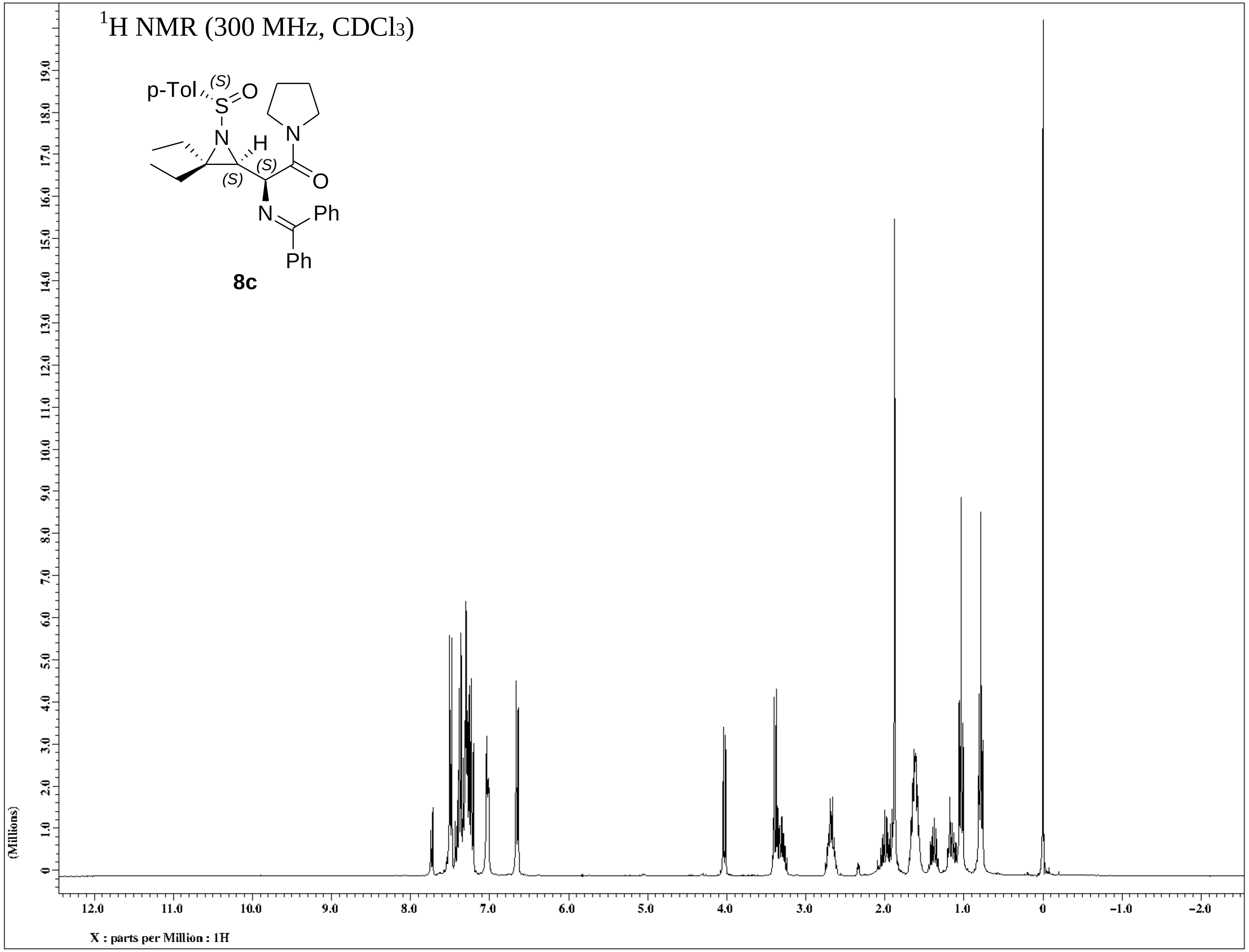

^{1}H NMR (300 MHz, CDCl3)
p-Tol
(S)
S
O
N
H
(S)
(S)
N
O
N
Ph
Ph
8c
(Millions)
X : parts per Million : 1H

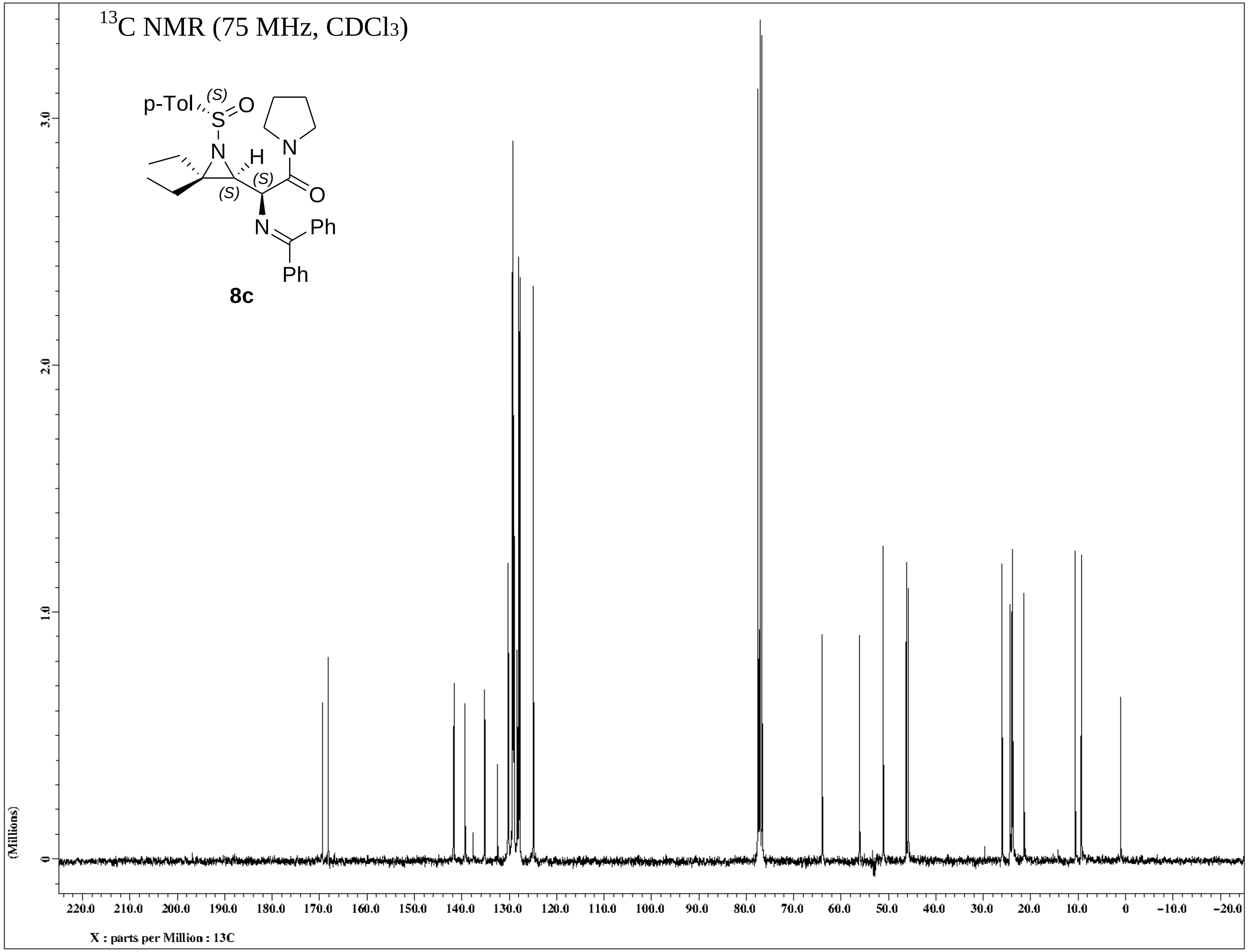

^{13}C NMR (75 MHz, CDCl$_3$)
p-Tol
(S)
S
O
N
H
(S)
(S)
N
O
N
Ph
Ph
8c
3.0
2.0
1.0
0
(Millions)
220.0
210.0
200.0
190.0
180.0
170.0
160.0
150.0
140.0
130.0
120.0
110.0
100.0
90.0
80.0
70.0
60.0
50.0
40.0
30.0
20.0
10.0
0
-10.0
-20.0
X : parts per Million : 13C

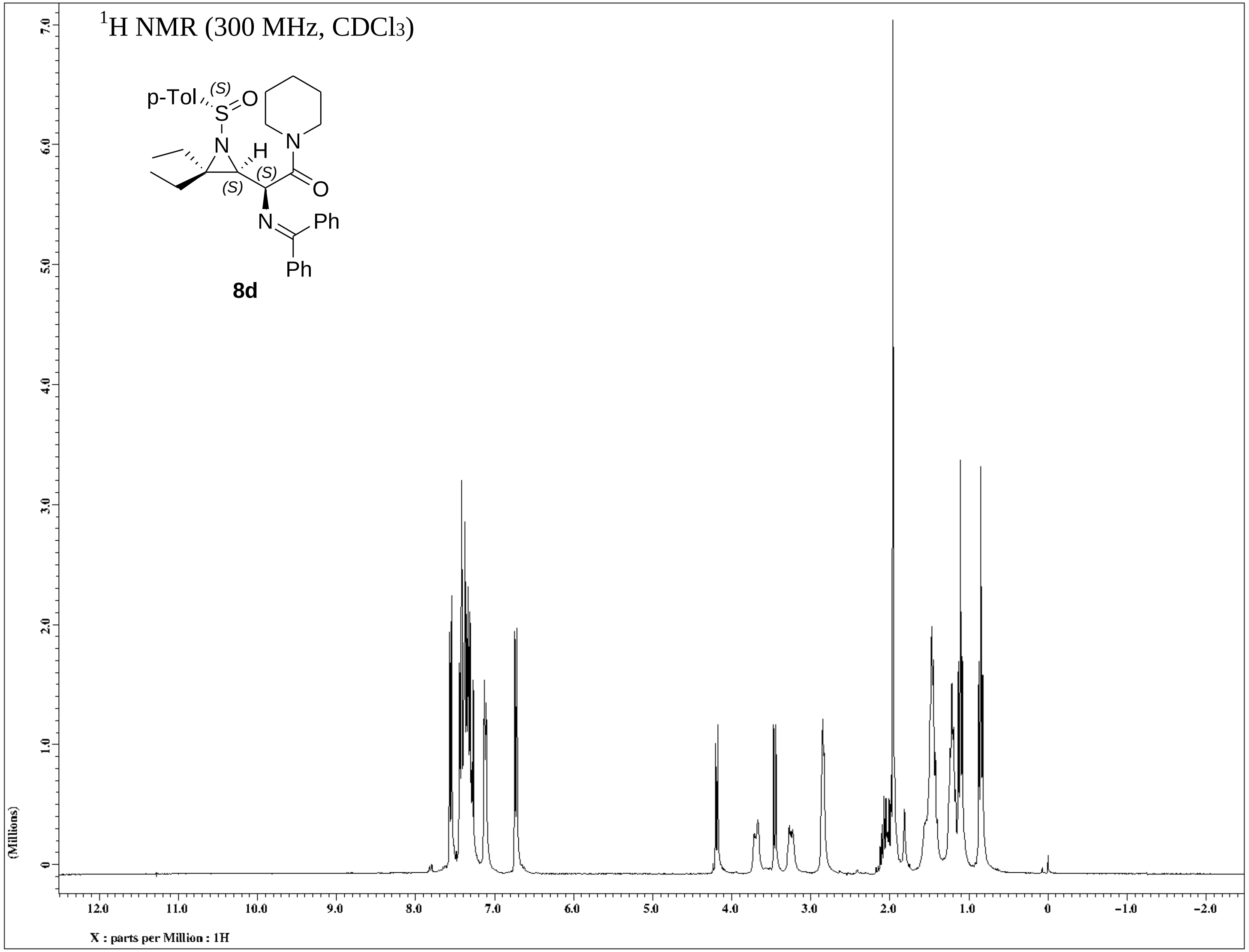
1H NMR (300 MHz, CDCl3)
p-Tol
(S)
S
O
N
H
(S)
(S)
N
O
N
Ph
Ph
8d
X : parts per Million : 1H
(Millions)

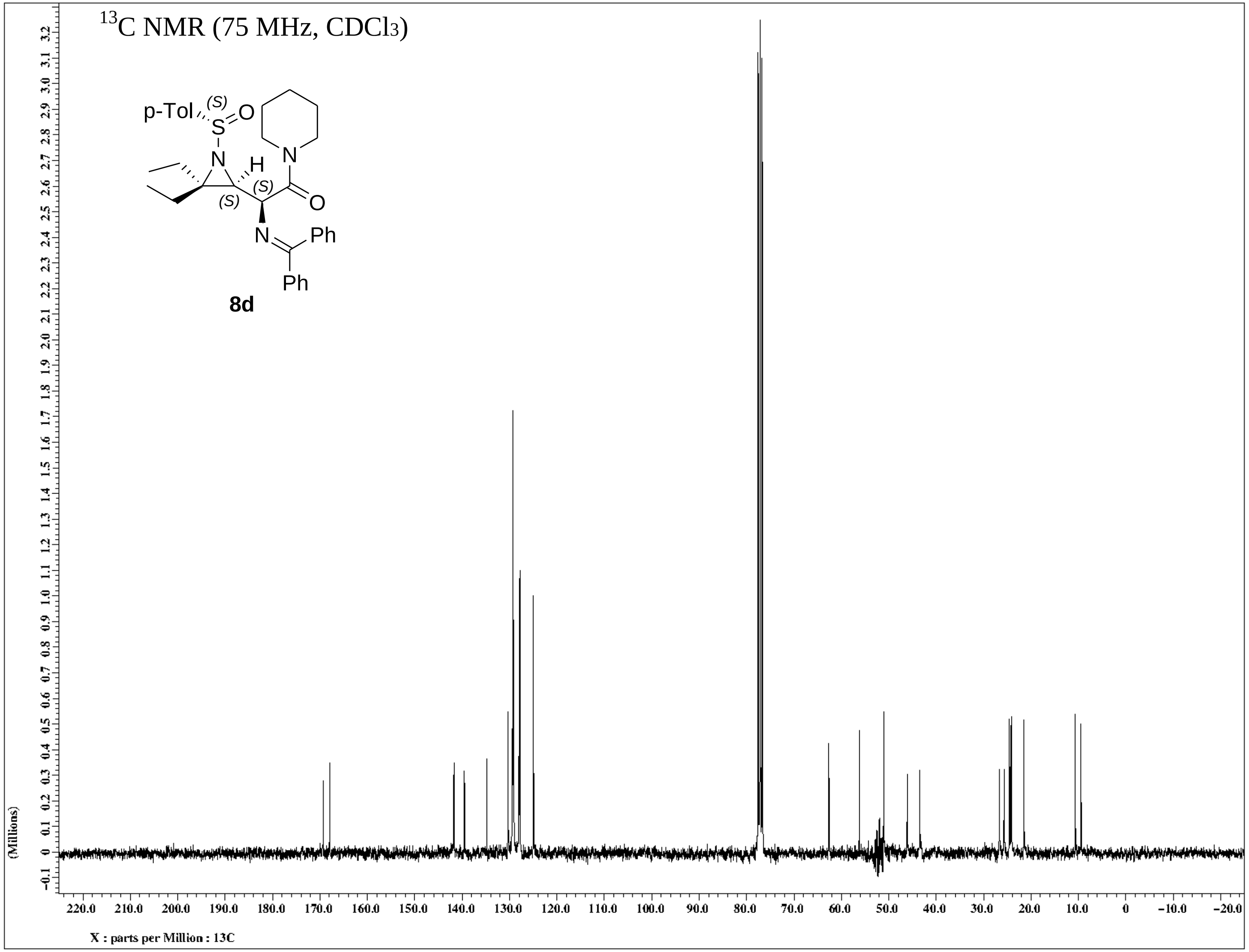

13C NMR (75 MHz, CDCl3)
p-Tol
(S)
S
O
N
H
(S)
(S)
N
O
Ph
Ph
8d
(Millions)
X : parts per Million : 13C

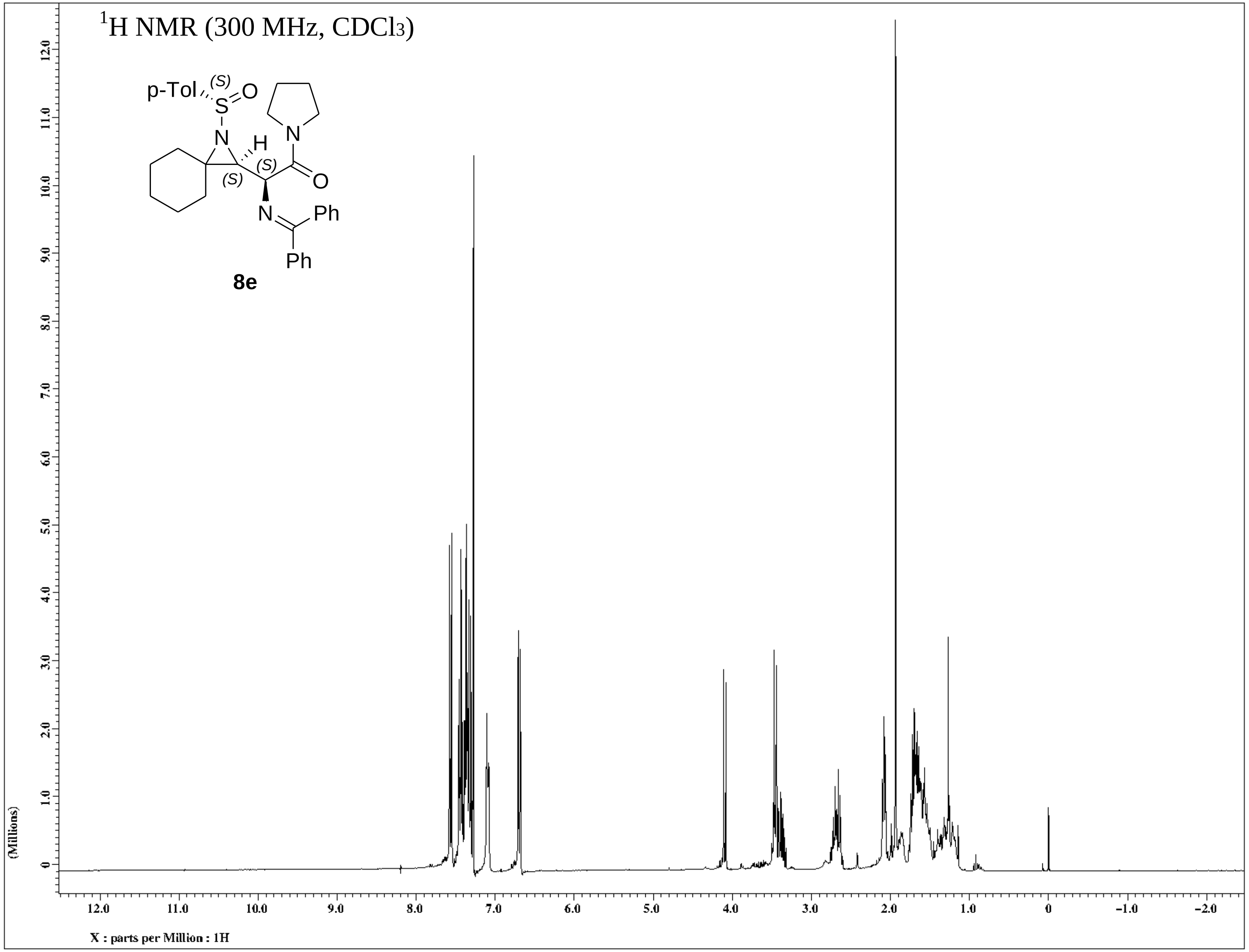

1H NMR (300 MHz, CDCl3)
p-Tol
(S)
S
O
N
H
(S)
(S)
N
O
N
Ph
Ph
8e
(Millions)
X : parts per Million : 1H

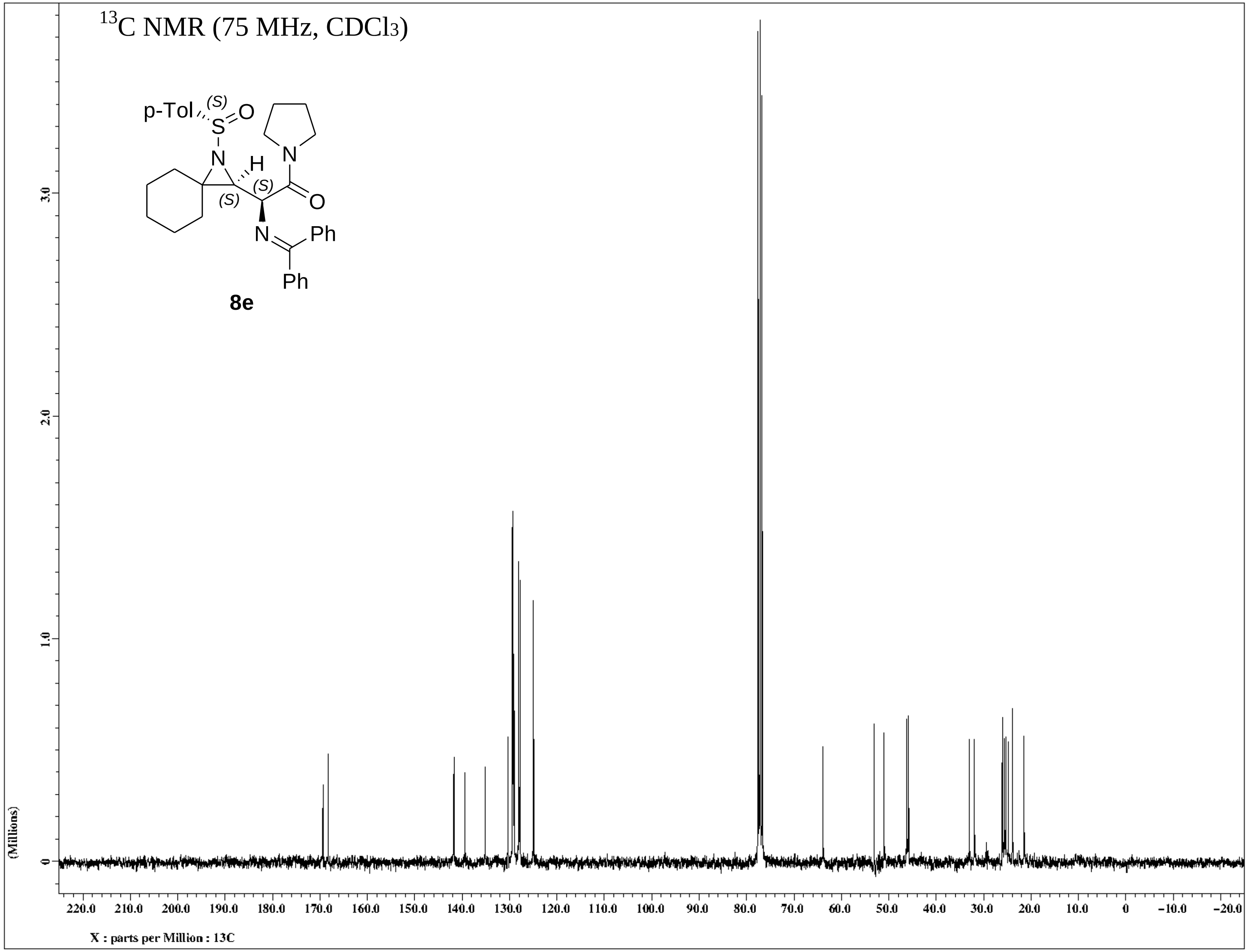

^{13}C NMR (75 MHz, CDCl3)
p-Tol
(S)
S
O
N
H
(S)
(S)
N
O
N
Ph
Ph
8e
(Millions)
3.0
2.0
1.0
0
220.0 210.0 200.0 190.0 180.0 170.0 160.0 150.0 140.0 130.0 120.0 110.0 100.0 90.0 80.0 70.0 60.0 50.0 40.0 30.0 20.0 10.0 0 -10.0 -20.0
X : parts per Million : 13C

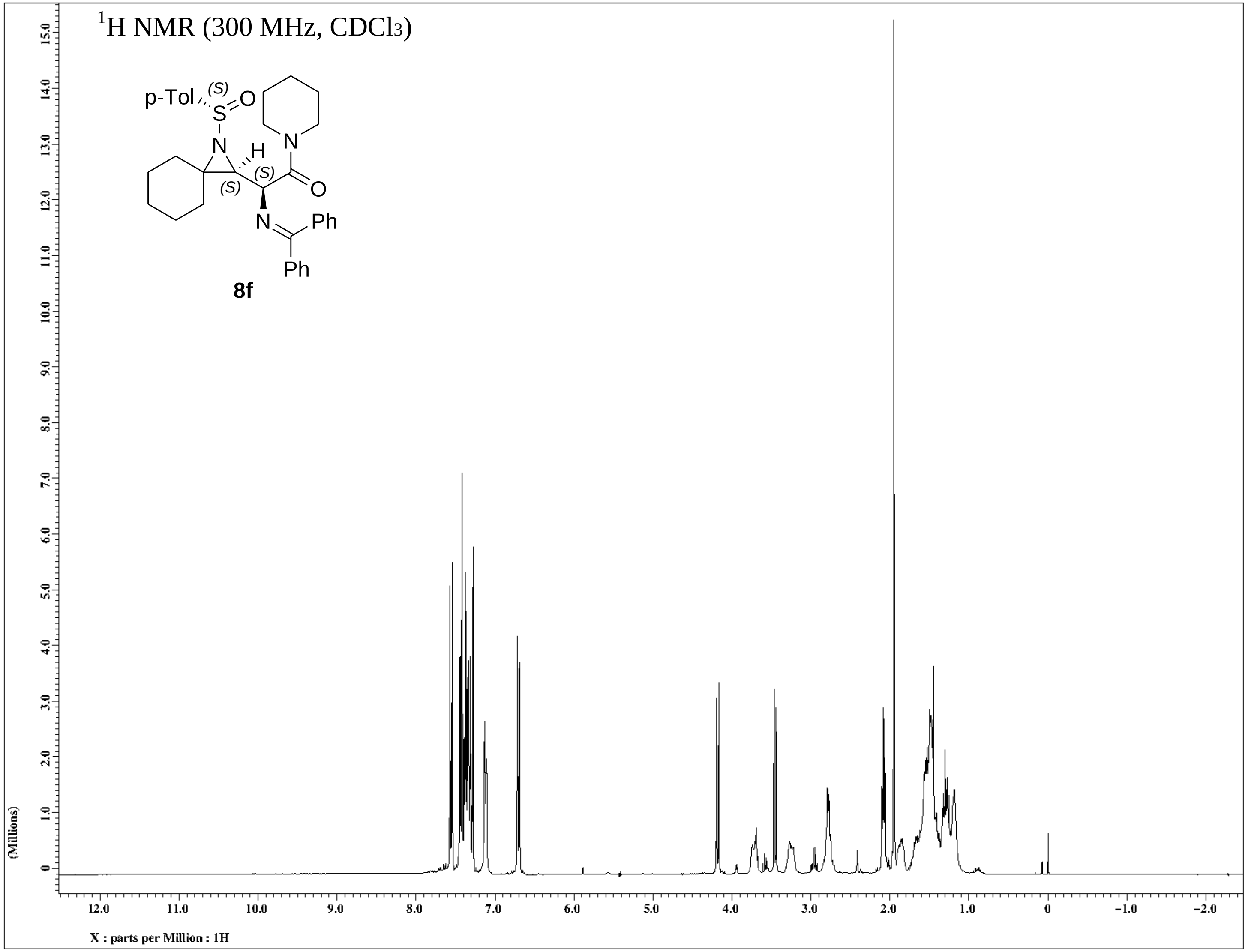

^{1}H NMR (300 MHz, CDCl$_3$)
p-Tol
(S)
S
O
N
H
(S)
(S)
N
O
N
Ph
Ph
8f
(Millions)
X : parts per Million : 1H

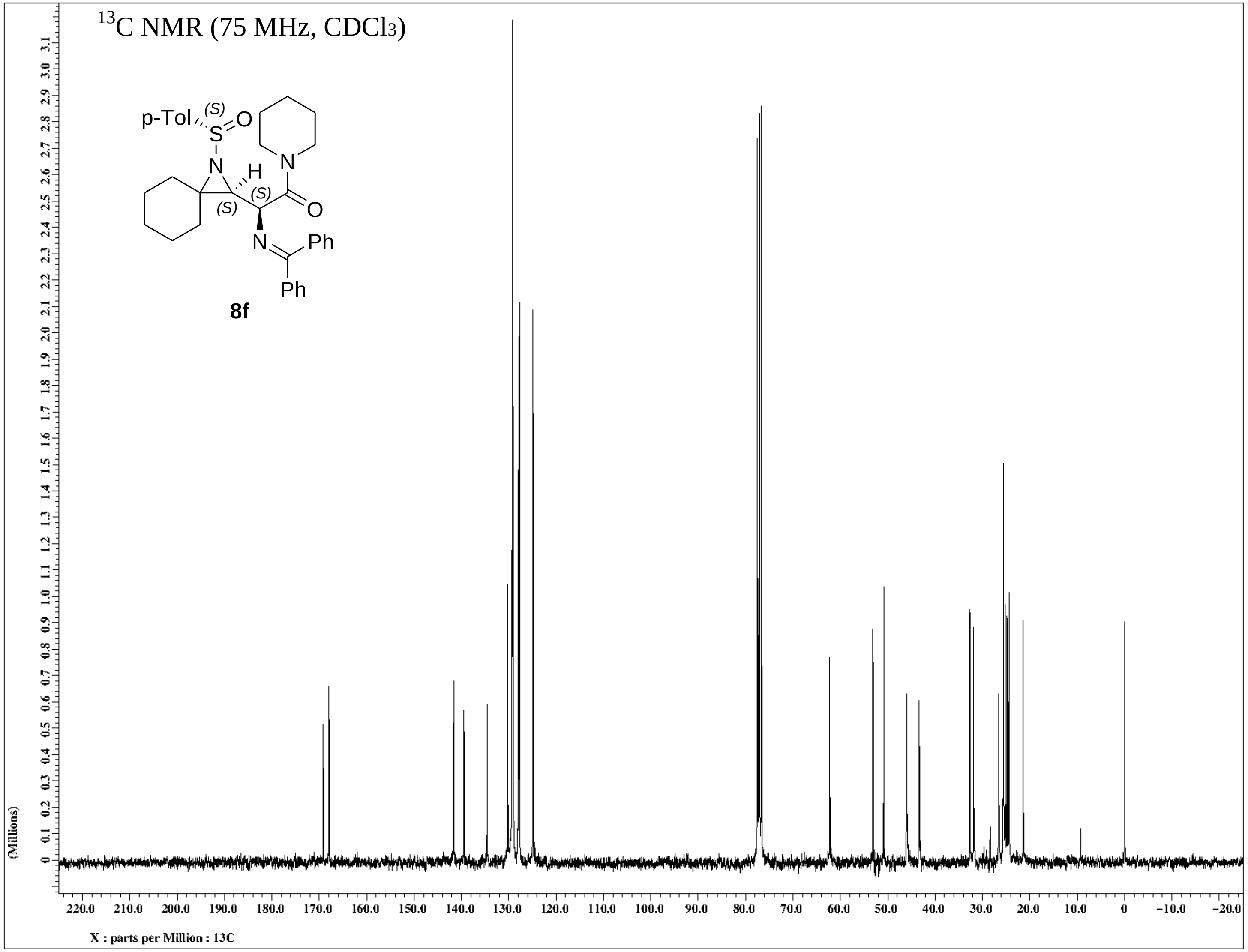

^{13}C NMR (75 MHz, CDCl$_3$)
p-Tol
(S)
S
O
N
H
(S)
(S)
N
O
N
Ph
Ph
8f
(Millions)
X : parts per Million : 13C

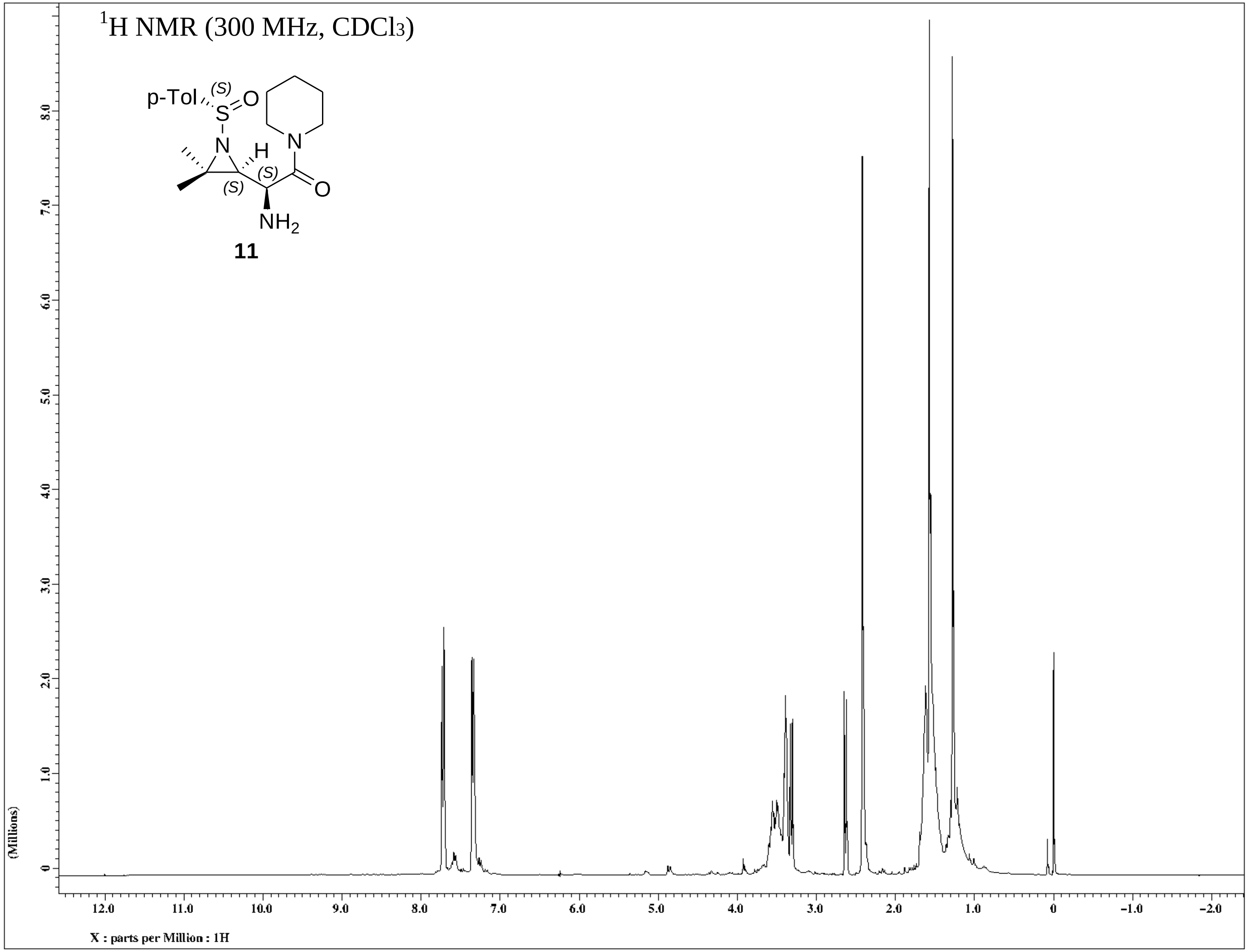
1H NMR (300 MHz, CDCl3)
p-Tol
(S)
S
O
N
H
(S)
(S)
N
O
NH2
11
(Millions)
X : parts per Million : 1H

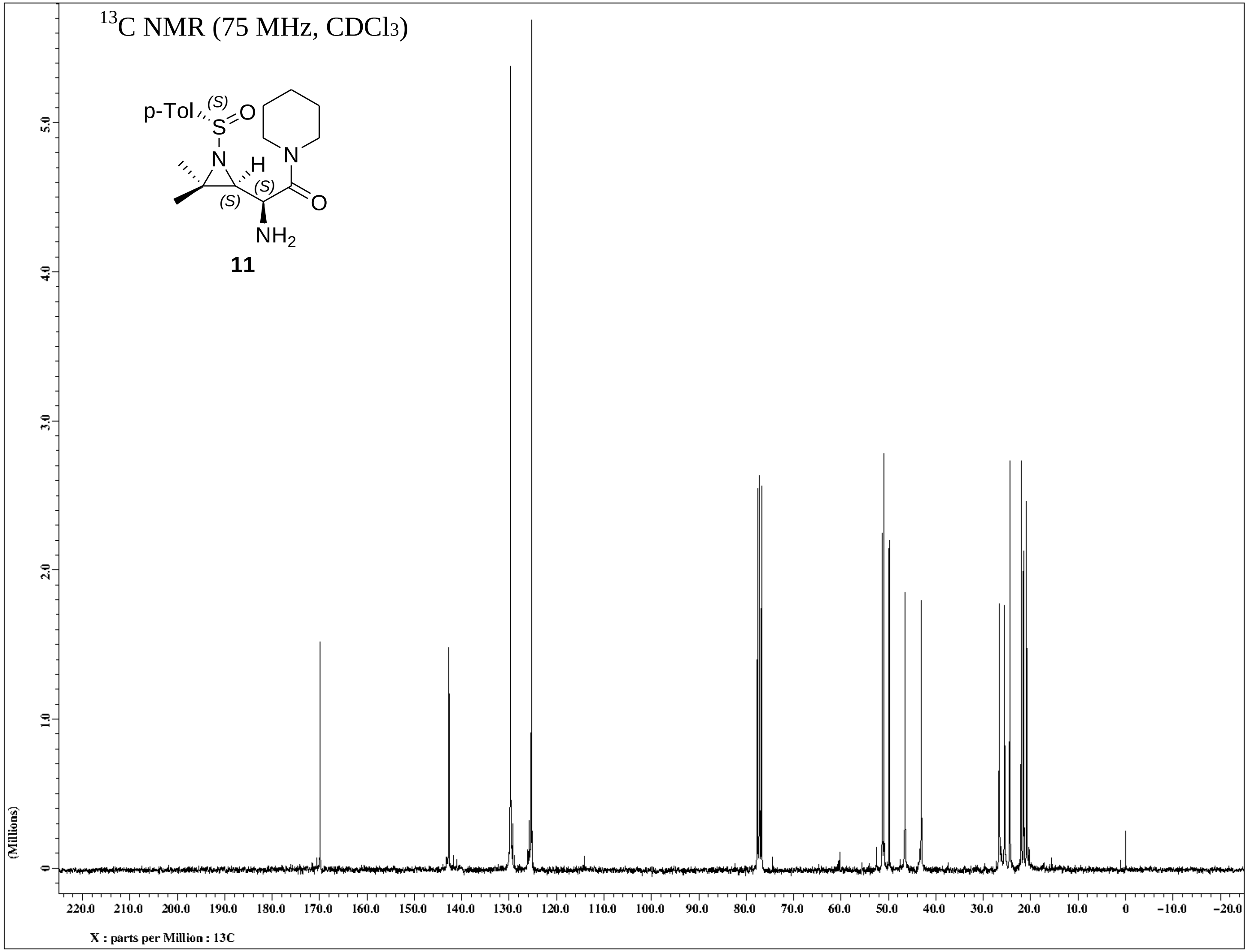

13C NMR (75 MHz, CDCl3)
p-Tol
(S)
S
O
N
H
(S)
(S)
O
N
NH2
11
(Millions)
5.0
4.0
3.0
2.0
1.0
0
220.0 210.0 200.0 190.0 180.0 170.0 160.0 150.0 140.0 130.0 120.0 110.0 100.0 90.0 80.0 70.0 60.0 50.0 40.0 30.0 20.0 10.0 0 -10.0 -20.0
X : parts per Million : 13C

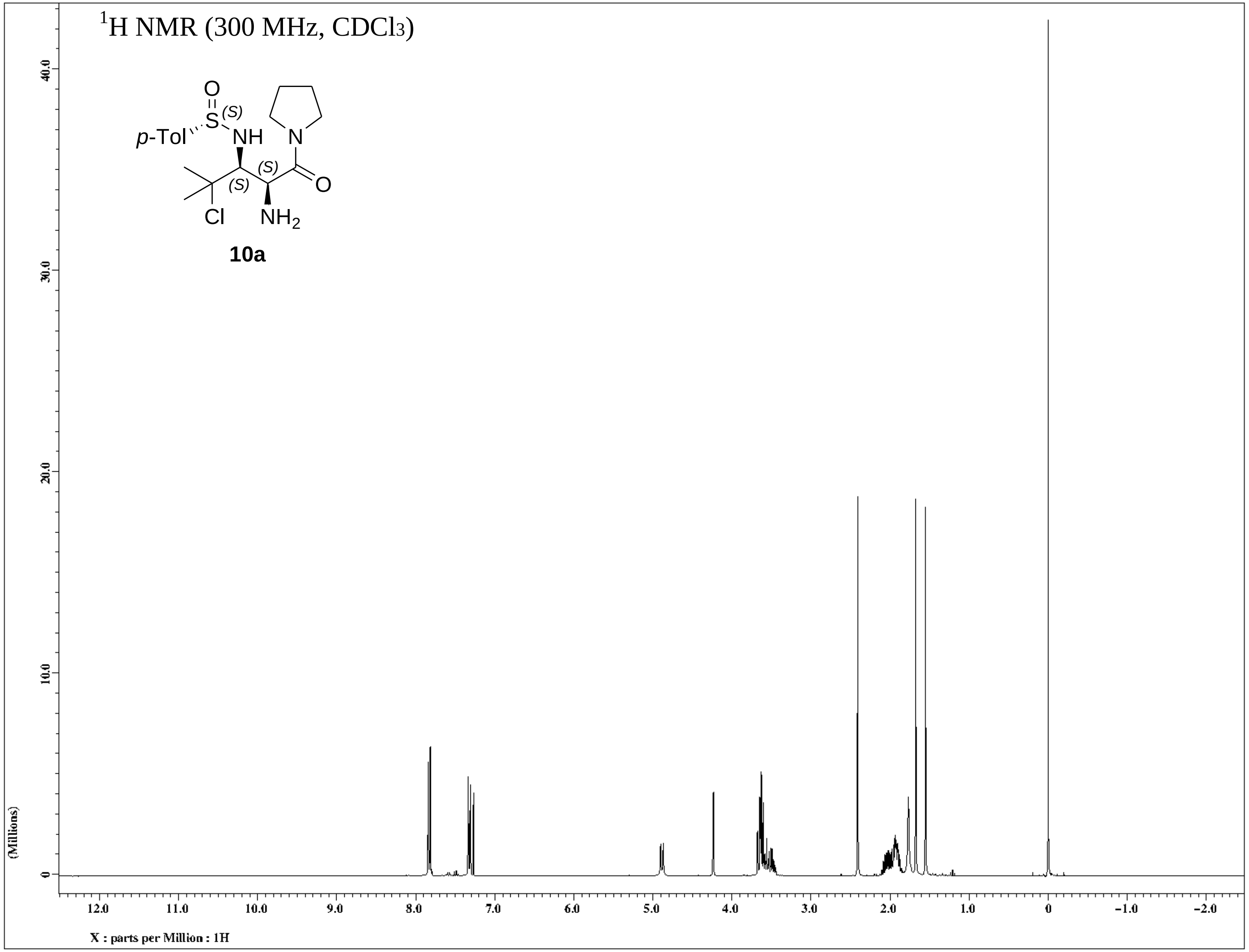

1H NMR (300 MHz, CDCl3)
O
(S)
p-Tol
S
NH
N
(S)
(S)
O
Cl
NH2
10a
(Millions)
40.0
30.0
20.0
10.0
0
12.0
11.0
10.0
9.0
8.0
7.0
6.0
5.0
4.0
3.0
2.0
1.0
0
−1.0
−2.0
X : parts per Million : 1H

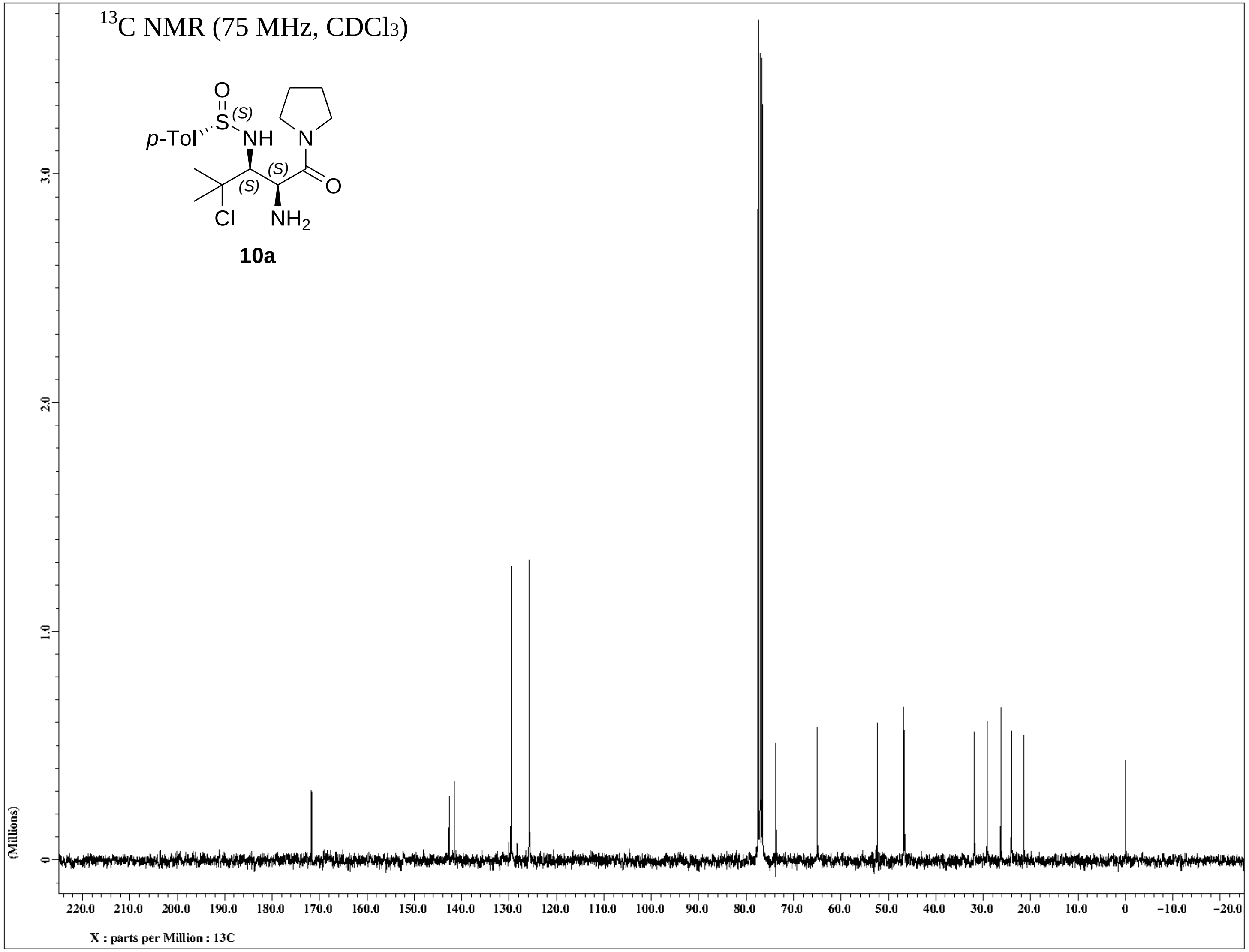

^{13}C NMR (75 MHz, $CDCl_3$)
p-Tol
NH
N
O
Cl
NH_2
(S)
10a
(Millions)
0
1.0
2.0
3.0
220.0 210.0 200.0 190.0 180.0 170.0 160.0 150.0 140.0 130.0 120.0 110.0 100.0 90.0 80.0 70.0 60.0 50.0 40.0 30.0 20.0 10.0 0 −10.0 −20.0
X : parts per Million : 13C

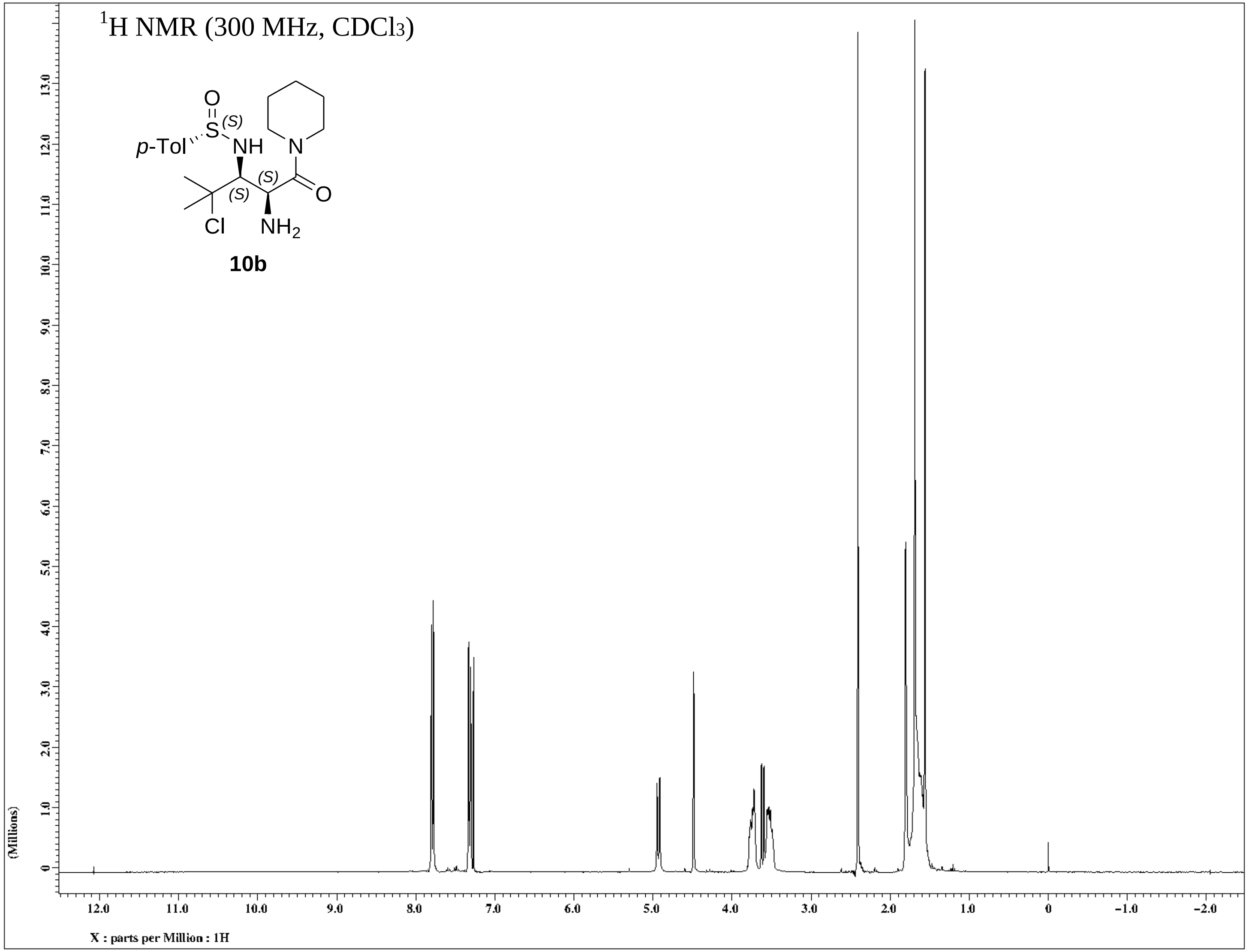

1H NMR (300 MHz, CDCl3)
O
(S)
p-Tol
S
NH
N
(S)
(S)
O
Cl
NH2
10b
(Millions)
X : parts per Million : 1H

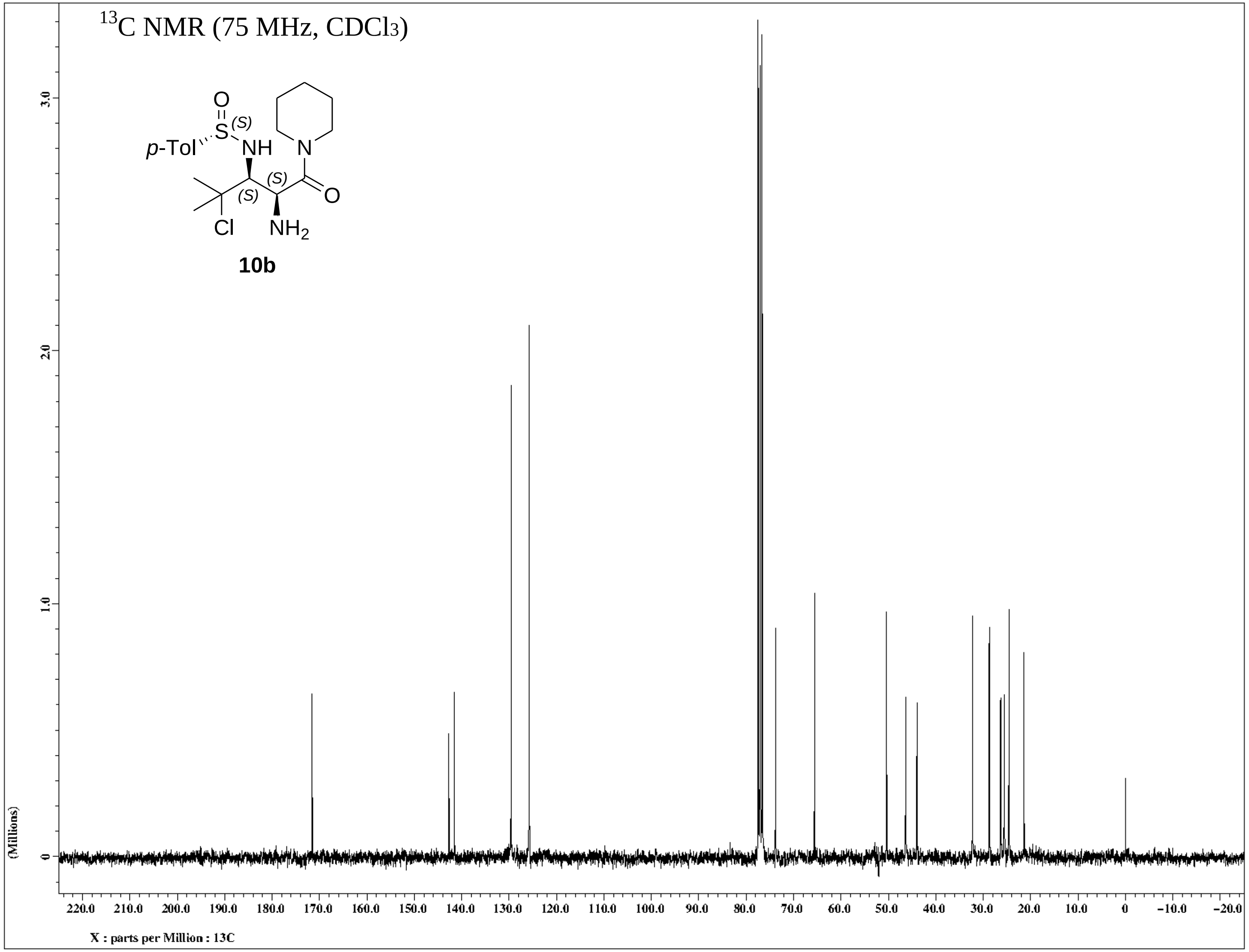

^{13}C NMR (75 MHz, CDCl3)
O
S
(S)
p-Tol
NH
N
(S)
(S)
O
Cl
NH2
10b
3.0
2.0
1.0
0
(Millions)
220.0
210.0
200.0
190.0
180.0
170.0
160.0
150.0
140.0
130.0
120.0
110.0
100.0
90.0
80.0
70.0
60.0
50.0
40.0
30.0
20.0
10.0
0
-10.0
-20.0
X : parts per Million : 13C

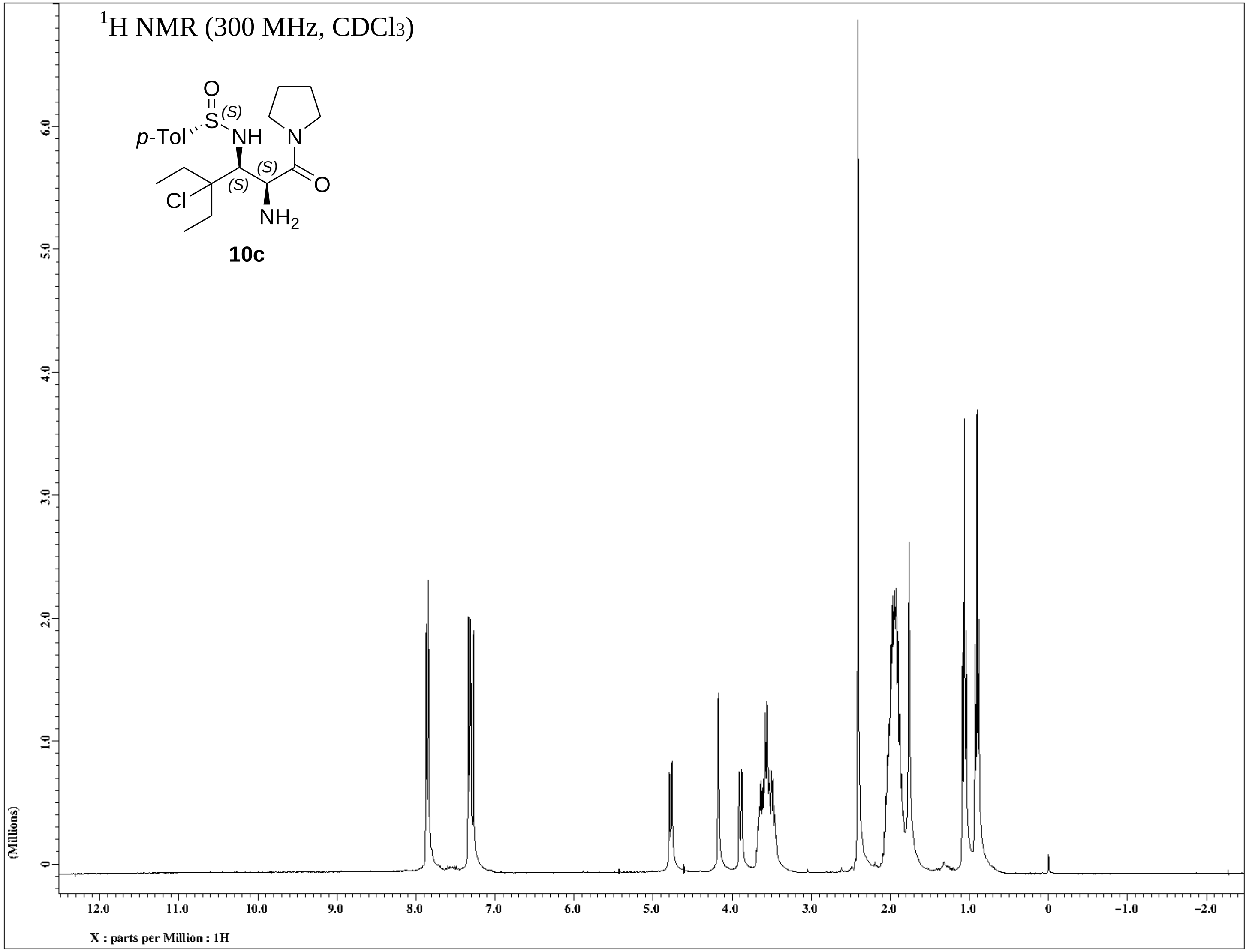
^{1}H NMR (300 MHz, CDCl3)
p-Tol
(S)
NH
Cl
NH$_2$
O
N
10c
(Millions)
X : parts per Million : 1H

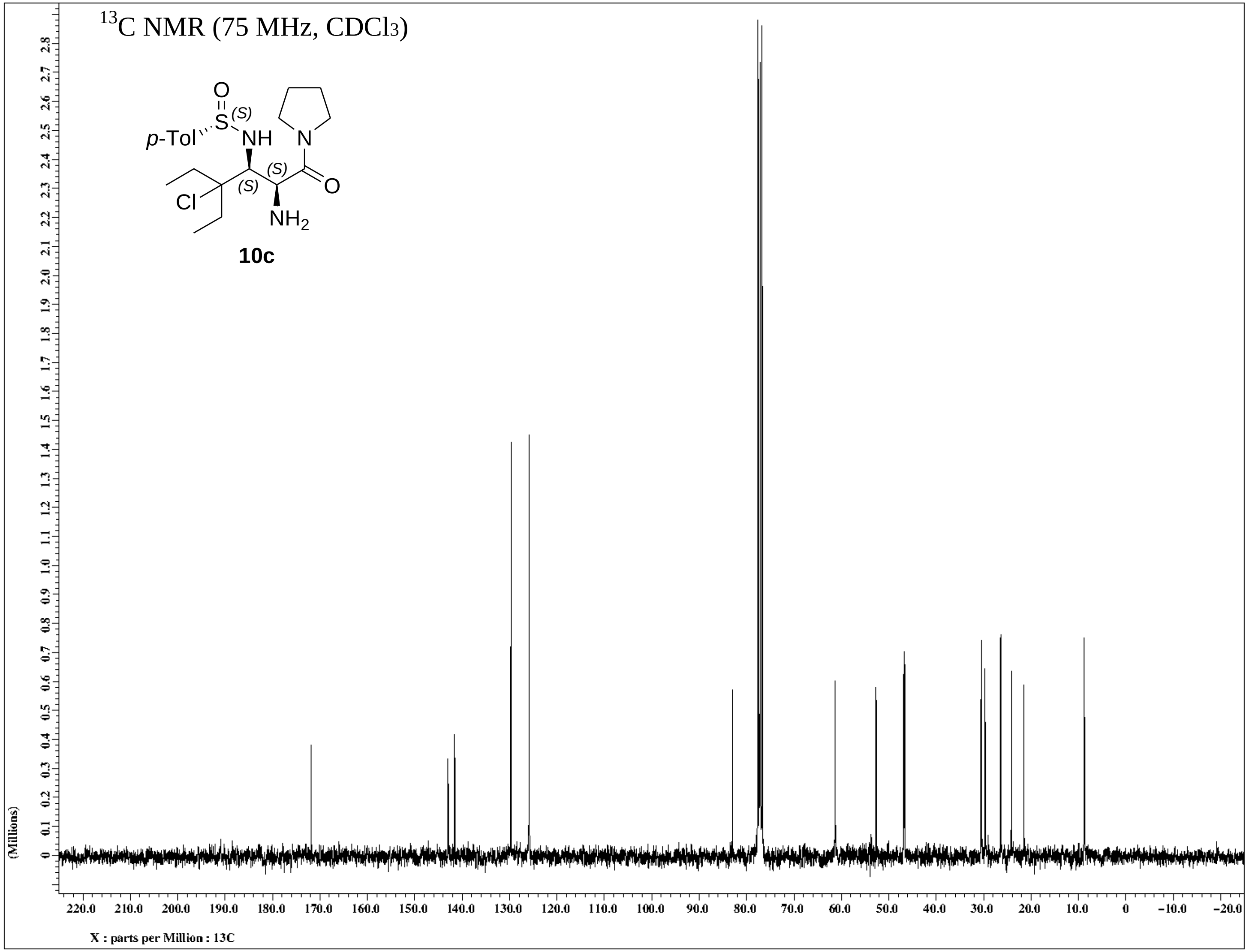

13C NMR (75 MHz, CDCl3)
O
(S)
p-Tol
S
NH
N
(S)
(S)
O
Cl
NH2
10c
(Millions)
X : parts per Million : 13C

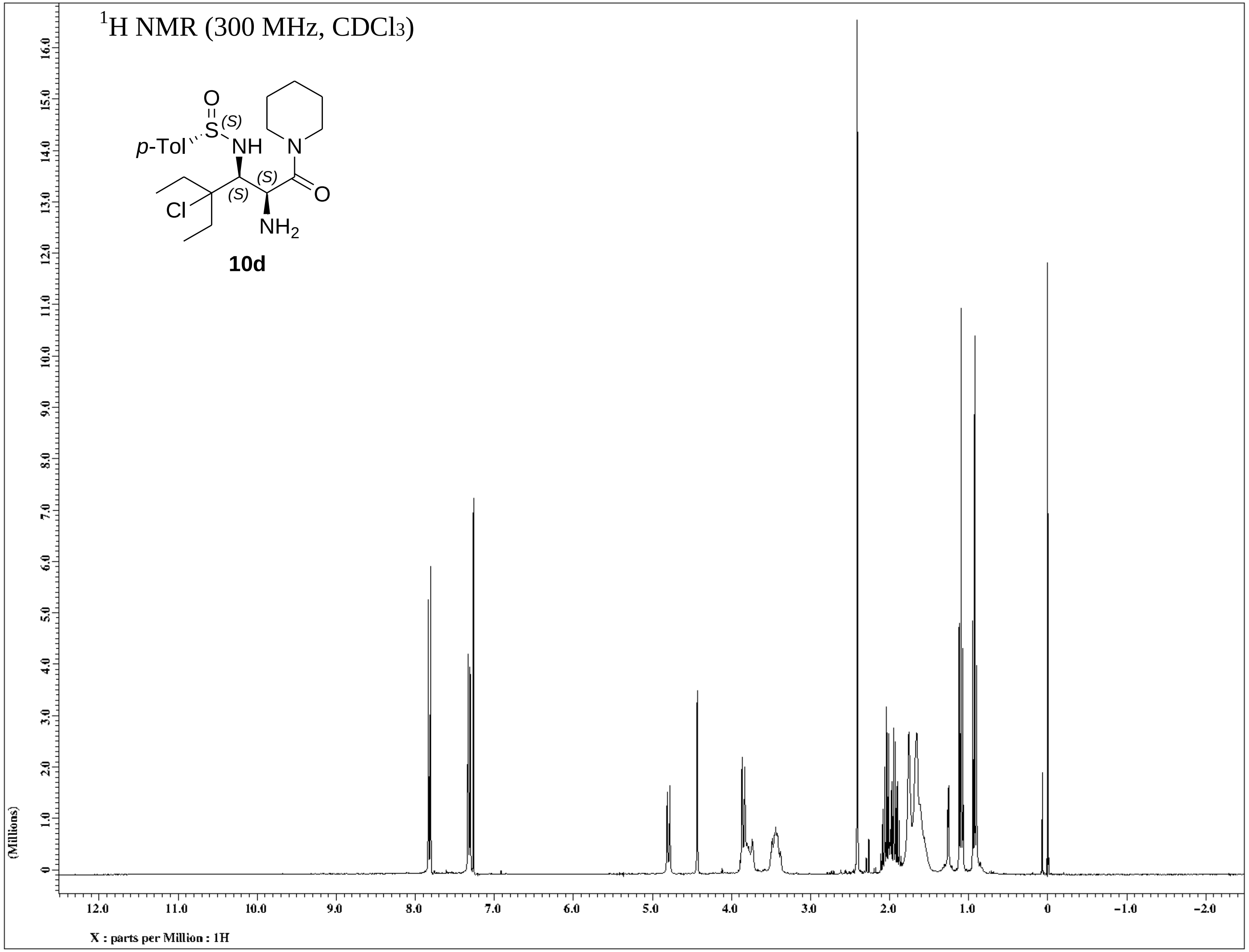

1H NMR (300 MHz, CDCl3)
O
(S)
p-Tol
S
NH
N
(S)
(S)
O
Cl
NH2
10d
(Millions)
X : parts per Million : 1H

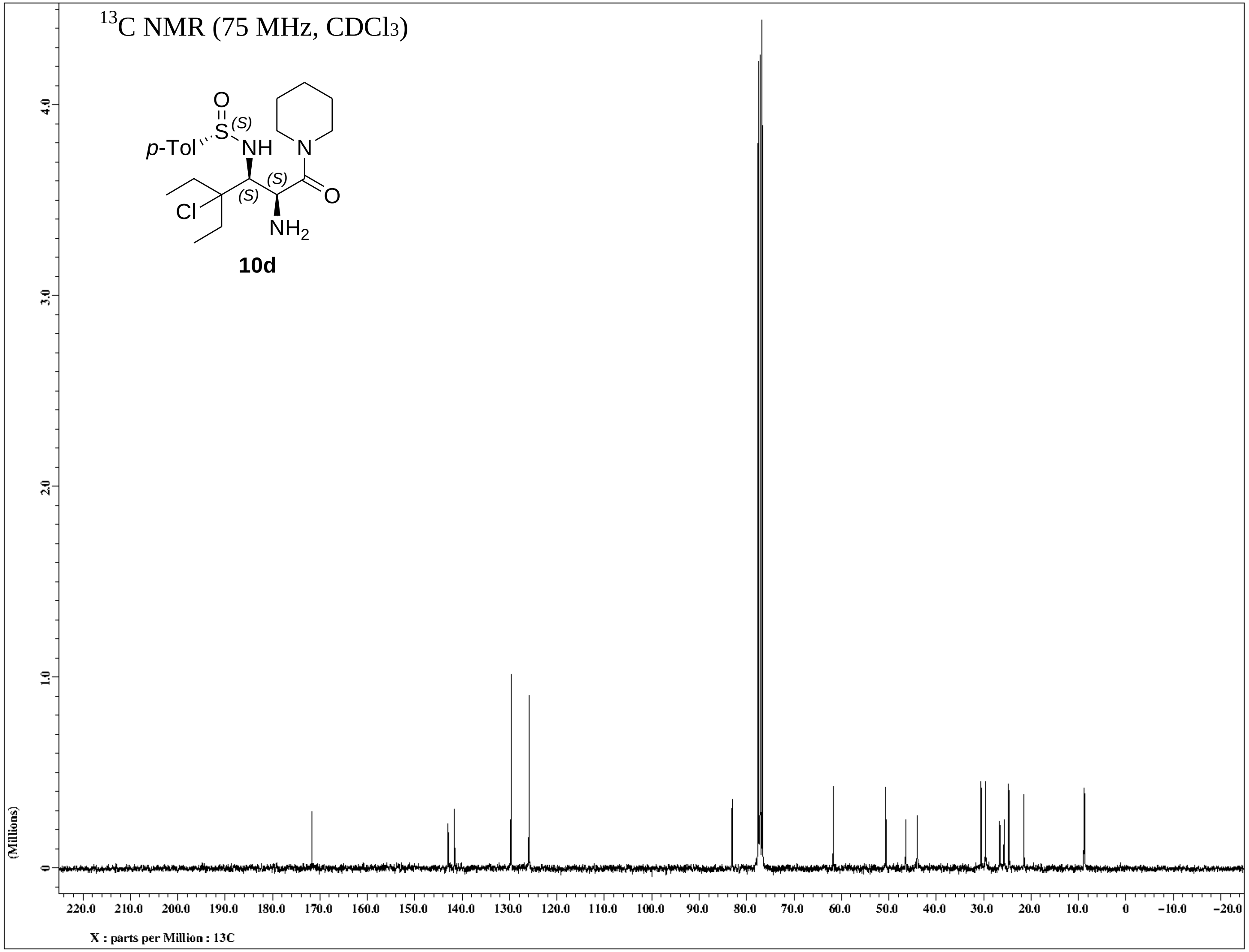
13C NMR (75 MHz, CDCl3)
p-Tol
S
O
(S)
NH
N
O
(S)
(S)
Cl
NH2
10d
(Millions)
4.0
3.0
2.0
1.0
0
220.0 210.0 200.0 190.0 180.0 170.0 160.0 150.0 140.0 130.0 120.0 110.0 100.0 90.0 80.0 70.0 60.0 50.0 40.0 30.0 20.0 10.0 0 -10.0 -20.0
X : parts per Million : 13C

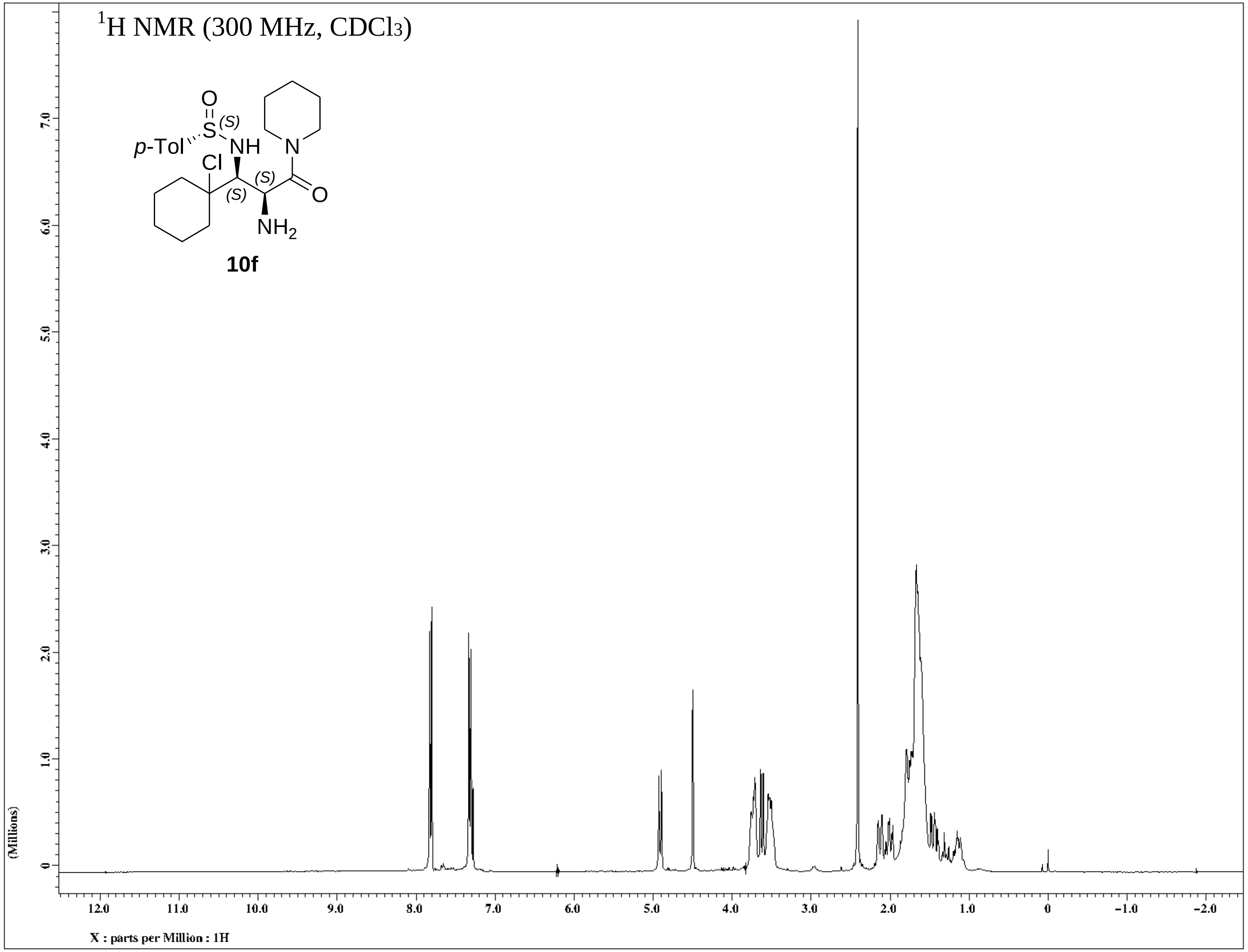

1H NMR (300 MHz, CDCl3)
O
(S)
S
p-Tol
NH
N
Cl
(S)
(S)
O
NH2
10f
7.0
6.0
5.0
4.0
3.0
2.0
1.0
0
(Millions)
12.0
11.0
10.0
9.0
8.0
7.0
6.0
5.0
4.0
3.0
2.0
1.0
0
−1.0
−2.0
X : parts per Million : 1H

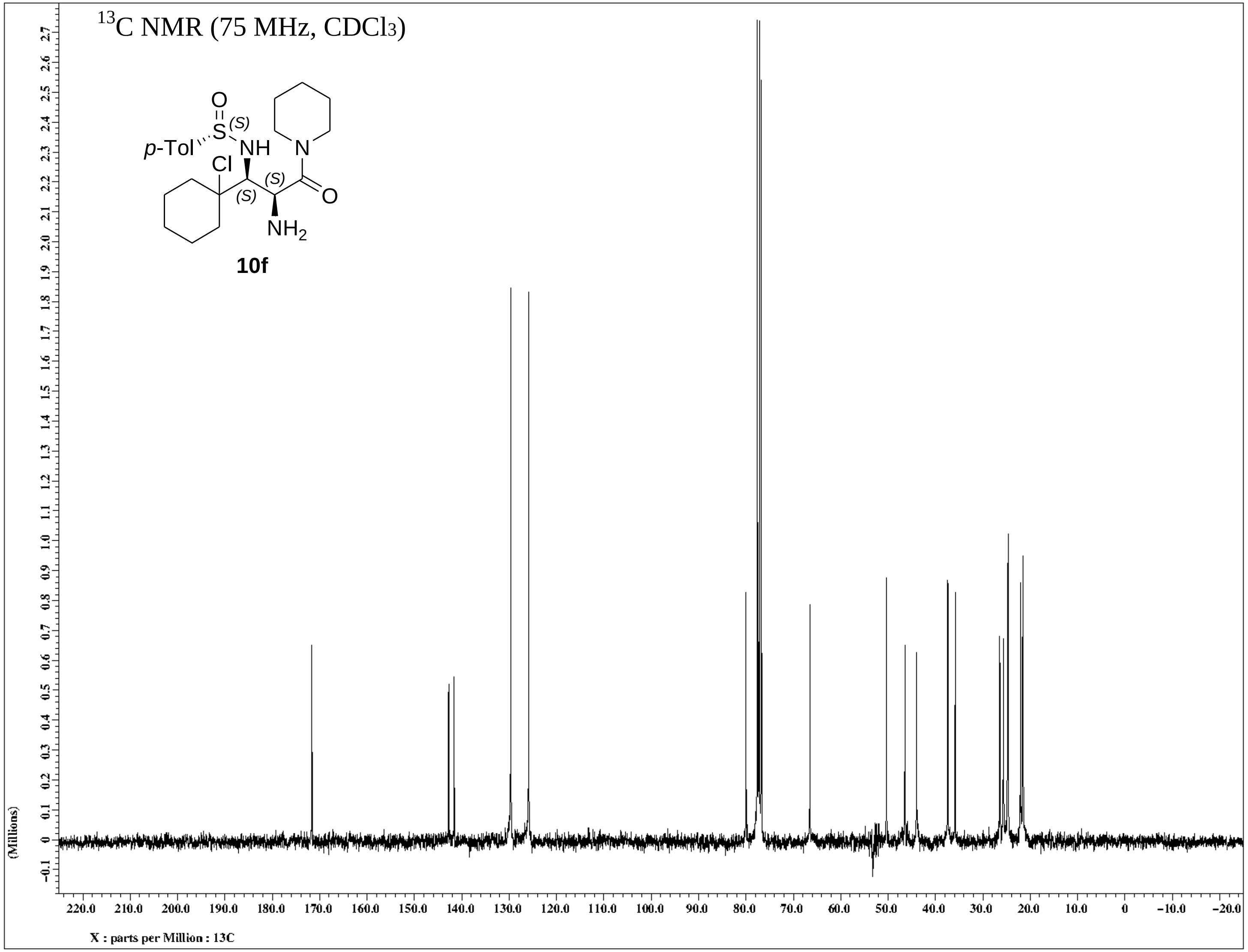

^{13}C NMR (75 MHz, CDCl$_3$)
p-Tol
O
S
(S)
NH
N
Cl
(S)
(S)
O
NH$_2$
10f
(Millions)
X : parts per Million : 13C
220.0 210.0 200.0 190.0 180.0 170.0 160.0 150.0 140.0 130.0 120.0 110.0 100.0 90.0 80.0 70.0 60.0 50.0 40.0 30.0 20.0 10.0 0 −10.0 −20.0

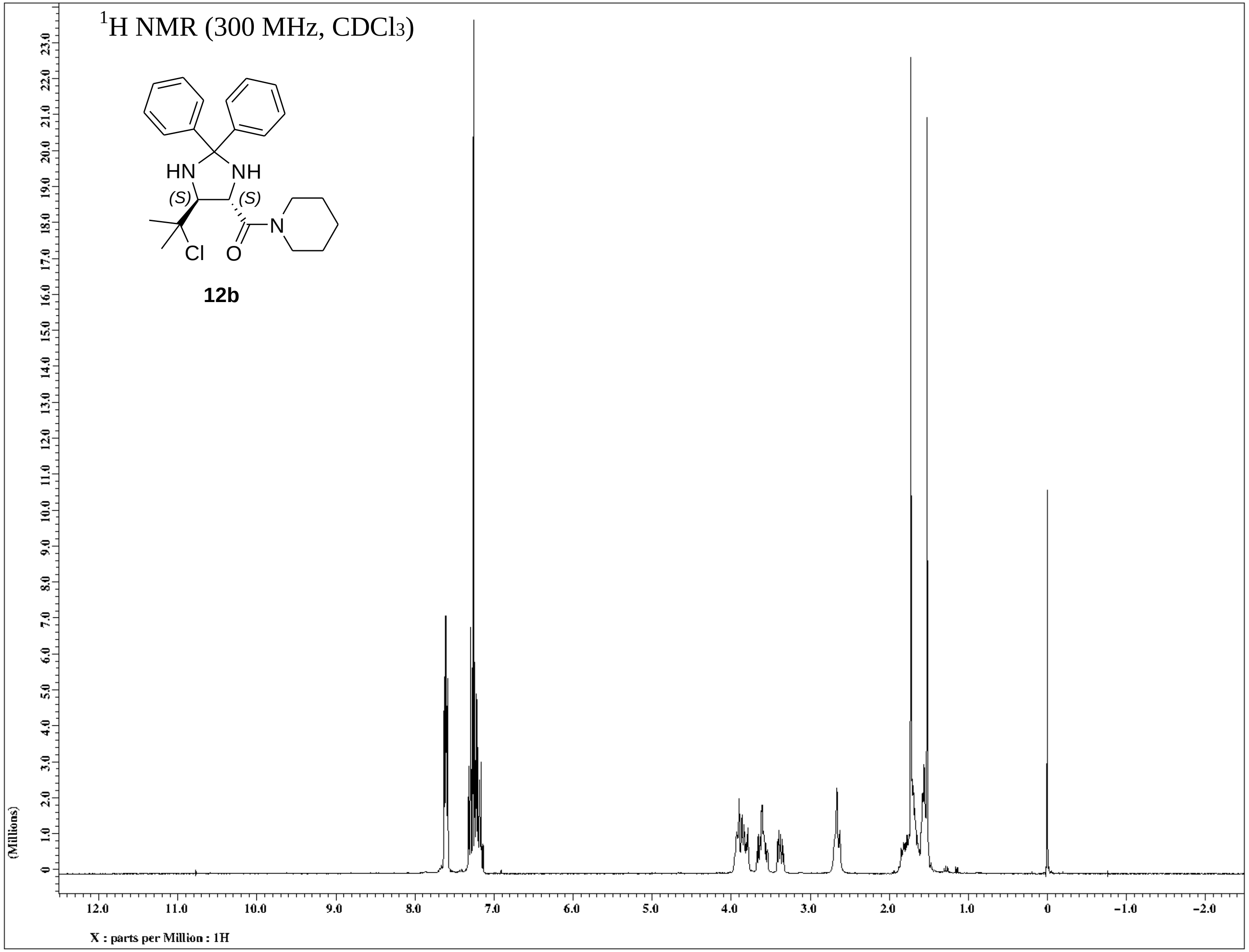
1H NMR (300 MHz, CDCl3)
HN
NH
(S)
(S)
N
Cl
O
12b
(Millions)
X : parts per Million : 1H
12.0
11.0
10.0
9.0
8.0
7.0
6.0
5.0
4.0
3.0
2.0
1.0
0
−1.0
−2.0

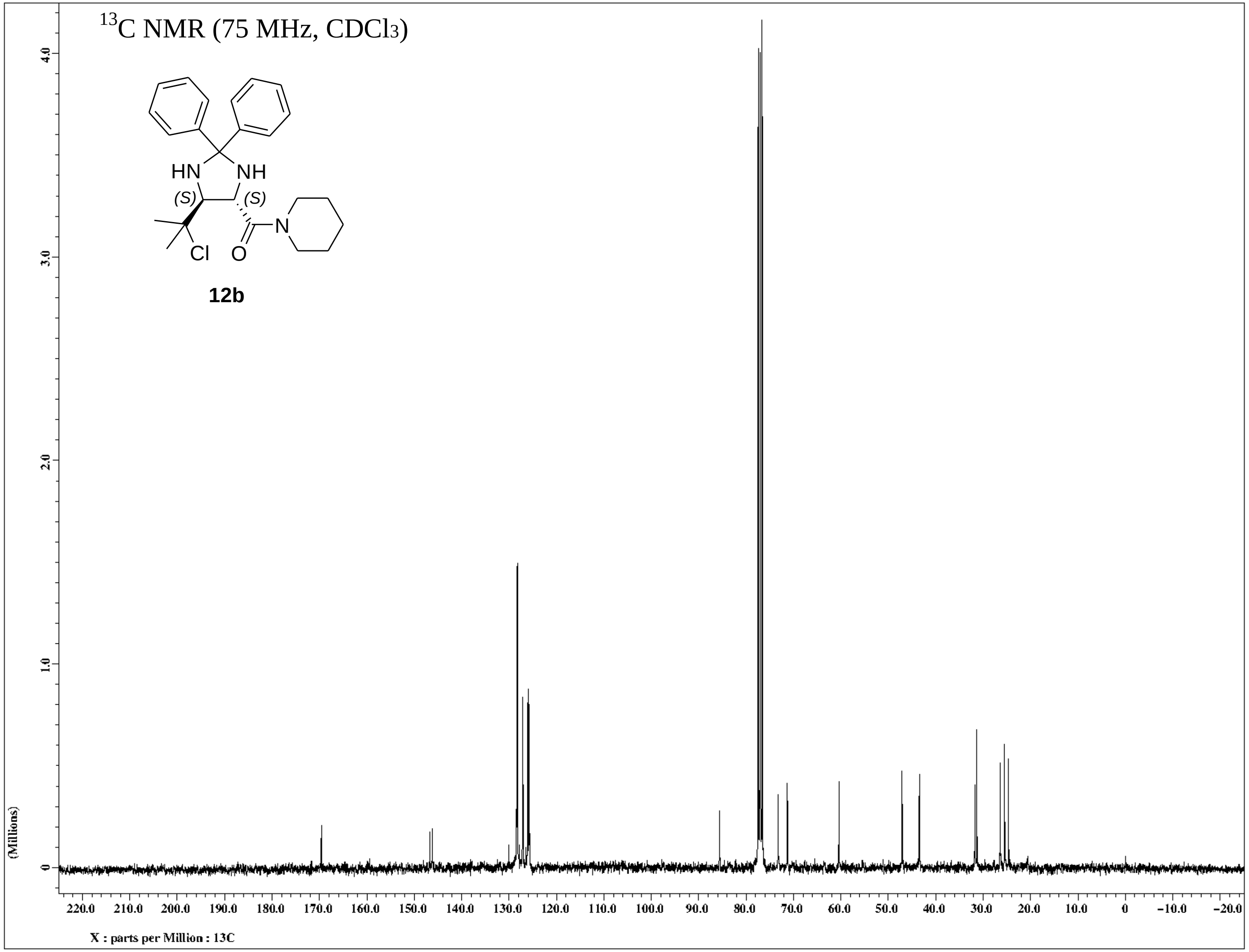
13C NMR (75 MHz, CDCl3)
HN
NH
(S)
(S)
N
Cl
O
12b
4.0
3.0
2.0
1.0
0
(Millions)
220.0 210.0 200.0 190.0 180.0 170.0 160.0 150.0 140.0 130.0 120.0 110.0 100.0 90.0 80.0 70.0 60.0 50.0 40.0 30.0 20.0 10.0 0 −10.0 −20.0
X : parts per Million : 13C

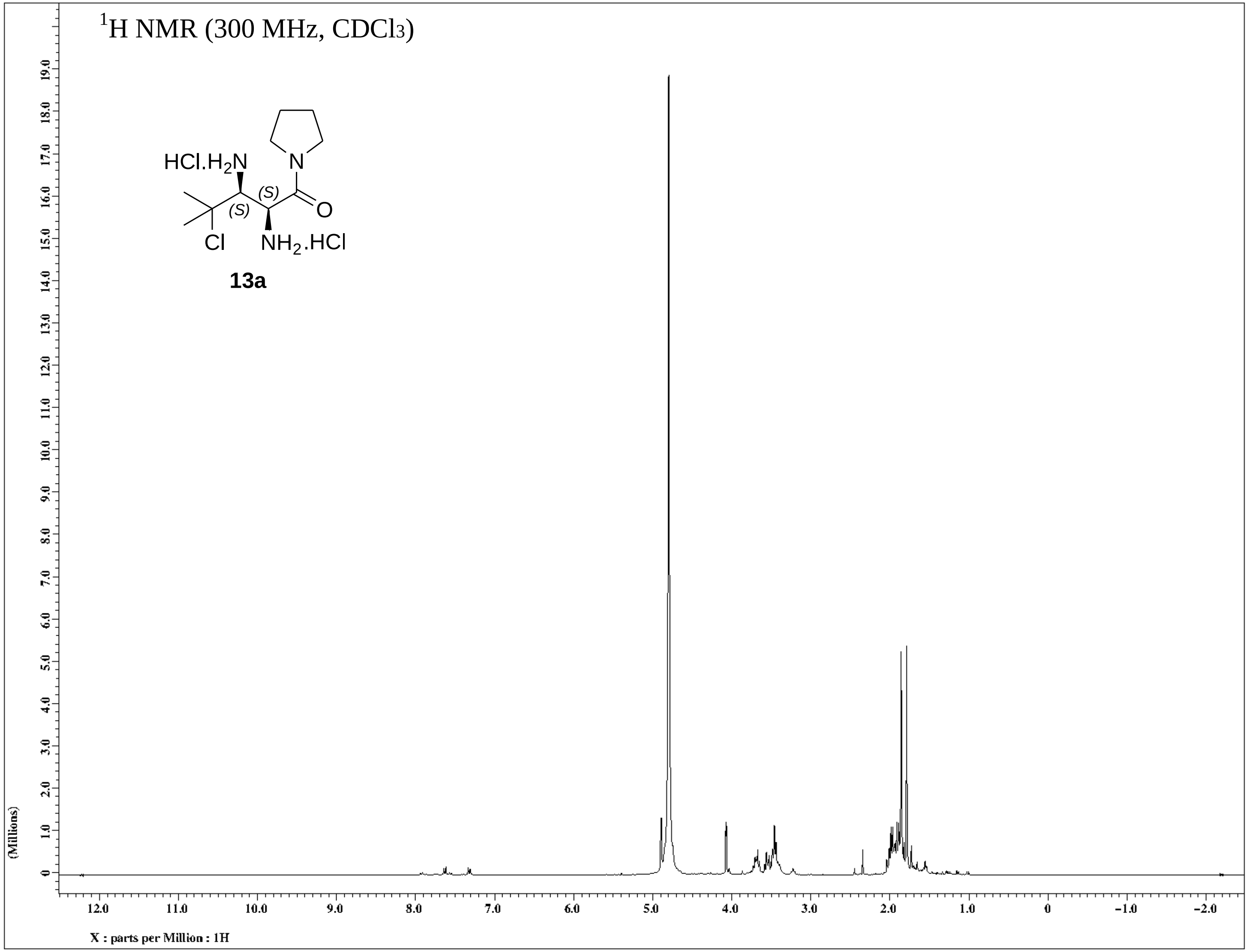

^{1}H NMR (300 MHz, CDCl$_3$)
HCl.H$_2$N
(S)
(S)
N
O
Cl
NH$_2$.HCl
13a
(Millions)
X : parts per Million : 1H

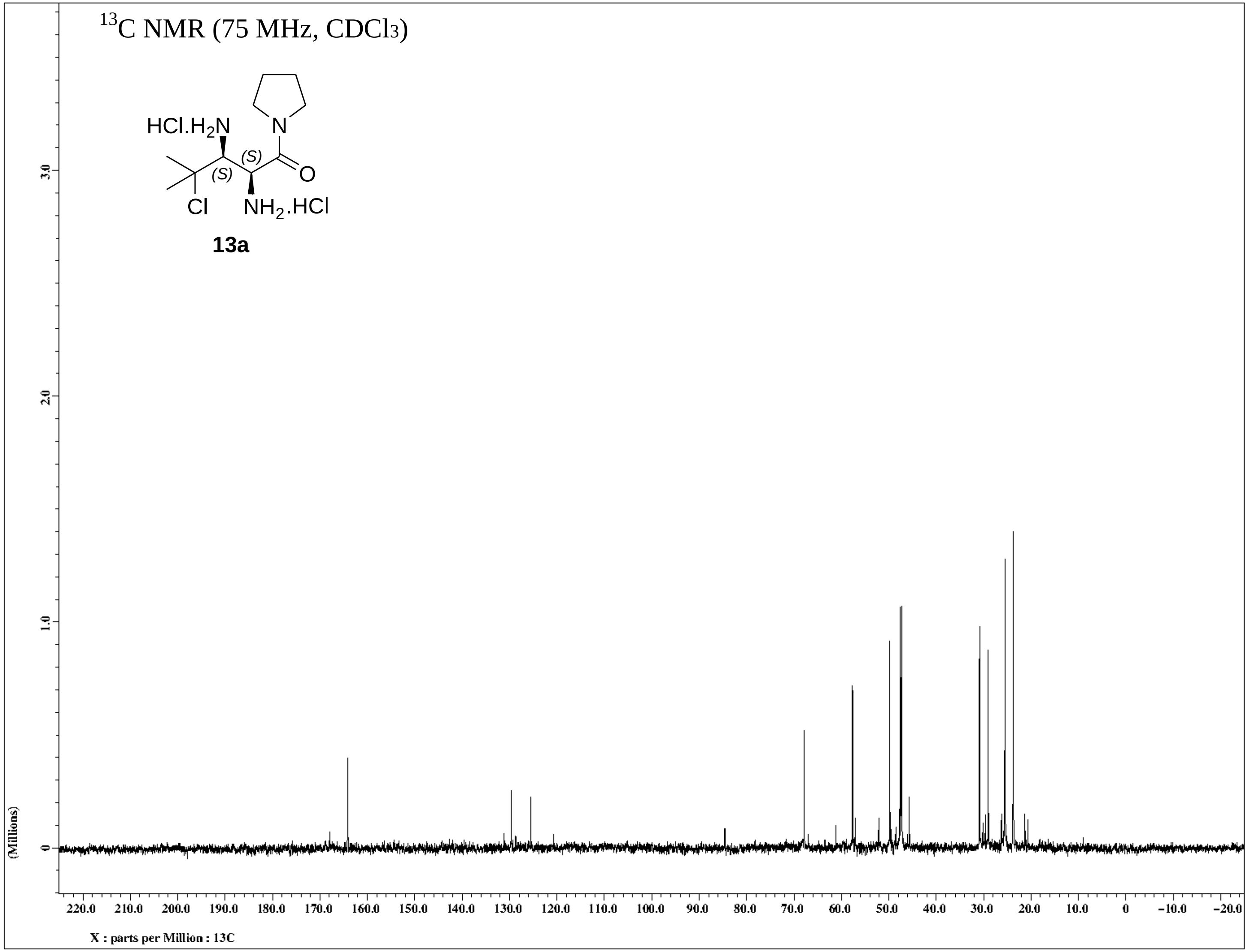

^{13}C NMR (75 MHz, CDCl3)
HCl.H2N
N
(S)
(S)
O
Cl
NH2.HCl
13a
(Millions)
X : parts per Million : 13C
